# A mixed chirality α-helix in a stapled bicyclic and a linear antimicrobial peptide revealed by X-ray crystallography†

**DOI:** 10.1039/d1cb00124h

**Published:** 2021-08-20

**Authors:** Stéphane Baeriswyl, Hippolyte Personne, Ivan Di Bonaventura, Thilo Köhler, Christian van Delden, Achim Stocker, Sacha Javor, Jean-Louis Reymond

**Affiliations:** Department of Chemistry, Biochemistry and Pharmaceutical Sciences, University of Bern Freiestrasse 3 3012 Bern Switzerland jean-louis.reymond@unibe.ch; Department of Microbiology and Molecular Medicine, University of Geneva, Service of Infectious Diseases, University Hospital of Geneva Geneva Switzerland

## Abstract

The peptide α-helix is right-handed when containing amino acids with l-chirality, and left-handed with d-chirality, however mixed chirality peptides generally do not form α-helices unless a helix inducer such as the non-natural residue amino-isobutyric acid is used. Herein we report the first X-ray crystal structures of mixed chirality α-helices in short peptides comprising only natural residues as the example of a stapled bicyclic and a linear membrane disruptive amphiphilic antimicrobial peptide (AMP) containing seven l- and four d-residues, as complexes of fucosylated analogs with the bacterial lectin LecB. The mixed chirality α-helices are superimposable onto the homochiral α-helices and form under similar conditions as shown by CD spectra and MD simulations but non-hemolytic and resistant to proteolysis. The observation of a mixed chirality α-helix with only natural residues in the protein environment of LecB suggests a vast unexplored territory of α-helical mixed chirality sequences and their possible use for optimizing bioactive α-helical peptides.

## Abstract 2

We report the first X-ray crystal structures of mixed chirality α-helices comprising only natural residues as the example of bicyclic and linear membrane disruptive amphiphilic antimicrobial peptides containing seven l- and four d-residues.

## Graphical abstract

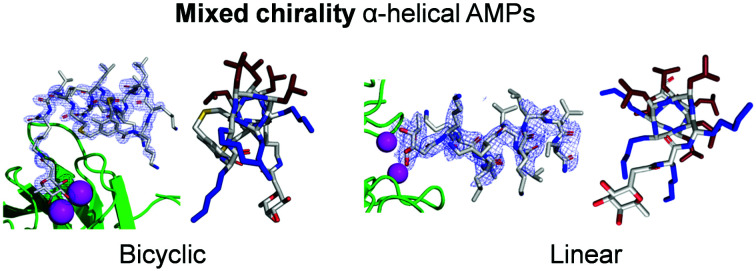

## Introduction

The primary amino acid sequence of a peptide or protein determines whether and which type of folded conformation it may form.^1^ However, conformational preferences are almost exclusively documented and understood in the context of homochiral, all l- or all d-sequences, which form α-helices and β-sheets as canonical secondary structures. Studies with mixed chirality peptides have shown that they are generally not compatible with α-helical folding,^2–4^ or form alternative secondary structures such as β-helices, α-strands, various types of β-turns, as well as intermolecular aggregates.^5–13^ However, α-helical mixed chirality sequences have been directly observed by X-ray crystallography in peptides incorporating the non-natural residue amino-isobutyric acid as the α-helix inducer.^14–17^ Incorporation of one or two d-residues in an α-helical l-peptide sometimes preserves helicity, as recently documented by X-ray crystallography for a macrocyclic peptide inhibitor of the p53–MDM2 interaction,^18^ and by CD-spectroscopy in a stapled bicyclic peptide inhibitor of the EGF–EGFR complex^19^ and a stapled peptide KRAS inhibitor.^20^ Note that mixed chirality 10/12 helices have been reported for β-peptides.^21^

In the case of antimicrobial peptides (AMPs), which are considered as an opportunity to address the public health threat of ESKAPE pathogens due to their ability to kill multidrug-resistant bacteria,^22–26^ studies on short amphiphilic α-helical sequences have shown that mixed chirality analogs lose the ability to form α-helices, but often retain their antimicrobial activity while hemolysis and protease sensitivity are reduced.^27–31^ In these studies, the deconvolution of relatively broad Fourier-transform infrared (FTIR) spectroscopy bands in the amide region suggested that some of the mixed chirality AMPs adopted random coil/α-helix/3_10_-helix secondary structures in the membrane environment.^32–35^ On the other hand, a recent atomic force microscopy study with random heterochiral AMP sequences in supported lipid bilayers suggested that these random AMPs do not fold, which would be compatible with a “carpet” type mechanism of membrane disruption suitable for the bactericidal effect, but not with pore formation required for hemolysis.^36^ Our own recent studies with stereorandomized AMPs showed that the ensemble of all possible mixed chirality versions of α-helical AMPs do not have antibacterial or membrane disruptive activity, suggesting that amphiphilic folding is not possible with most mixed chirality sequences.^37^

**Fig. 1:**
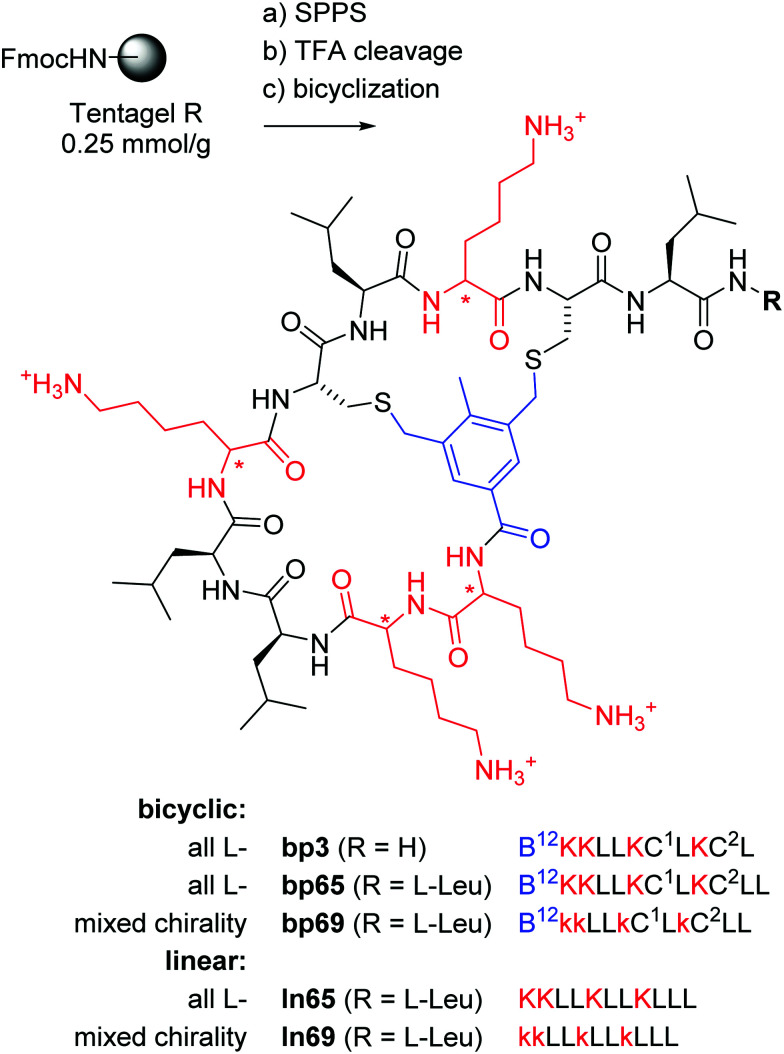
Synthesis and structure of homochiral and mixed chirality AMBPs bp3, bp65, bp69, and the linear AMPs ln65 and ln69. Conditions: (a) solid-phase peptide synthesis: (i) piperidine/DMF 1 : 4, 2 × 20 min, 50 °C, (ii) FmocAAOH (last coupling for bicyclic: 3,5-bis(chloromethyl)-4-methylbenzoic acid), DIC/oxyma, DMF, 50 °C; (b) TFA/TIS/DODT/H_2_O 94 : 2.5 : 2.5 : 1, v/v/v/v, 3 h; (c) for bicyclic: H_2_O/MeCN (50 : 50, v/v), KI (1 eq.), DIEA (5 eq.), 1 h. The line notation for bicyclic structures uses single letter codes for amino acids and the SMILES convention for cyclization points,^49,50^ B = 3,5-bis(methylene)-toluoyl. All products were purified by preparative RP-HPLC.

In the context of developing AMPs with branched topologies against multi-drug resistant Gram-negative bacteria,^38–40^ we recently identified antimicrobial bicyclic peptides (AMBPs) showing potent membrane disruptive and antibacterial activities.^41,42^ However, all our AMBPs contained substantial acyclic portions and eluded precise structure determination. Capitalizing on our previous success in determining the structure of short peptides and peptide dendrimers by X-ray crystallography of lectin complexes,^43–48^ we set out to obtain a direct structural insight into the bicyclic architecture of our AMBPs. Our starting point of choice was bp3, a weakly active but short AMBP prepared during a virtual screening guided campaign, in which all residues except its C-terminus resided within the loops (Fig. 1).^42^

Herein we report that optimization of bp3 by sequence and residue chirality variations followed by structural studies led us to the first X-ray crystal structure of an AMPB in the form of bp65. The structure of bp65 features an α-helical fold essentially identical to that of the corresponding linear peptide ln65 but comprising a bicyclic helix staple. More importantly, our study revealed the first X-ray crystal structure of mixed chirality α-helices with only natural amino acids in AMBP bp69 containing seven l- and four d-residues and in the corresponding mixed chirality linear peptide ln69, both of which are almost perfectly superimposable with the homochiral α-helices of bp65 and ln65.

## Results and discussion

### Optimizing a bicyclic antimicrobial peptide to a mixed chirality analog which retains activity as linear AMP

The previously identified AMBP bp3 featured a particularly short sequence of only 10 residues, but was only active against the Gram-positive *Bacillus subtilis*, a bacterium which is particularly sensitive to membrane disruptive compounds.^42^ An initial chemical space nearest neighbor search using molecular fingerprint similarity as described previously,^41,42^ followed by synthesis and testing of eight close analogs of bp3, indicated that a second leucine residue at the C-terminus to form AMBP bp65 increased the activity spectrum to *Pseudomonas aeruginosa* PAO1, our standard Gram-negative test strain, however at the cost of stronger hemolysis (bp59–bp66, Table S1, ESI† and Table 1).

**Table 1:** Synthesis and activity of bicyclic and linear AMPs

No.	Sequence^a^	SPPS yield^b^ mg (%)	MS analysis calc./obs.^c^	MIC PAO1^d^ (μg mL^−1^)	Hemolysis on hRBC, MHC^e^ (μg mL^−1^)	EYPG vesicle leakage^f^ (%)	EYPC vesicle leakage^f^ (%)
bp3	B^12^KKLLKC^1^ LKC^2^L	21.6 (15)	1330.81/1330.43	>64	500	—	—
bp65	B^12^KKLLKC^1^LKC^2^LL	41.5 (26)	1443.89/1443.90	8	16.6	34	37
bp67	B^12^kKlLkC^1^lKc^2^Ll	51.2 (33)	1443.89/1443.89	32	>2000	48	6
bp68	B^12^KKLLKc^1^LKc^2^LL	55.7 (36)	1443.89/1443.79	16	16.6	44	18
bp69	B^12^kkLLkC^1^LkC^2^LL	45.7 (29)	1443.89/1443.83	16	16.6	48	57
ln65	KKLLKLLKLLL	51.2 (33)	1320.99/1320.99	2–4	125	77	54
ln65b	TolKKLLKC_m_LKC_m_LL	84.0 (58)	1446.92/1446.92	4–8	16.6	40	49
ln69	kkLLkLLkLLL	45.7 (29)	1320.99/1320.99	8	1000	92	7
ln69b	TolkkLLkC_m_LkC_m_LL	102.6 (71)	1446.92/1446.92	16	1000	47	6

a One letter codes for amino acids, B = 3,5-bis(methylene)toluoyl, Tol = toluoyl group, C_m_ = *S*-methyl cysteine. Line notation for bicyclic structures uses single letter codes for amino acids and the SMILES convention for cyclization points.^49,50^

b Yields given for RP-HPLC purified products.

c High-resolution electrospray ionization mass spectrometry (positive mode), the calculated monoisotopic mass, and the observed mass in the reconstructed spectrum are given.

d Minimum inhibitory concentration (MIC) was determined on *P. aeruginosa* PAO1 after incubation for 16–20 h at 37 °C. Values represent two independent triplicate MIC determinations.

e Minimum hemolytic concentration (MHC) measured on human red blood cells in 10 mM phosphate buffer, 150 mM NaCl, pH 7.4, 25 °C.

f Lipid vesicles made of EYPG or EYPC were suspended in buffer (10 mM TRIS, 107 mM NaCl, pH 7.4). After 45 s, the indicated compound was added to reach the indicated concentration. After 285 s, 30 μL of Triton X-100 1.2% was added for full fluorescein release. The percentage leakage observed with the 10 μg mL^−1^ compound at 250 s is given. See Fig. S1 (ESI) for full curves.

To further optimize our AMBP, we prepared several diastereomers of bp65. While alternating l- and d-residues (bp67) produced an AMBP with both reduced hemolysis and reduced antibacterial activity, inverting only cysteines (bp68) or only lysines (bp69) preserved activity and hemolysis. To check whether cyclization was required for activity, we additionally prepared ln65 and ln69 as the linearized analogs of bp65 and bp69 by removing the N-terminal toluoyl staple and replacing both cysteines with leucines, as well as the corresponding ln65b and ln69b bearing an N-terminal toluoyl group and a pair of *S*-methyl cysteines corresponding more precisely to the composition of the bicyclic peptides. All four linear peptides showed antibacterial activities comparable to the parent bicyclic peptides, showing that cyclization was not required for activity (Table 1). Interestingly the two mixed chirality linear analogs ln69 and ln69b additionally lost their hemolytic activity, an effect also sometimes observed with mixed chirality lysine/leucine containing AMPs.^28^

Vesicle leakage assays^51^ showed that all peptides had substantial activity on anionic egg yolk phosphatidyl glycerol (EYPG) vesicles mimicking bacterial membranes. Furthermore, all compounds with hemolytic activity also induced leakage of zwitterionic egg yolk phosphatidyl choline (EYPC) vesicles mimicking eukaryotic membranes, the non-hemolytic peptides bp67, ln69 and ln69b being the only compounds with very low leakage activity on these vesicles. These data suggested that our AMBP and their linear analogs acted by membrane disruption (Table 1 and Fig. S1, ESI†).

A closer characterization of AMBP bp65 and bp69 and their linear analogs ln65, ln65b, ln69 and ln69b showed that these compounds had substantial activity against an extended panel of bacteria including multidrug-resistant strains (Table 2). While AMBPs bp65 and bp69 were both resistant to serum proteolysis as shown previously with other bicyclic peptides,^42,50^ the linearized homochiral analogs ln65 and ln65b were rapidly degraded (Table 2 and Fig. S2, ESI†). The mixed chirality linear AMPs ln69 and ln69b however were entirely stable to serum proteolysis, similar to other mixed chirality AMPs,^27–31^ random peptide mixtures^52,53^ and stereorandomized peptides.^37^

**Table 2:** Extended activity profiling of bicyclic and linear AMPs

Cpd	*P. aeruginosa* ZEM-1A^a^	*P. aeruginosa* ZEM9A^a^	*K. pneumoniae* Oxa-48^a^	*E. coli* W3110^a^	*A. baumannii* BAL225^a^	*S. aureus* Newman^a^	*S. aureus* COL^a^	Serum stability (%)^b^
bp65	8	>32	16	8	4	8	8	93
bp69	4–8	>32	16	8	4	2–4	4	93
ln65	2–4	16	4	4	2–4	2–4	4	0
ln69	8	>32	8	4	2–4	8	16	96
ln65b	4	16	8	8	4	4	4	20
ln69b	8	>32	>32	8	8	32	32	96

a Minimal inhibitory concentration (MIC) in μg mL^−1^ in the Mueller–Hinton (MH) broth after incubation for 16–20 h at 37 °C.

b % of undegraded peptide after incubation in 25% human serum after 24 h. All experiments were performed in two independent triplicates. See Fig. S2 (ESI) for full curves.

### CD spectra indicate α-helical folding in a membrane environment

**Fig. 2:**
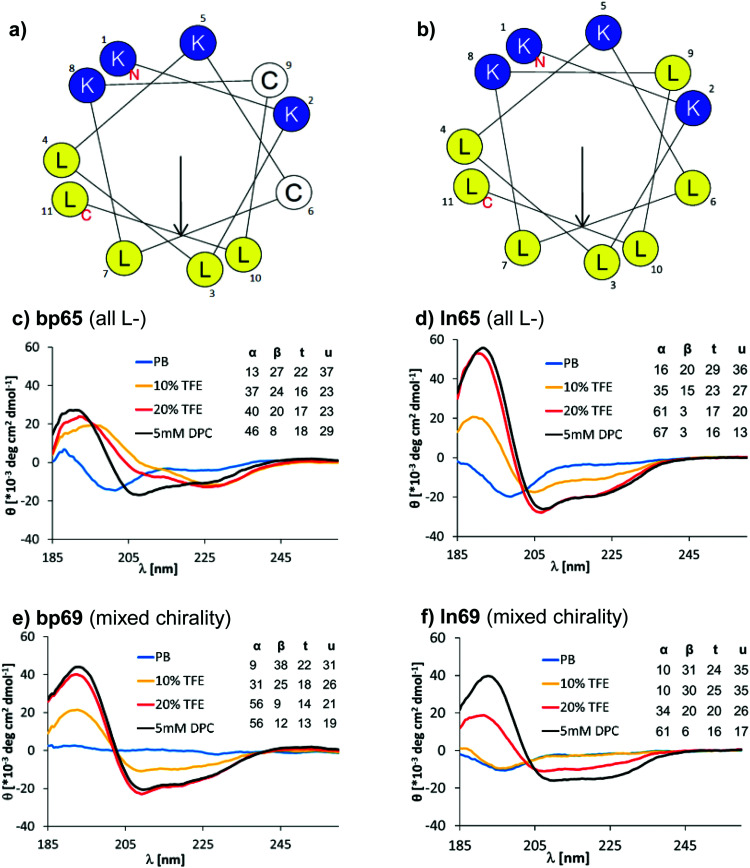
(a) Helix properties of bp65 predicted by HeliQuest.^58^ Blue indicates cationic residues, yellow indicates hydrophobic residues and white indicates cysteines bound to the linker. The arrow inside the helix wheel indicates the magnitude and direction of the hydrophobic moment. (b) Same as (a) for ln65. (c) CD spectrum of bp65 recorded at 0.100 mg mL^−1^ in 7 mM phosphate buffer at pH 7.2 with the addition of 0, 10, 20% TFE or 5 mM DPC. The primary CD spectra were analyzed using Dichroweb, and the percentages of secondary structures were extracted. α = alpha, β = beta, t = turn, u = unordered. The Contin-LL method and reference set 4 were used.^59^ See the ESI† for full CD spectra (Fig. S3). (d) Same as (b) for ln65. (e) Same as (b) for bp69. (f) Same as (b) for ln69.

A helix-wheel analysis of AMBP bp65 showed that its N-terminus and both cysteine residues resided on the same face of a possibly typical AMP-like amphiphilic α-helix with cationic and hydrophobic side chains on opposite sides of the helix (Fig. 2a). Indeed, bp65 showed a CD (circular dichroism) spectrum typical for an α-helical AMP, with a transition from an unordered conformation in water to an α-helical conformation upon addition of up to 20% v/v trifluoroethanol (TFE) or in the presence of 5 mM *n*-dodecylphosphocholine (DPC) micelles mimicking the membrane environment (Fig. 2c). Its linear analogs ln65 and ln65b, which had the same helix-wheel amphiphilic arrangement as bp65 (Fig. 2b), showed similar medium dependent CD spectra (Fig. 2d and Fig. S3, Table S2, ESI†), showing that, in contrast to hydrocarbon and related staples which most often increase α-helicity,^54–57^ our bicyclic staple did not influence α-helical folding.

Surprisingly, the mixed chirality sequences bp69 and ln69 showed similar CD spectra and an even stronger α-helical signal as their homochiral parents in 5 mM DPC although they contained 7 l- and 4 d-residues (Fig. 2e, f and Fig. S3, Table S2, ESI†). These data suggested a membrane-induced helical folding reminiscent of the helical fold deduced on the basis of FTIR for certain mixed chirality AMPs.^32–35^ In this case however, the linear peptide ln69b containing the N-terminal toluoyl group and a pair of *S*-methyl cysteines exactly matching bp69 showed lower α-helicity in 20% TFE than bp69 and ln69, suggesting a slightly lower folding ability (Table S2, ESI†), consistent with its lower antimicrobial and membrane disruptive activity (Tables 1 and 2).

### X-ray crystallography shows α-helices in bicyclic, linear and mixed chirality AMPs

To get a direct insight into the structure of AMBP bp65/bp69 and their linear analogs ln65/ln69, we performed several crystallization studies using either the compounds themselves or their racemates to be crystallized as pure compounds, or their fucosylated derivatives of both mirror image peptides as complexes with the *P. aeruginosa* lectin LecB. From several hundred crystallization attempts under various conditions, we obtained seven well diffracting crystals (Table 3).

**Table 3:** X-Ray crystallography of homochiral and mixed chirality AMBP and AMPs

No.	Sequence^a^	Conditions	PDB code	Res.	*N* b
*rac*-bp65	B^12^KKLLKC^1^LKC^2^LL/B^12^kkllkc^1^lkc^2^ll	10 mg mL^−1^ peptide, 0.1 M sodium citrate pH 5.6, 35% v/v *t*-BuOH (crystal screen F5)	6Y14	0.9 Å	2
bp70	B^12^HONleYDabC^1^IRC^2^YA	10 mg mL^−1^ peptide, 1.6 M (NH_4_)_2_SO_4_, 0.1 M MES pH 6.5, 10% v/v 1,4-dioxane, 2% v/v glycerol (crystal screen F11 + 2% v/v Glycerol)	6Y13	1.1 Å	1
		10 mg mL^−1^ peptide, 0.2 M CaCl_2_, 0.1 M NaOAc pH 4.6, 20% v/v i-PrOH (crystal screen B12)	6Y1S	1.1 Å	1
bp71	B^12^kkLLkC^1^LkC^2^LLK(*)	40 μM LecB, 200 μM peptide, 1.5 M (NH_4_)_2_SO_4_, 0.1 M Tris pH 8.5, 12% v/v glycerol (crystal screen H6)	6Y0U	1.5 Å	3
		40 μM LecB, 200 μM peptide, 0.01 M CoCl_2_, 0.1 M NaOAc pH 4.6, 1.0 M 1,6-hexanediol (crystal screen E11)	6Y0V	1.7 Å	3
Fln65	(*)KKLLKLLKLLL	40 μM LecB, 200 μM peptide, 0.2 M magnesium formate dihydrate (crystal screen D8)	7NEF	1.5 Å	8
Fdln69	(*)KKllKllKlll	40 μM LecB, 200 μM peptide, 0.2 M sodium citrate, 0.1 M sodium cacodylate pH 6.5, 30% v/v i-ProH (crystal screen A8)	7NEW	2.0 Å	2

a One letter codes for amino acids, B = 3,5-bis(methylene)toluoyl, Nle = norleucine, Dab = diaminobutyric acid, * = α-l-fucosyl-acetyl. The line notation for bicyclic structures uses single letter codes for amino acids and the SMILES convention for cyclization points. The structural formulae of all compounds are shown in the ESI.

b *N* = number of crystallographically non-equivalent structures.

**Fig. 3:**
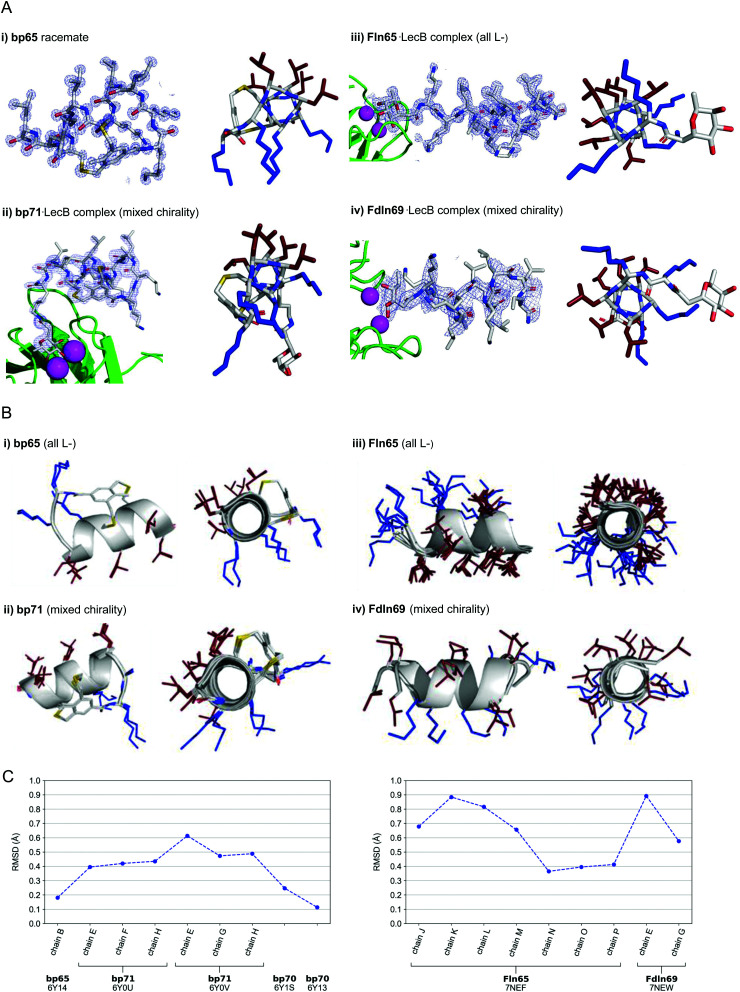
(A) X-Ray crystallography of α-helical homochiral and mixed chirality AMPs and AMBPs. (i) X-Ray crystal structure of bp65. Left panel: Electron density as blue mesh corresponding to bp65 observed in the X-ray crystal structure of the bp65 racemate in PDB 6Y14. Right panel: Stick model of the bp65 crystal structure, lysine side chains shown in blue and leucine side chains shown in brown. See the ESI† for details. (ii) X-Ray crystal structure of the bp71·LecB complex. Left panel: Electron density as blue mesh for bp71 in one of the 4 crystallographically distinct LecB binding sites in PDB 6Y0U. Peptide, Ca^2+^ atoms and LecB monomer are represented as in (ii). Right panel: Stick model of bp71 as observed in the crystal, with full side chains added for clarity, color coded as in (i). See the ESI† for details. (iii) X-Ray crystal structure of the Fln65·LecB complex. Left panel: Electron density (blue mesh) for Fln65 in one of the eight crystallographically distinct LecB binding sites in PDB 7NEF. The peptide is represented with sticks, Ca^2+^ atoms of the lectin binding site are shown with magenta spheres and the LecB monomer with green cartoon. Right panel: Stick model of Fln65 as observed in the crystal structure, color coded as in (i). See the ESI† for details. (iv) X-Ray structure of the Fdln69·LecB complex. Left panel: Electron density (blue mesh) for Fdln69 in one of the four crystallographically distinct LecB binding sites in PDB 7NEW. Peptide, Ca^2+^ atoms and the LecB monomer are represented as in (ii). Right panel: Stick model of Fdln69 as observed in the crystal structure, color coded as in (i). See the ESI† for details. (B) Superpositions of non-equivalent peptides within the X-ray structures. (i) bp65: Superposition of the 2 non-equivalent l-peptides in the unit cell of PDB 6Y14. (ii) bp71: Superposition of the 6 most complete peptides of PDB entries 6Y0U and 6Y0V. (iii) Fln65: Superposition of the 8 non-equivalent peptides of PDB 7NEF. (iv) Fdln69: superposition of the 2 non-equivalent of PDB 7NEW. Peptides are shown as grey cartoon with the side chains as sticks. Amino acid side chains are color-coded: brown = hydrophobic, blue = cationic. (C) Left panel: Root-mean-square deviation (RMSD) calculated with PyMol between the backbone of bp65 (6Y14, chain A) and the backbone of every non-equivalent bicyclic peptides obtained by X-ray crystallography: bp65 (6Y14, chain B), bp71 (6Y0U, chains E, F and H; 6Y0V, chains E, G and H) and bp70 (6Y1S and 6Y13). Right panel: RMSD calculated with PyMol between the backbone of Fln65 (7NEF, chain I) and the backbone of every non-equivalent linear peptide obtained by X-Ray crystallography of Fln65 (7NEF, chains J, K, L, M, N, O and P) and Fdln69 (7NEW, chains E and G). Enantiomers of both Fdln69 chains were used for these calculations. All fucosyl groups were removed for superpositions and RMSD calculations.

In the crystal structure of the homochiral AMBP bp65 obtained as a racemic mixture with its d-enantiomer, the unit cell contained two non-equivalent peptides, each in l- and d-forms related by an inversion center (6Y14, 0.9 Å resolution, Table S3.1 and Fig. S4.1, ESI†). For the two structures obtained for bp70, an analog of bp65 with multiple conservative exchanges of leucines and lysines, the unit cells were occupied by 6, respectively 24, symmetrically related copies of a single peptide (PDB 6Y13, 1.1 Å resolution, Table S3.2 and Fig. S4.2, and PDB 6Y1S, 1.1 Å resolution, Table S3.3 and Fig. S4.3, ESI†). In all four individually resolved homochiral bicyclic structures, the peptides were present in almost perfectly superimposable conformations featuring nine of the eleven residues forming two full turns of a right-handed α-helix including the pair of stapling cysteines (Fig. 3A and B and Fig. S4.4, ESI†). The two N-terminal residues and the N-terminal toluoyl double staple formed a turn conformation. In all four cases the α-helical fold resulted in an amphiphilic arrangement of hydrophobic and cationic residues corresponding to the helix-wheel model and explaining the membrane disruptive activities discussed above (Fig. 3B).

For the mixed chirality AMBP bp69, we obtained two crystal structures of lectin LecB complexed with bp71, an analog of the mixed chirality AMBP bp69 with an additional side-chain fucosylated lysine at the C-terminus. In these two very similar X-ray structures, three of the four non-equivalent fucose binding pockets were occupied by moderately to well resolved peptides in an α-helical conformation (PDB 6Y0U, 1.5 Å resolution, Table S3.4 and Fig. S4.5; and PDB 6Y0V, 1.7 Å resolution, Table S3.5 and Fig. S4.6, ESI†).^44,46–48^ The remaining binding pocket showed electron densities only for the anchoring fucose, indicating a disordered conformation. The six individual structures of the mixed chirality AMBP featured essentially the same double-stapled amphiphilic α-helix as that observed in the parent homochiral AMBP structures (Fig. 3A and B).

In the case of the linear AMPs, we obtained X-ray crystal structures of the homochiral AMP ln65 in the form of LecB complexes with the N-terminally fucosylated analog Fln65 (PDB 7NEF, 1.51 Å resolution, Table S3.6 and Fig. S4.7, ESI†). The LecB complex with the homochiral sequence Fln65 showed eight crystallographically distinct structures, which represented amphiphilic α-helices (Fig. 3A and B). Similarly, we obtained an X-ray crystal structure of the mixed chirality linear AMP ln69 as a LecB complex of its N-terminally fucosylated enantiomer Fdln69 (PDB 7NEW, 2.02 Å resolution, Table S3.7 and Fig. S4.8, ESI†). This structure contained four crystallographically non-equivalent fucose binding sites, two of which featured the peptide in a fully formed α-helix (Fig. 3A and B). The other pair of fucose binding sites only showed electron density for the anchoring fucosyl group and the first residue, indicating a disordered conformation, a situation also observed previously with other homochiral peptides and probably caused by crystal packing (Fig. S4.8, ESI†).^47^

Remarkably, the ten crystallographically non-equivalent stapled bicyclic α-helices observed in the X-ray structures of the homochiral *rac*-bp65 and bp70, and the mixed chirality bp71·LecB complex were essentially superimposable, with RMSD < 0.6 Å for the peptide backbones (Fig. 3C, left panel). The same was true across the ten different X-ray structures of linear α-helices observed in the X-ray structures of LecB complexes for the homochiral Fln65 and its mixed chirality analog Fdln69 (RMSD < 0.9 Å, Fig. 3C, right panel). This comparison showed that the helical geometries of the homochiral and heterochiral helices were not significantly different from each other.

### Molecular dynamics simulations confirm the stability of homochiral and heterochiral α-helices in micelles or as helix bundles

While CD spectra indicated that in solution the different peptides were unordered in water and only folded in the presence of DPC micelles or with TFE mimicking the membrane environment, the X-ray structures presented above featured α-helices within the crystal environment in the absence of any membrane lipids. To better understand in which context the folded conformations were stable, we performed MD simulations using GROMACS.^60^

**Fig. 4:**
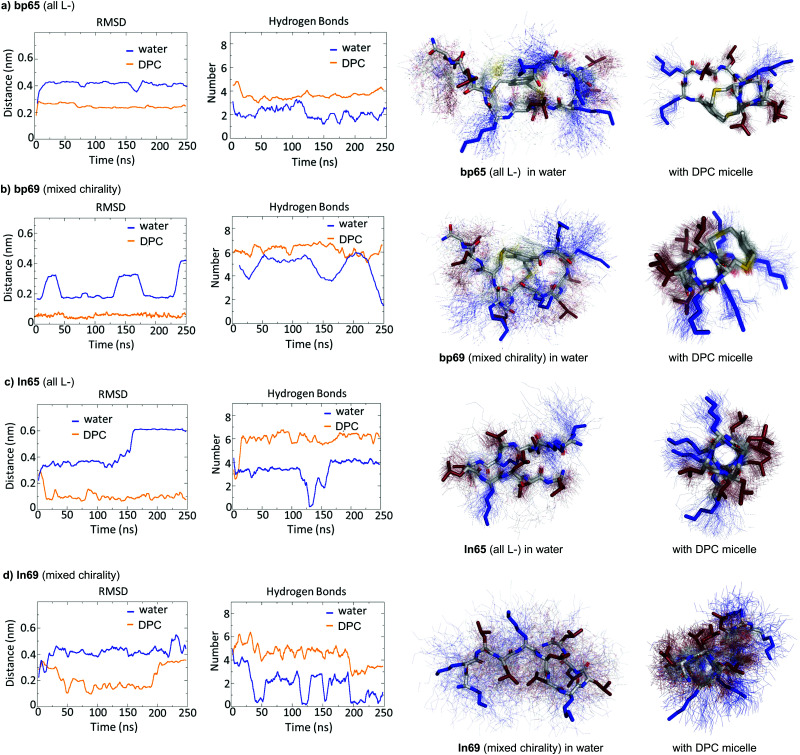
MD simulations in water and in the presence of a DPC micelle. (a) MD simulation of bp65 in water with/without a DPC micelle over 250 ns using GROMACS. Left panel: Root-mean-square deviation of the peptide backbone atoms relative to starting coordinates observed in the crystal structure of bp65. Center left panel: Number of intramolecular backbone hydrogen bonds. Center right and right panels: Average structure (stick model) in water (center right) or with DPC micelle (right panel) over 100 structures sampled over the last 100 ns (thin lines). (b) Same as (a) for bp69, starting from the α-helical coordinates observed in the crystal structure of the LecB·bp71 complex. (c) Same as (a) for ln65 starting from the α-helix model built in PyMol. (d) Same as (a) for ln69 starting from the α-helix model built in PyMol. The DPC micelle was omitted for clarity.

For the homochiral AMBP bp65, MD simulation in pure water resulted in rapid unfolding of the α-helix and formation of a disordered conformation. In the presence of a DPC micelle by contrast, the AMBP rapidly adsorbed to the micelle surface and refolded into an α-helix, reproducing the CD observation in the presence of micelles (Fig. 4a). MD simulations with the mixed chirality AMBP bp69 gave similar results (Fig. 4b). To test whether the α-helix was stabilized by the bicyclic staple, we repeated the MD simulation with linear analogs ln65 and ln69, which again showed rapid unfolding in water but refolding to a stable α-helix at the DPC micelle surface for both the homochiral linear peptide ln65 and the mixed chirality linear peptide ln69 (Fig. 4c and d). These simulations confirmed the similar folding behavior of homochiral and mixed chirality bicyclic or linear peptides, both requiring a membrane-like environment to form, and provided further evidence that the bicyclic staple did not contribute to helix stability.

**Fig. 5:**
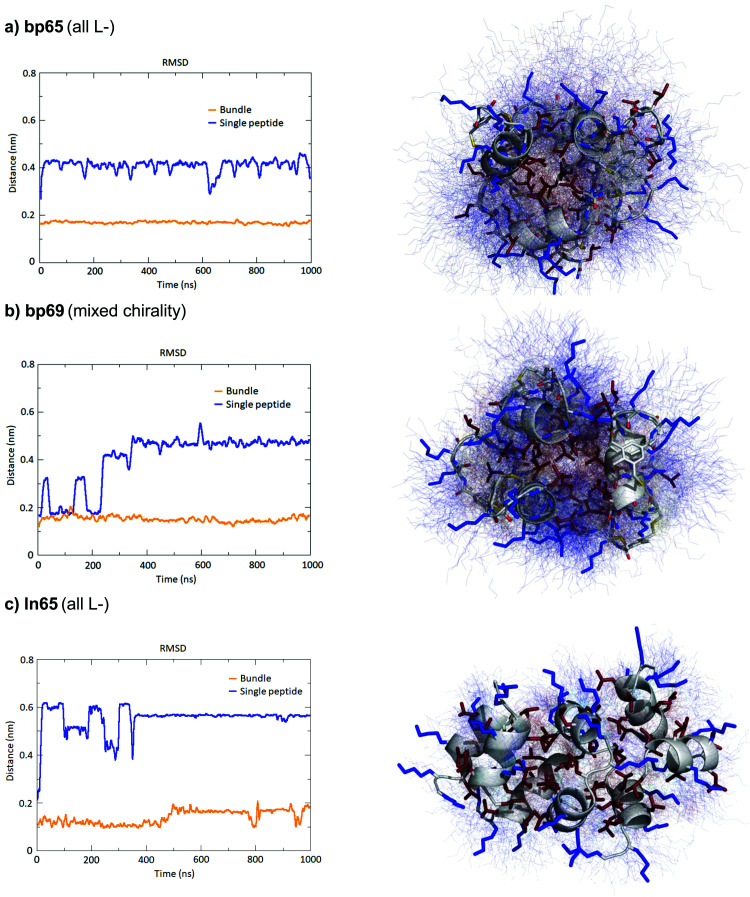
MD simulations of helix bundles for bp65, bp69 and ln65 from X-ray structures of bp65, bp71 and Fdln65. (a) MD simulations of bp65 as a bundle. Left panel: Root mean square distances of the backbone atoms relative to the backbone atoms of the starting model of a single peptide in the bundle compared to single peptides in water. Right panel: Overlay of 100 states over the 1000 ns run trajectory of the bundle in water. The average structure is shown as cartoon (backbone) and sticks (sidechains) and the 100 states as thin lines. Hydrophobic side chains are colored in red and cationic side chains in blue. (b) Same as (a) for bp69 for the bundle containing three complete peptides in the bundle out of four. (c) Same as (a) for ln65. All fucoses were removed before simulations.

The MD simulations in water and DPC micelles were consistent with the CD observations but did not explain why α-helices could be observed in the X-ray crystal structures where the peptides are surrounded only by a largely aqueous solvent. A closer analysis of the different X-ray structures showed that the peptides did not make any significant contact with LecB apart from the anchoring fucose residue. In the case of bp65, bp71 and Fln65 the peptides were aggregated in bundles of four of more helices *via* hydrophobic contacts between leucine side chains. MD simulations showed that these helix bundles were stable in water and retained the α-helical fold of the individual peptides thanks to stabilizing hydrophobic contacts in the absence of a membrane environment as observed previously with related AMPs (Fig. 5 and Fig. S5, ESI†).^47^

## Conclusions

By investigating the structure of AMPBs we discovered the mixed chirality sequence bp69 containing seven l- and four d-residues folding into an amphiphilic α-helix within a stapled bicyclic peptide similar to the parent all l-AMBP bp65. A similar α-helical fold was observed for the corresponding linear mixed chirality peptide ln69 and the homochiral sequence ln65. The mixed chirality linear AMP ln69 showed comparably strong antibacterial activities but reduced hemolysis and much stronger stability against serum proteolysis compared to its homochiral parent ln65, similar to previous reports with mixed chirality AMPs.^27–35^ The homochiral or heterochiral bicyclic and linear α-helices were observed by CD in a micellar environment as well as by X-ray crystallography of peptides or their complexes with LecB. MD simulations confirmed that the mixed chirality formed comparably well to the homochiral helices in micelles or as helix bundles in water as observed by X-ray crystallography.

While previous reports of similar mixed chirality AMPs concluded on either unordered conformations or helical conformations appearing in a membrane environment based on deconvolution of FTIR spectra,^32–35^ here we reported X-ray crystallographic evidence of short mixed chirality helices consisting of only natural residues existing as helix bundles within the protein environment of a lectin and almost perfectly superimposable with their parent homochiral α-helices. These direct observations unequivocally demonstrate that the α-helical fold with only natural residues can sometimes tolerate multiple stereochemical inversions without significant conformational changes. Together with the fact that mixed chirality sequences appear to be entirely stable in serum, our observation suggests a vast unexplored territory of α-helical mixed chirality sequences as stabilized replacements for all l-bioactive α-helical peptides.

## Methods

Synthesis and characterization of peptides and all assays, measurements, and modeling studies are described in the ESI.† No unexpected or unusually high safety hazards were encountered.

## Author contributions

SB and HP designed and carried out structural and functional studies and modeling simulations and wrote the paper. IDB designed the study, and synthesized and characterized bicyclic peptides. TK and CvD designed and supervised experiments with MDR bacteria. AS supervised and carried out X-ray crystallography. SJ supervised molecular dynamics studies and wrote the paper. JLR designed and supervised the study and wrote the paper. All authors read and commented on the paper.

## Conflicts of interest

The authors declare no competing financial interest.

## Supplementary Material

CB-002-D1CB00124H-s001
