# Supplementary material for: A mixed chirality α-helix in a stapled bicyclic and a linear antimicrobial peptide revealed by X-ray crystallography
Source: RSC Chem Biol. 2021 Aug 20;2(6):1608–17. doi: 10.1039/d1cb00124h (PMC8637766; doi:10.1039/d1cb00124h)
Supplement: CB-002-D1CB00124H-s001 [file CB-002-D1CB00124H-s001.pdf]

Electronic Supplementary Material (ESI) for RSC Chemical Biology.
This journal is © The Royal Society of Chemistry 2021

Supporting Information

Mixed chirality α-helix in a stapled bicyclic and a linear antimicrobial peptide revealed by X-ray crystallography

Stéphane Baeriswyl,[¥a)] Hippolyte Personne,[¥a)] Ivan Di Bonaventura,[a)] Thilo Köhler,[b)] Christian van Delden,[b)] Achim Stocker,[a)] Sacha Javor[a)] and Jean-Louis Reymond [a)*]

[a)] *Department of Chemistry, Biochemistry and Pharmaceutical Sciences, University of Bern, Freiestrasse 3, 3012 Bern, Switzerland;* [b)] *Department of Microbiology and Molecular Medicine, University of Geneva, Service of Infectious Diseases, University Hospital of Geneva, Geneva, Switzerland*

e-mail: jean-louis.reymond@dcb.unibe.ch

[¥] equal contributions

Table of contents

1 PEPTIDE SYNTHESIS 3

1.1 Materials and Methods 3

1.2 Synthesis of Bicyclic peptides 4

1.3 Synthesis of Fucosylated Bicyclic Peptides for Crystallization Studies 5

1.4 Synthesis of linear peptides 6

1.5 Synthesis of Fucosylated linear peptides for crystallization studies 7

2 ANTIMICROBIAL ACTIVITY 9

3 HEMOLYSIS ASSAY 10

4 LIPID VESICLE LEAKAGE ASSAYS 10

5 SERUM STABILITY ASSAY 14

6 CIRCULAR DICHROISM SPECTROSCOPY 15

7 CRYSTALLOGRAPHY 18

8 MOLECULAR DYNAMICS 34

8.1 Parameters for the non-natural residues 35

8.2 MD in the presence of a DPC micelle 37

8.3 Topology for the DPC molecule[13] 37

8.4 Clustering of stable structures 39

9 HPLC AND MS DATA 40

10 REFERENCES 63

1 Peptide synthesis

1.1 Materials and Methods

SPPS – oxyma pure (hydroxyiminocyanoacetic acid ethyl ester) was purchased from SENN AG, *N*,*N*'-diisopropyl carbodiimide (DIC) was purchased from Iris Biotech GMBH, *N*-ethyldiisopropylamine potassium iodide, piperidine, triisopropylsilane, 2,2'-(ethylenedioxy) diethanethiol were purchased from Sigma Aldrich, phenylsilane was purchased from TCI (Tokyo Chemical Company), 3,5-bis(chloromethyl)-4-methylbenzoic acid was purchased from Enamine, trifluoroacetic acid (TFA) purchased by Fluorochem Ltd, chemicals were used as supplied and solvents were of analytical grade. Amino acids were used as the following derivatives: Fmoc-Cys(Trt)-OH, Fmoc-Leu-OH, Fmoc-Lys(Boc)-OH, Fmoc-His(Boc)-OH, Fmoc-Orn-OH, Fmoc-Tyr(*t*Bu)-OH, Fmoc-Ile-OH, Fmoc-Arg(Pbf)-OH , Fmoc-Nle-OH, and Fmoc-Ala-OH. Tentagel S RAM resin was purchased from Rapp Polymere. Cyclic peptide synthesis was performed automatically by Liberty Blue Automated Microwave Peptide Synthesizer.

LC and MS – Analytical RP-HPLC was performed with an Ultimate 3000 Rapid Separation LC-MS System (DAD-3000RS diode array detector) using an Acclaim RSLC 120 C18 column (2.2 µm, 120 Å, 3×50 mm, flow 1.2 mL/min) from Dionex. Data recording and processing was done with Dionex Chromeleon Management System Version 6.80 (analytical RP-HPLC). All RP-HPLC were using HPLC-grade acetonitrile and Milli-Q deionized water. The elution solutions were: A MilliQ deionized water containing 0.1% TFA; B MilliQ deionized water/acetonitrile (50:50, v/v); D MilliQ deionized water/acetonitrile (10:90, v/v) containing 0.1% TFA. Preparative RP-HPLC was performed with a Waters automatic Prep LC Controller System containing the four following modules: Waters2489 UV/Vis detector, Waters2545 pump, Waters Fraction Collector III and Waters 2707 Autosampler. A Dr. Maisch GmbH Reprospher column (C18-DE, 100×30 mm, particle size 5 µm, pore size 100 Å, flow rate 40 mL/min) was used. Compounds were detected by UV absorption at 214 nm using a Waters 248 Tunable Absorbance Detector. Data recording and processing was performed with Waters ChromScope version 1.40 from Waters Corporation. All RP-HPLC were using HPLC-grade acetonitrile and Milli-Q deionized water. The elution solutions were: A MilliQ deionized water containing 0.1% TFA; D MilliQ deionized water/acetonitrile (10:90, v/v) containing 0.1% TFA. MS spectra, recorded on a Thermo Scientific LTQ OrbitrapXL, were provided by the MS analytical service of the Department of Chemistry and Biochemistry at the University of Bern (group PD Dr. Stefan Schürch). Yields are given as total yields (SPPS and second thioether ligation).

1.2 Synthesis of Bicyclic peptides

Linear peptides were synthesized by CEM Liberty Blue Microwave peptide synthesizer. The synthesis was carried out by using Rink Amide MBHA resin (100-200 mesh), unloaded (0.78 mmol/g), 0.25 scale (150 mg of resin). The resin was swollen in DMF/DCM 50:50 for 15 min. at R.T.

Removal of the Fmoc protecting group – At each step the Fmoc protecting group was removed with 3 mL of piperidine/DMF (1:4, v/v) for 2 min at 75 °C. After filtration the resin was washed 3 times for 7 sec. with DMF.

Coupling of the Fmoc-protected amino acids – 5 eq. of Fmoc-protected amino acid with a concentration of 0.2 M, 5 eq. of Oxyma and 6 eq. of DIC, both with a concentration of 0.2 M, were used as coupling reagents in 4.5 mL of DMF. The reaction was stirred for 5 min. at 75 °C or 50°C with cysteines. The resin was then washed with DMF for 7 sec. for 3 times.

3,5-Bis(chloromethyl)-4-methylbenzoic acid was coupled by CEM – 5 eq. of the linker, 5 eq. of Oxyma and 6 eq. of DIC, both with a concentration of 0.2 M, were used as coupling reagents in 4.5 mL of DMF. The reaction was stirred for 5 min. at 75 °C and repeated twice. The resin was then washed with DMF for 7 sec. for 3 times.

Cleavage of the linear peptides – The cleavage was carried out by treating the resins with 7 mL of a TFA/DODT/TIS/H_2O (94:2.5:2.5:1, v/v/v/v) solution for 3 h. The peptide solutions were precipitated with 25 mL of TBME, centrifuged for 10 min at 3500 rpm (twice), evaporated and dried in high vacuum for 60 min.

Peptide bicyclization by double thioether ligation was performed in solution. The crude dried linear peptides were dissolved in 40 mL ACN/H_2O (50:50, v/v) in order to be diluted enough to reduce dimer formation. The solution was basified to pH 8 with DIPEA and 5eq KI were added. The reaction was run under vigorous stirring and completion was checked by LC-MS after 30 min and the solution was lyophilized directly upon completion.

Purification of the bicyclic peptides – The dried crude was dissolved in a water/ACN mixture, filtered (pore size 0.22μm) and purified by preparative RP-HPLC with gradients of 60 min. Fractions were analysed by LC-MS with a 5 min gradient. Peptides were obtained as white foamy solids after lyophilization. Yields were calculated for the TFA salts.

1.3 Synthesis of Fucosylated Bicyclic Peptides for Crystallization Studies

Linear peptides were synthesized by CEM Liberty Blue Microwave peptide synthesizer as shown in 1.2. For sequences designed to be functionalized with the Peracetylated α-L-fucosyl-acetic acid, Tentagel S RAM resin (unloaded, 0.23 mmol/g) was preferred for its ability to swell in aqueous conditions for the deacetylation step. The sequences included Fmoc-Lys(Alloc)-OH in the peptide sequence on the C-terminus.

Alloc deprotection – The polypropylene syringe was equipped with a septum and dried under vacuum for one hour. It was then swollen in dry DCM for 20 min under argon. After removal of the solvent, $Pd(PPh_3)_4$ (0.25 eq.) was diluted in 8 mL of dry DCM and added to the resin under argon. Phenylsilane (25 eq.) was then added to the resin. The reaction was stirred under argon bubbling for 45 min. (2×). The reagents were then removed by filtration and the resin washed with dry DCM (6 mL, 2×15 min) and sodium diethylamino dithiocarboxylate (20 mM in DMF, 6 mL, 10 min).

Coupling of the sugar – Peracetylated α-L-fucosyl-acetic acid [1] (5 eq.), HATU (4 eq.) and DIPEA (10 eq.) were dissolved in 4.5 mL of NMP and 1.5 mL of DCM and added to the syringe. The mixture was stirred overnight. The solvent was filtrated and the washing step, NMP (2×6 mL), MeOH (2×6 mL) and DCM (2×6 mL), was performed.

Fmoc deprotection was performed as stated above and 3,5-bis(chloromethyl)-4-methylbenzoic acid was coupled following the procedure previously described. Cleavage, purification of the linear peptide, bicyclization in solution were performed as stated above in 1.2.

Sugar deacetylation in solution – Once the lyophilization was completed, the bicyclic glycopeptide was dissolved and stirred 24 h in a mixture of $MeOH/H_2O/NH_3$ (8:1:1). The MeOH was removed by reduced pressure and the solution was lyophilized. Finally, the purification of the bicyclic compound was performed using RP-HPLC as shown above.

1.4 Synthesis of linear peptides

Linear peptides were synthesized either manually or with Biotage® Initiator+ AlstraTM automated synthesizer. The synthesis was carried out by using Fmoc- Tentagel S RAM resin, unloaded (0.23 mmol/g), 0.1 mmol scale. The resin was swollen in DMF/DCM 50:50 for 15 min. at R.T.

Removal of the Fmoc protecting group – At each step the Fmoc protecting group was removed with 3 mL of piperidine/DMF (1:4, v/v) for 2 min at 75 °C. After filtration the resin was washed 3 times for 7 sec. with DMF.

Coupling of the Fmoc-protected amino acids – The resin was swelled at 50°C (25 W microwave) in DMF for 20 min. Fmoc deprotection was performed for 3 min at RT and 5 min at 50°C (100 W). The coupling reaction was done in microwave at 50°C (100 W microwave power, 600 s). All couplings were performed with the following parameters: 5 eq (relative to resin loading) of Fmoc amino acid and 5 eq (relative to resin loading) Oxyma in DMF and 5 eq (relative to resin loading) DIC.

Cleavage of the linear peptides – The cleavage was carried out by treating the resins with 7 mL of a TFA/DODT/TIS/H_2O (94:2.5:2.5:1, v/v/v/v) solution for 3 h. The peptide solutions were precipitated with 25 mL of TBME, centrifuged for 10 min at 3500 rpm (twice), evaporated and dried in high vacuum for 60 min.

Purification of the linear peptides – The dried crude was dissolved in a water/ACN mixture, filtered (pore size 0.22μm) and purified by preparative RP-HPLC with gradients of 60 min. Fractions were analysed by LC-MS with a 5 min gradient. Peptides were obtained as white foamy solids after lyophilization. Yields were calculated for the TFA salts.

1.5 Synthesis of Fucosylated linear peptides for crystallization studies

Linear peptides were synthesized manually using 250 mg of Tentagel S RAM resin (0.24 mmol/g) and standard Fmoc Solid Phase Peptide Synthesis at 60°C under nitrogen bubbling. The resin was swollen in DMF during 10 min. Double deprotections of Fmoc group were performed using a solution of 5% w/v piperazine / 2% DBU with 10% of butanol in DMF during 1 min then 4 min. The resin was washed 5 times (5 x 8 mL DMF) after deprotection. Double couplings (2 x 8') were performed with 3 mL of amino acid (0.2 M), 2 mL of DIC (0.8 M) and 1.5 mL of Oxyma (0.8 M), all in DMF. Resin was washed twice (2 x 8 mL) between couplings, and 3 times (3x 8 mL DMF) after second coupling.

Coupling of the sugar – Peracetylated α-L-fucosyl-acetic acid [1] (3 eq.), Oxyma (3 eq.) and DIC (3 eq.) were dissolved in 6 mL of NMP. The mixture was stirred overnight. The solvent was filtrated and the washing step, NMP (2×6 mL), MeOH (2×6 mL) and DCM (2×6 mL), was performed.

Sugar deacetylation in solution – Once the lyophilization was completed, the linear glycopeptide was dissolved and stirred 24 h in a mixture of $MeOH/H_2O/NH_3$ (8:1:1). The MeOH was removed by reduced pressure and the solution was lyophilized.

Cleavage of the linear peptides – The cleavage was carried out by treating the resins with 7 mL of a $TFA/TIS/H_2O$ (95:4:1, v/v/v/v) solution for 3 h. The peptide solutions were precipitated with 25 mL of TBME, centrifuged for 10 min at 3500 rpm (twice), evaporated and dried in high vacuum for 60 min.

Purification of the fucosylated linear peptides – The dried crude was dissolved in a water/ACN mixture, filtered (pore size 0.22µm) and purified by preparative RP-HPLC with gradients of 60 min. Fractions were analysed by LC-MS with a 5 min gradient. Peptides were obtained as white foamy solids after lyophilization. Yields were calculated for the TFA salts.

Table S1. Synthesis and activity of the first library of bicyclic **bp3** analogs.

No.	Sequence[a]	SPPS Yield mg (%)[b]	MS analysis calc./obs.[c]	MIC BR 151 µg/mL[d]	MIC PAO1 µg/mL[d]
**bp3**	B^{12}KKLLKC^{1}LKC^{2}L	21.6 (15)	1330.81/1330.43	4	>64
**bp59**	B^{12}KLLKKC^{1}LKC^{2}L	37.4 (26)	1330.81/1330.73	4	>64
**bp60**	B^{12}KLLKLC^{1}KKC^{2}L	31.2 (21.7)	1330.81/1330.70	>64	>64
**bp61**	B^{12}KKLC^{1}KLC^{2}KL	41.2 (31.3)	1217.73/1217.73	>64	>64
**bp62**	B^{12}KKLLLC^{1}KLC^{2}K	33.5 (23.3)	1330.81/1330.81	32	>256
**bp63**	B^{12}KKLC^{1}KLLKC^{2}L	33.5 (23.3)	1330.81/1330.81	4	>64
**bp64**	B^{12}KKLLKC^{1}KLC^{2}L	23.5 (16.3)	1330.81/1330.71	16	>64
**bp65**	B^{12}KKLLKC^{1}LKC^{2}LL	41.5 (26.5)	1443.89/1443.90	2	8
**bp66**	B^{12}KKLC^{1}KLLKC^{2}	41.5 (26.5)	1217.73/1217.71	>64	>64

[a)] One letter codes for amino acids, B = 3,5-bis(methylene)toluoyl. Line notation for bicyclic structures uses single letter codes for amino acids and the SMILES convention for cyclization points.[27,28] [b)] Yields given for RP-HPLC purified product. [c)] Electrospray ionization mass spectrometry (positive mode), the calculated monoisotopic masses, and the observed masses for $[M+H]^+$ are reported. [d)] Minimum inhibitory concentration (MIC) was determined on *B. subtilis* BR151 and *P. aeruginosa* PAO1 after incubation for 16−20 h at 37 °C.

2 Antimicrobial activity

Bicyclic and linear peptides cytotoxicity was assayed against *P. aeruginosa* PAO1, *P. aeruginosa* ZEM-1A, *P. aeruginosa* ZEM9A, *K. pneumoniae* Oxa-48, *E. coli* W3110, *A. baumannii* BAL225, *S. aureus* Newman, *S. aureus* COL (clinical isolate of MRSA), *B. Subtilis* BR151.

To determine the Minimal Inhibitory Concentration (MIC), Broth Microdilution method was used. [2] A colony of bacteria from glycerol stock was grown in LB medium overnight at 37 °C and 180 rpm shaking. The compounds were prepared as stock solutions of 8 mg/mL in sterilized milliQ deionized water, added to the first well of 96-well sterile, polypropylene round bottom microtiter plates (TPP, untreated) and diluted serially by ½. The concentration of the bacteria was quantified by measuring absorbance at 600 nm and diluted to an OD_{600} of 0.022 in MH medium. The sample solutions (150 µL) were mixed with 4 µL diluted bacterial suspension with a final inoculation of about 5×10^5 CFU. For each test, two columns of the plate were kept for sterility control (MH medium only), growth control (MH medium with bacterial inoculum, no compound). The positive control, Polymyxin B (starting with a concentration of 64 µg/mL) in MH medium with bacterial inoculums, was introduced in the two first lines of the plate. The plates were incubated at 37 °C for ~18 hours under static conditions. 15 µL of 3-(4,5-dimethylthiazol-2-yl)-2,5-diphenyltetrazolium bromide (MTT)[3] (1 mg/mL in sterilized milliQ deionized water) were added to each well and the plates were incubated for 20-30 minutes at room temperature. The minimal inhibitory concentration (MIC) was defined as the lowest concentration of the dendrimer that inhibits the visible growth of the tested bacteria (yellow) with the unaided eye.

3 Hemolysis Assay

To determine the minimal hemolytic concentration (MHC) stock solutions of 8 mg/mL of the peptide in H_2O were prepared and 50 µL were diluted serially by l/2 in 50 µL PBS (pH 7.4) in 96-well plate (Costar or Nunc, polystyrene, untreated). Human red blood cells (hRBC) were obtained by centrifugation of 1.5 mL of whole blood, from the blood bank of Bern, at 3000 rpm for 15 minutes at 4 °C. Plasma was discarded and the pellet was re-suspended in a 15 mL Falcon tube in 5 mL of PBS. The washing was repeated three times and the remaining pellet was re-suspended in 10 mL of PBS at a final hRBC concentration of 5%. The hRBC suspension (50 µL) was added to each well and the plate was incubated at room temperature for 4 hours. Minimal hemolytic concentration (MHC) end points were determined by visual determination of the wells after the incubation period. Controls on each plate included a blank medium control (50 µL PBS + 50 µL of hRBC suspension) and a hemolytic activity control (mQ-deionized water 50 µL + 50 µL hRBC suspension).

4 Lipid Vesicle Leakage assays

5(6)-carboxyfluorescein (CF) was purchased from Sigma. Egg Yolk Phosphatidylcholine (EYPC), Egg Yolk Phosphatidylglycerol (EYPG) and a Mini-Extruder were purchased from Avanti Polar Lipids. Egg PC or Egg PG thin lipid layers were prepared by evaporating a solution of 100 mg EYPC or EYPG in 4 mL MeOH/$CHCl_3$ (1:1) on a rotary evaporator at room temperature and then dried in vacuo overnight. The resulting film was then hydrated with 4 mL CF buffer (50 mM CF, 10 mM TRIS, 107 mM NaCl, pH 7.4) for 30 min, subjected to freeze-thaw cycles (7×) and extrusion (15×) through a polycarbonate membrane (pore size 100 nm). Extra vesicular components were removed by gel filtration (Sephadex G-50) with 10 mM TRIS, 107 mM NaCl, pH 7.4 buffer. Final conditions: ~ 2.5 mM PC or PG; inside: 50 mM CF, 10 mM TRIS, 10 mM NaCl, pH 7.4 buffer; outside: 10 mM TRIS, 107 mM NaCl, pH 7.4. PC or PG stock solutions (37.5 µL) were diluted to 3000 µL with a buffer (10 mM TRIS, 107 mM NaCl, pH 7.4) in a thermostated fluorescence cuvette (25 °C) and gently stirred (final lipid concentration ~31 µM). CF efflux was monitored at λ_{em} 517 nm (λ_{ex} 492 nm) as a function of time after addition of the desired volume of peptide (1 or 20 mg/mL stock in mQ water) at t = 45 s. The following final concentrations of peptide were monitored: 5, 10, 15, 20, 25 and 200 µg/mL. Finally, 30 µL of 1.2% Triton X-100 was added to the cuvette (0.012% final concentration) at t = 285 s to reach the maximum intensity. Fluorescence intensities were then

normalized to the maximal emission intensity using $I(t) = (I_t - I_0) / (I_\infty - I_0)$ where $I_0 = I_t$ at peptide addition, $I_\infty = I_t$ at saturation of lysis.

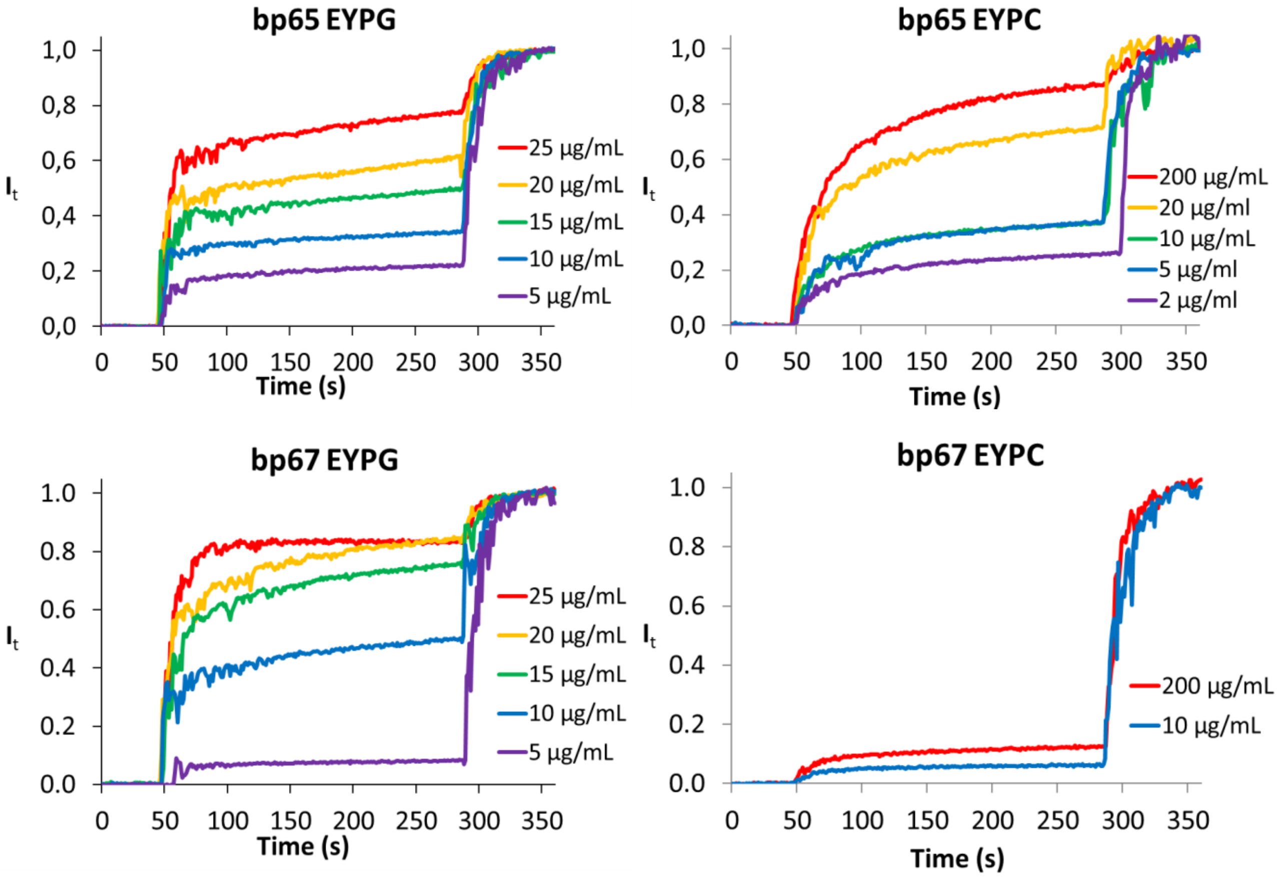

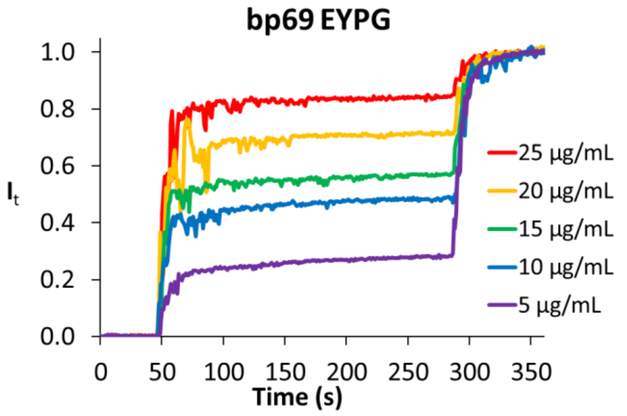
bp69 EYPG
I_t
Time (s)
25 µg/mL
20 µg/mL
15 µg/mL
10 µg/mL
5 µg/mL

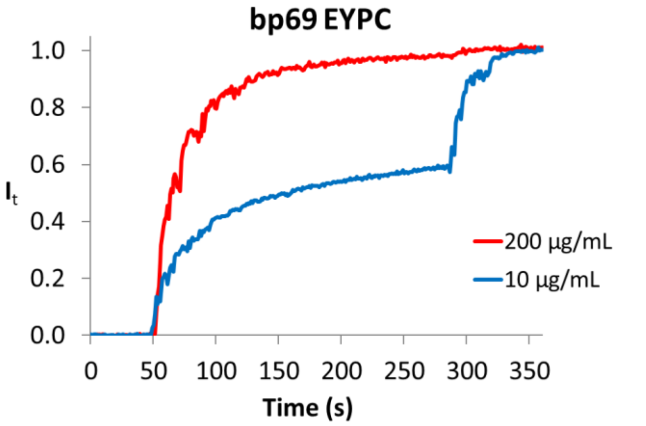
bp69 EYPC
I_t
Time (s)
200 µg/mL
10 µg/mL

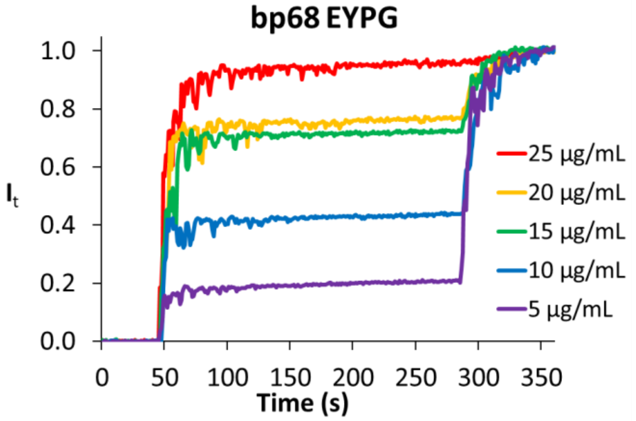
bp68 EYPG
I_t
Time (s)
25 µg/mL
20 µg/mL
15 µg/mL
10 µg/mL
5 µg/mL

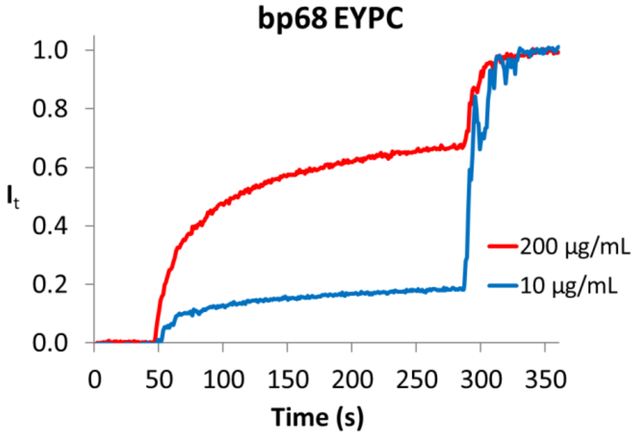
bp68 EYPC
I_t
Time (s)
200 µg/mL
10 µg/mL

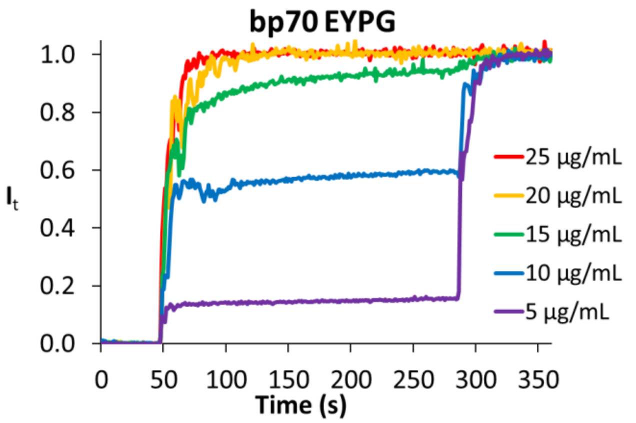
bp70 EYPG
I_t
Time (s)
25 µg/mL
20 µg/mL
15 µg/mL
10 µg/mL
5 µg/mL

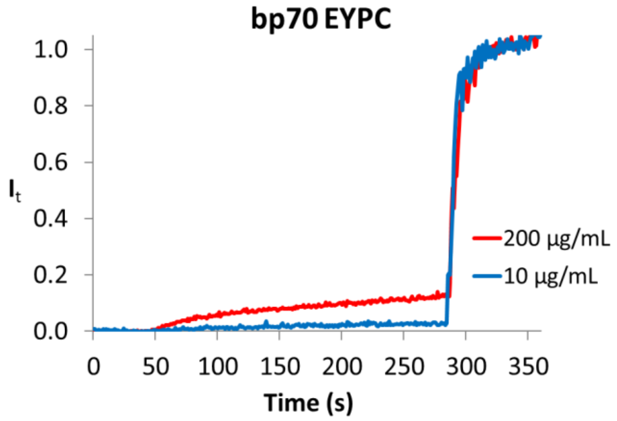
bp70 EYPC
I_t
Time (s)
200 µg/mL
10 µg/mL

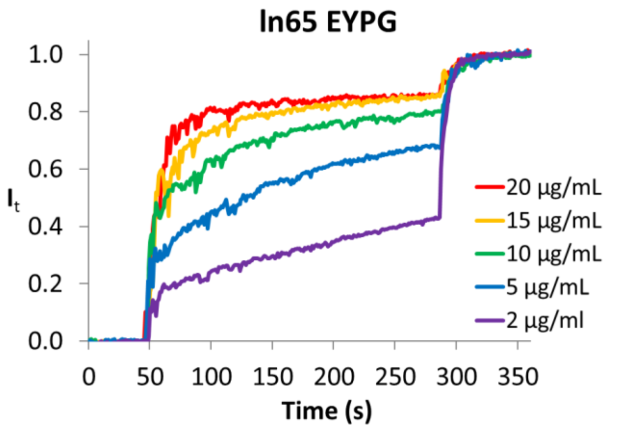
ln65 EYPG
I_t
Time (s)
20 µg/mL
15 µg/mL
10 µg/mL
5 µg/mL
2 µg/ml

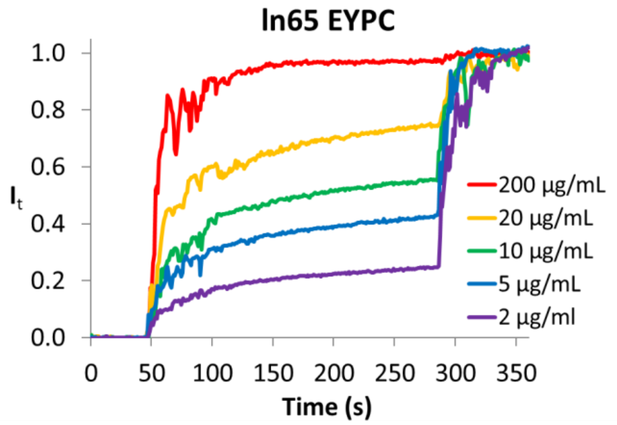
ln65 EYPC
I_t
Time (s)
200 µg/mL
20 µg/mL
10 µg/mL
5 µg/mL
2 µg/ml

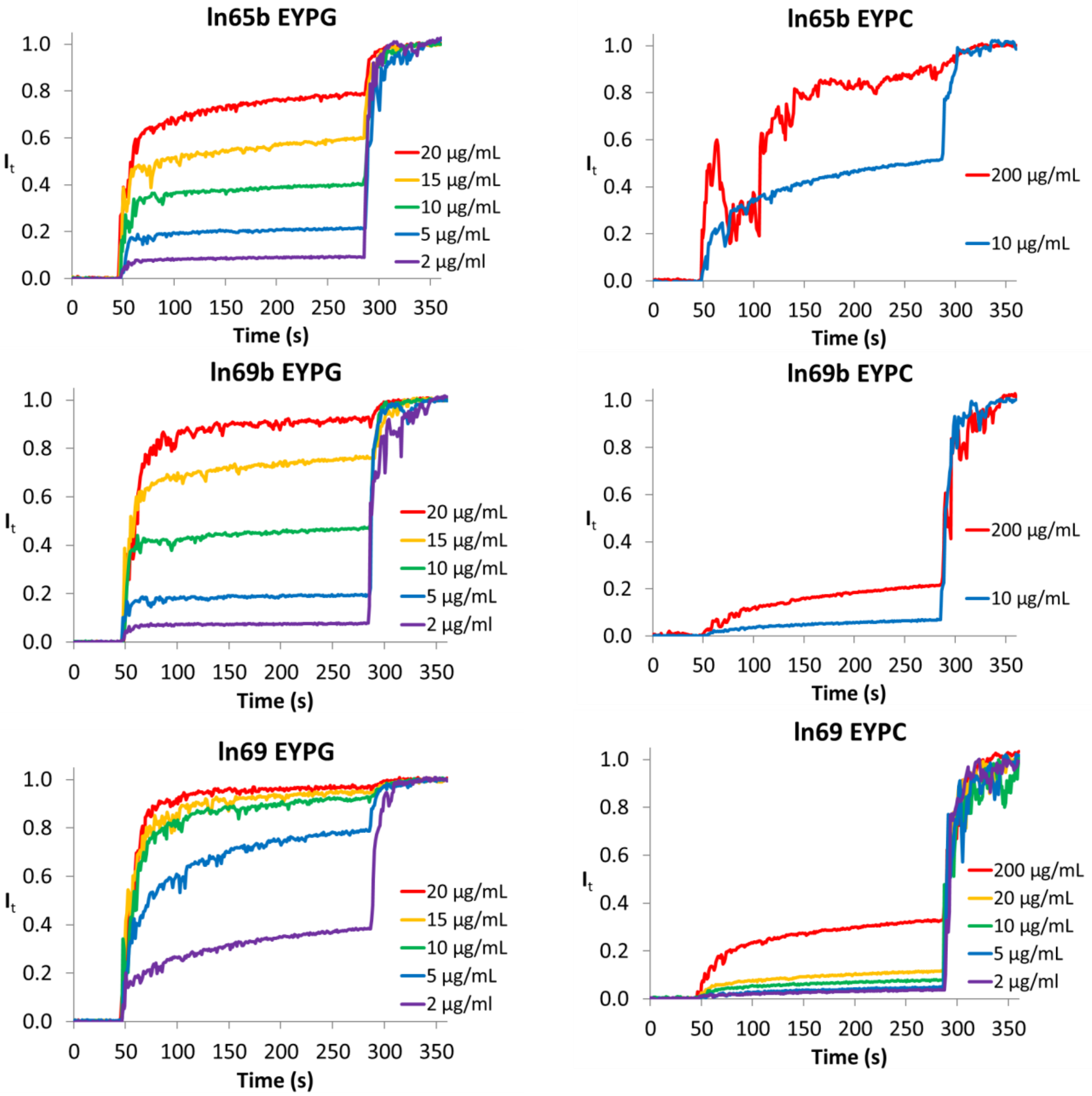

Figure S1: Vesicle leakage experiments using 5(6)-carboxyfluorescein induced by peptides. EYPG and EYPC vesicles were suspended in buffer (10 mM TRIS, 107 mM NaCl, pH 7.4) and the indicated concentration of the compound was added after 45 sec. After 285 seconds 30 μL Triton X-100 1.2% was added for full release of fluorescein.

5 Serum Stability Assay

Human serum was diluted in 0.1 M filtered TRIS buffer pH 7.4 (25%, 1:3, v/v). Selected peptides were diluted in 0.1 M filtered TRIS buffer pH 7.4 to a concentration of 400 µM and 0.1 mg/mL 4-hydroxybenzoic acid was added as internal standard. Aliquots of peptide solution (50 µL) were added to aliquots of serum (50 uL) in sterile Eppendorf tubes, to reach a peptide concentration of 200 µM during the assay. Samples were incubated at 37 °C under gentle stirring (350 rpm). Different samples (triplicates) were quenched at different time points (0/1/6/12/24 h) by precipitating serum proteins through the addition of (0.1 M) $ZnSO_4 \cdot 7\ H_2O$/ ACN (1:1) (0.1 M, 100 µL) and cooling in ice bath for 10 minutes. Protein precipitates were pelleted under centrifugation and the supernatants were sampled and evaporated to dryness in a centrifugal evaporator. Samples were re-suspended in a H_2O/ACN (4:1, v/v) mixture and centrifuged again to remove residual protein precipitate. Supernatants were then sampled and analyzed by LC-MS. Experiment controls included a precipitation control for each peptide, to test their resistance to the protein precipitation conditions, and serum blanks, to check reproducibility over different serum batches. Peaks corresponding to the internal standard and the undegraded peptides were integrated, with the ratio peptide/standard at t = 0 h as 100%.

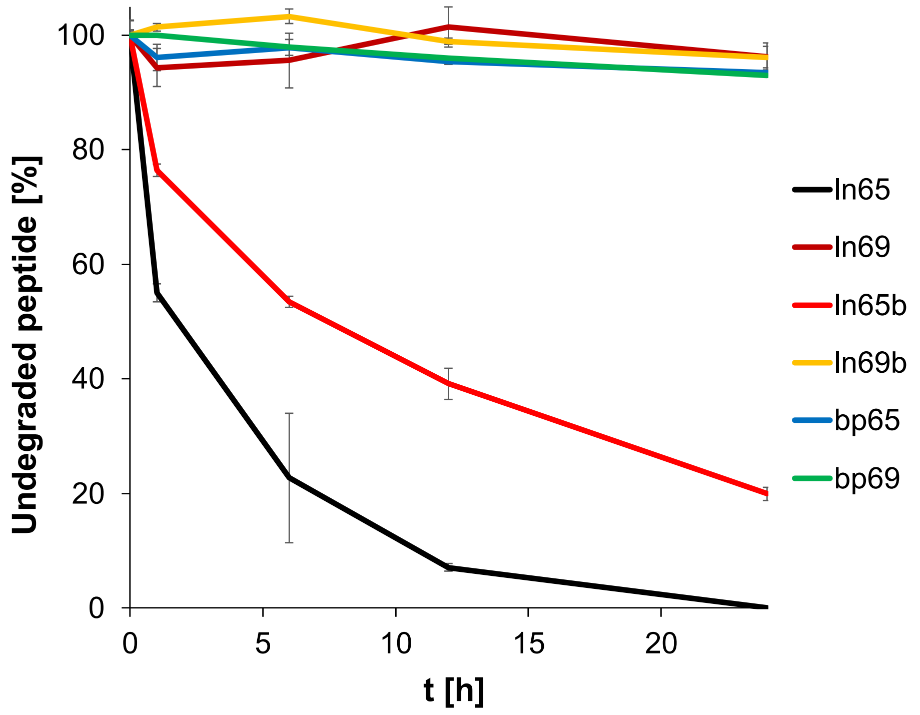

Figure S2: Stability assays of linear peptides (200 µM**)** **ln65**, **ln65b**, **ln69**, **ln69b** and bicyclic peptides **bp65**, **bp69** in 25% human serum in 0.1 M filtered TRIS buffer pH 7.4. Normalized undegraded peptide values determined by RP-HPLC analysis using hydroxybenzoic acid as internal standard. Data from triplicate experiments.

6 Circular dichroism spectroscopy

Circular dichroism (CD) experiments were measured on a Jasco J-715 Spectropolarimeter. All the experiments were performed using Hellma Suprasil 110-QS 0.1 cm cuvettes. For each peptide, the measurements were performed in phosphate buffer (PB, pH=7.2, 7 mM), 5% TFE in PB (pH=7.2, 7 mM), 10%, 15% and 20% respectively and in 5 mM DPC. The buffer was degassed for 10 min under high vacuum before each set of experiments. The concentration of the peptides was 0.100 mg/mL and each sample was measured in one accumulation. The scan rate was 20 nm/min, pitch 0.5 nm, response 16 sec and bandwidth 1.0 nm. The nitrogen flow was kept >8.5 L/min. After each measurement, the cuvettes were washed successively with 1 M HCl, milli-Q H_2O and PB buffer. The baseline was recorded under the same conditions and subtracted manually.

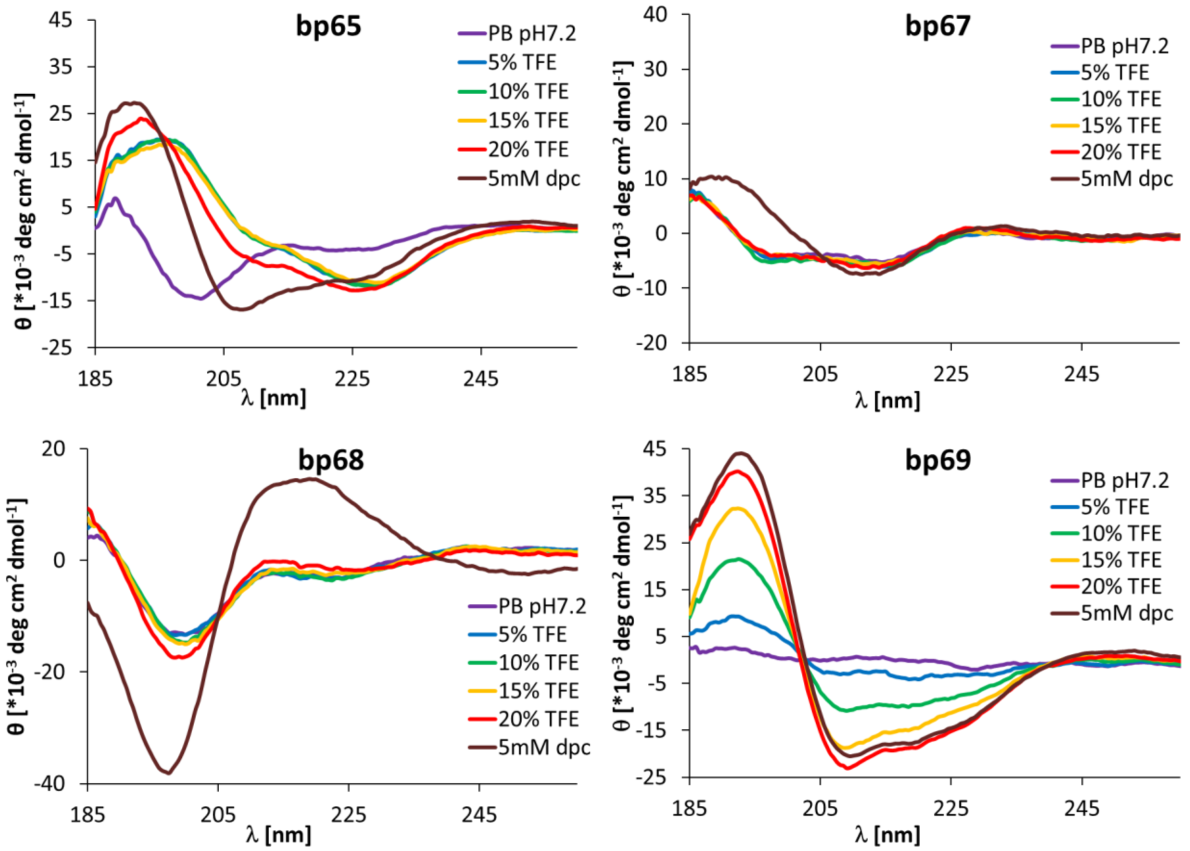

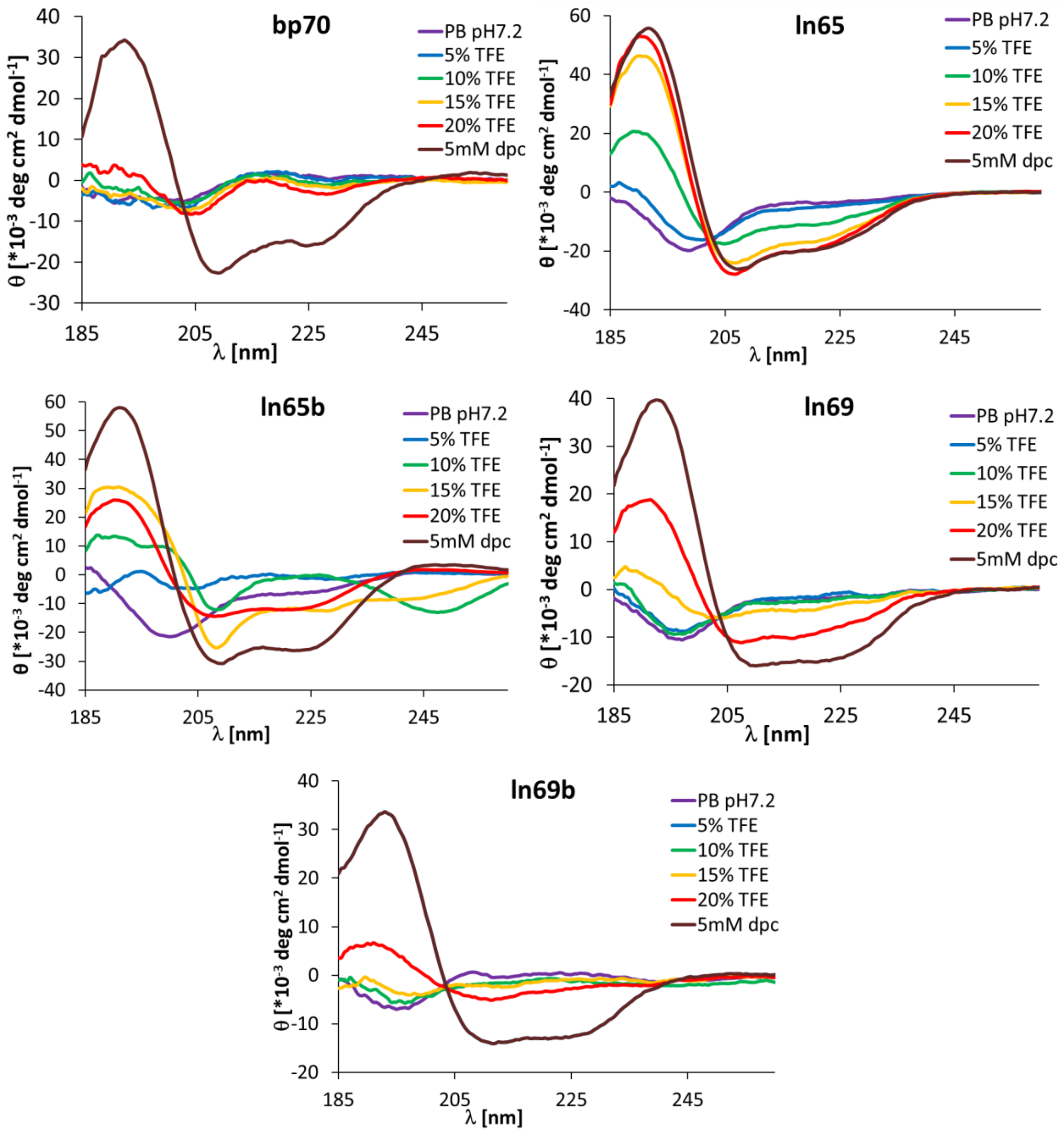

Figure S3: Circular dichroism spectra of linear and bicyclic peptides at 100 µg/mL in phosphate buffer pH 7.2 with different amounts of TFE and 5mM DPC.

Table S2: Dichroweb analysis of **bp65** bicyclic and linear peptide derivatives.

**Cpd.**	**Sequence**[a)]	**CD α/β/t/u (%)**[b)]					
		**0% TFE**	**5% TFE**	**10% TFE**	**15% TFE**	**20% TFE**	**5 mM DPC**
**bp65**	B12KKLLKC1LKC2LL	13/27/22/37	36/25/16/22	37/24/16/23	34/27/17/22	40/20/17/23	46/8/18/29
**ln65b**	TolKKLLKC$_m$LKC$_m$LL	17/19/25/39	22/61/17/0	48/18/34/0	74/5/19/2	74/0/5/21	89/0/0/11
**ln65**	KKLLKLLKLLL	16/20/29/36	18/24/25/33	35/15/23/27	55/4/20/21	61/3/17/20	67/3/16/13
**bp67**	B12kKlLkC1lKc2Ll	3/40/23/34	2/40/23/35	2/40/23/34	2/41/23/33	2/41/24/33	5/40/23/32
**bp68**	B12KKLLKc1LKc2LL	7/31/21/40	8/32/21/39	9/29/23/39	9/30/22/39	10/30/22/38	28/33/13/26
**bp69**	B12kkLLkLC1LkC2LL	9/38/22/31	15/36/21/28	31/25/18/26	46/16/16/22	56/9/14/21	56/12/13/19
**ln69b**	TolkkLLkLC$_m$LkC$_m$LL	9/35/24/32	8/36/24/32	9/35/25/31	9/34/25/32	17/29/26/28	54/12/15/19
**ln69**	kkLLkLLkLLL	10/31/24/35	12/32/25/32	10/30/25/35	16/28/25/31	34/20/20/26	61/6/16/17
**bp70**	B12HONleYDabC1IRC2YA	9/34/23/35	12/31/22/35	14/30/22/34	17/28/23/32	25/28/20/32	73/0/8/19

a) One letter codes for amino acids, B = 3,5-bis(methylene)toluoyl, Nle = norleucine, Dab = diaminobutyric acid, Tol = p-toluic acid, C$_m$ = S-methyl-L-cysteine. Line notation for bicyclic structures uses single letter codes for amino acids and the SMILES convention for cyclization points. b) CD spectra were recorded at 0.100 mg/mL in aq. 7 mM phosphate buffer pH 7.2 with addition of 0, 5, 10, 15 and 20 % v/v TFE or 5 mM DPC. The primary CD spectra were analyzed using DichroWeb, and the percentages of α-helical (α), β-sheet (β), turns (t) and unordered (u) signals were extracted. The Contin-LL (Provencher & Glockner) method and reference set 4 were used.[4]

7 Crystallography

Suitable diffracting crystals were obtained via three different methods: crystallization of the racemic compounds in a 50/50 ratio, direct crystallization of the peptide and co-crystallization of the C-fucosylated derivative with the bacterial lectin LecB.

Racemic and direct crystallization of the bicyclic peptides was performed in two steps. A first round was done using the sitting drop vapor diffusion method, screening 192 different conditions per compound. The peptides were dissolved in milli-Q water at concentrations between 15 and 25 mg/mL and mixed with reservoir solutions in a 1.5 µL to 1.5 µL ratio. Conditions yielding promising hits were further optimized using the vapor diffusion hanging drop method by screening for concentration, additives, pH and temperature. Drop volumes were increased to 6 µL.

Co-crystals of LecB·C-fucosylated peptides were obtained using the sitting drop vapor diffusion method. The lyophilized protein was dissolved in milli-Q water (5 mg/ml) in the presence of salts (6 mM $CaCl_2$ and $MgCl_2$). The peptides were added to the protein at a 5:1 molar excess related to the LecB lectin monomer. 192 different conditions were tested for each peptide-protein combination. Crystals were obtained within five to thirty days after mixing 1.5 µL of LecB ligand-complex with 1.5 µL of reservoir solution at 18 °C.

Primary crystallization conditions were found in Index screens I/II (96 conditions) and Crystal Screen I/II (96 conditions) (Hampton Research, Laguna Niguel, CA, USA). Diffraction data were collected at 80 K at the Paul Scherrer Institute (Villigen, Switzerland) on beamline X06DA PX-III using a DECTRIS PILATUS 2M-F detector and a multi-axis PRIGo goniometer. The structures were solved and visualized with the help of olex2,[5] Phenix,[6] ccp4,[7] PyMol,[8] coot[9] and XDS.[10]

Table S3.1: Data collection and refinement statistics for the X-ray structure of the **bp65/dbp65** bicyclic peptides.

**Structural data**	**bp65/dbp65 racemate in P -1**
Beam line	PSI PXIII
Wavelength(Å)	0.80
Resolution(Å)	50.0-0.8 (0.813-0.809)[a)]
**Cell dimension**	
Space group	2, P -1
Unit cell(Å)	a = 16.5, b = 26.2, c = 29.9;<br>α = 111.3°, β = 92.3°, γ = 102.0°
Measured reflection / unique	51859/29455
Average multiplicity	1.8 (1.8)
Completeness (%)	87.7 (84.7)
Average $I / \sigma(I)$	4.5 (2.2)
Correlation CC (1/2) (%)	92.5 (88.6)
R_{meas} (%)	23.3 (34.9)
Wilson B-factor (Å^2)	7.05
**Refinement**	
Resolution range (Å)	27.66-0.90
R1 (%)	19.13
wR2 (%)	47.73
Average Biso (Å2)	23.8
RMSD from ideality angles (°)	0.822
Bonds (Å)	0.006
Water molecules	10
Ligand molecules	2
Protein Data Bank deposition code	6Y14

a) Values in brackets correspond to the outer shell.

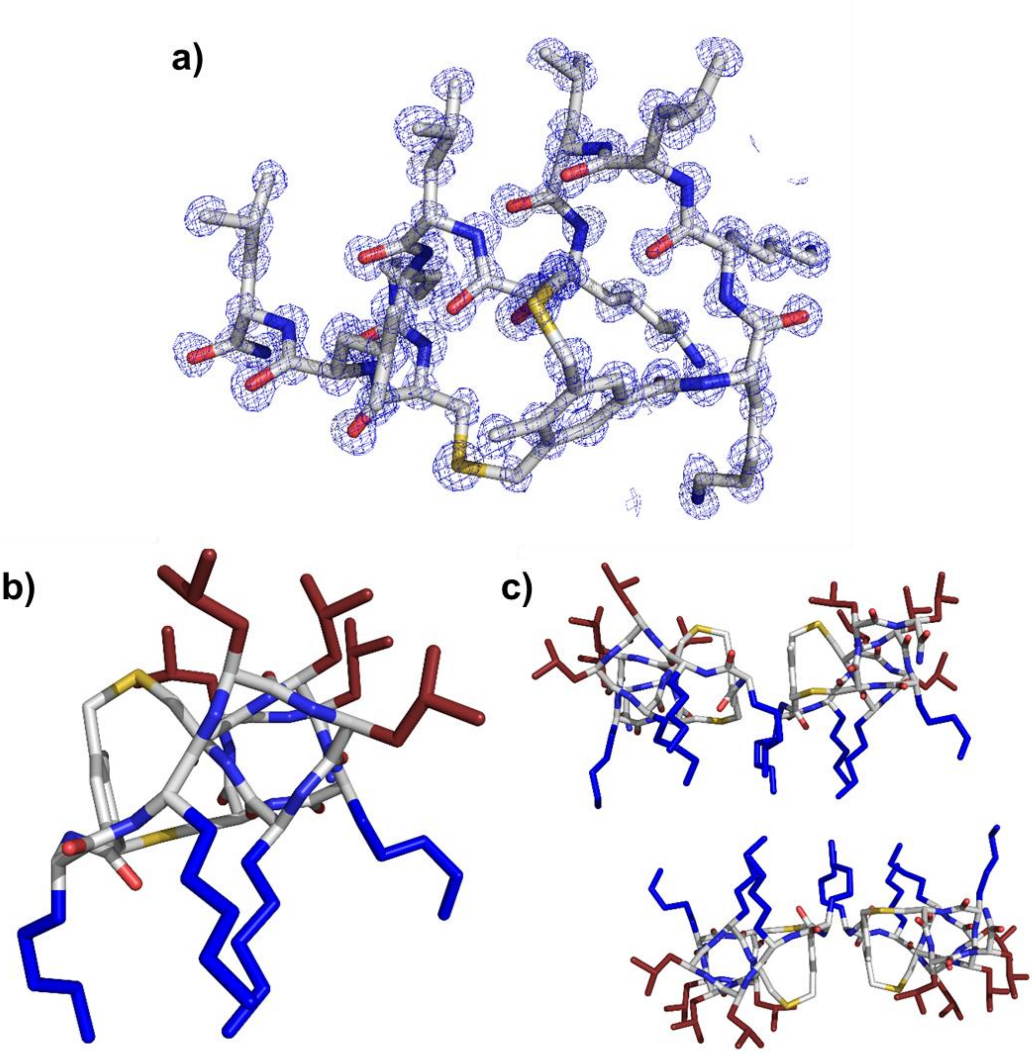

Figure S4.1: Details of the X-ray structure of **bp65** crystallized as racemic mixture. **a**) Stick model of the L-peptide with corresponding electron density map as blue mesh. Charge distribution within the helix of a single L-peptide (**b**) and within the unit cell containing 2 L- and 2 D-peptides (**c**). Amino acid side chains are color-coded. Brown: leucine, blue: lysines.

Table S3.2: Data collection and refinement statistics for the X-ray structure of the **bp70** bicyclic peptide.

**Structural data**	**bp70 in P321**
Beam line	PSI PXIII
Wavelength(Å)	0.80
Resolution(Å)	50.0-1.1 (1.12-1.11)[a)]
**Cell dimension**	
Space group	150, P 3 2 1
Unit cell(Å)	*a* = 26.9, *b* = 26.9, *c* = 26.2;<br>α = 90°, β = 90°, γ = 120°
Measured reflection / unique	49054/7887
Average multiplicity	6.2 (3.4)
Completeness (%)	94.7 (67.2)
Average $I / \sigma(I)$	2.67 (0.79)
Correlation CC (1/2) (%)	97.2 (16.3)
R_{meas} (%)	7.5 (84.4)
Wilson B-factor (Å^2)	13.1
**Refinement**	
Resolution range (Å)	26.16-1.11
R1 (%)	10.34
wR2 (%)	26.89
Goodness of Fit	0.929
RMSD from ideality angles (°)	4.020
Bonds (Å)	0.023
Water molecules	0
Ligand molecules	1
Protein Data Bank deposition code	6Y13

a) Values in brackets correspond to the outer shell.

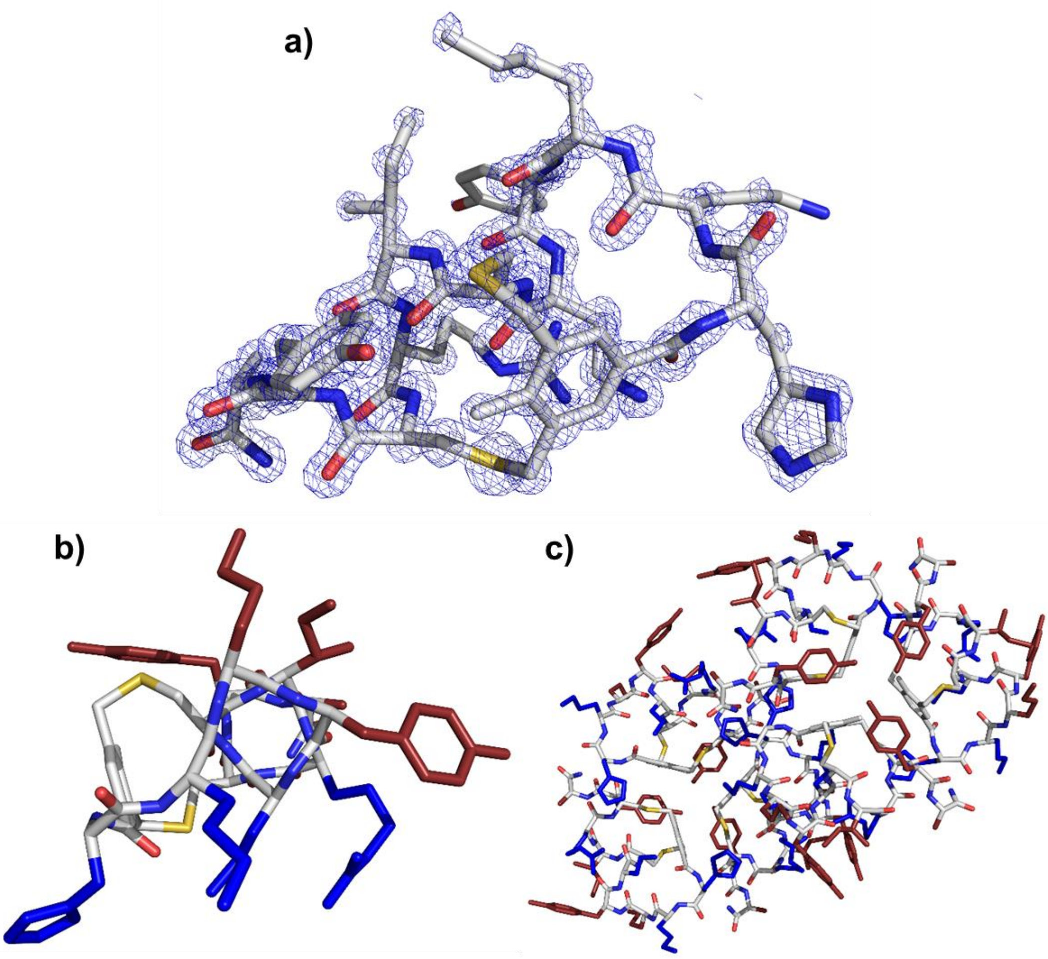

Figure S4.2: Details of the X-ray structure of **bp70** in **P321** crystallized as single L-peptide. (**a**) Stick model with corresponding electron density map as blue mesh. (**b**) Amphiphilic arrangement of leucine and lysine residues along the α-helix. (**c**) View of the unit cell containing 6 symmetrical peptides. Amino acid side chains are color-coded. Brown: hydrophobic, blue: cationic.

Table S3.3: Data collection and refinement statistics for the X-ray structure of the **bp70** bicyclic peptide.

**Structural data**	**bp70 in $I4_132$**
Beam line	PSI PXIII
Wavelength(Å)	1.00
Resolution(Å)	50.0-1.0 (1.06-1.04)[a)]
**Cell dimension**	
Space group	214, $I\ 4_1\ 3\ 2$
Unit cell(Å)	a = 55.5, b = 55.5, c = 55.5;<br>α = 90°, β = 90°, γ = 90°
Measured reflection / unique	470745/7245
Average multiplicity	65.0 (19.1)
Completeness (%)	99.9 (98.6)
Average $I / \sigma(I)$	51.2 (1.00)
Correlation CC (1/2) (%)	100.0 (34.5)
R_{meas} (%)	4.7 (300.6)
Wilson B-factor (Å^2)	19.4
**Refinement**	
Resolution range (Å)	39.22-1.04
R1 (%)	22.53
wR2 (%)	57.41
Average Biso (Å2)	23.8
RMSD from ideality angles (°)	3.72
Bonds (Å)	0.021
Water molecules	0
Ligand molecules	1
Protein Data Bank deposition code	6Y1S

a) Values in brackets correspond to the outer shell.

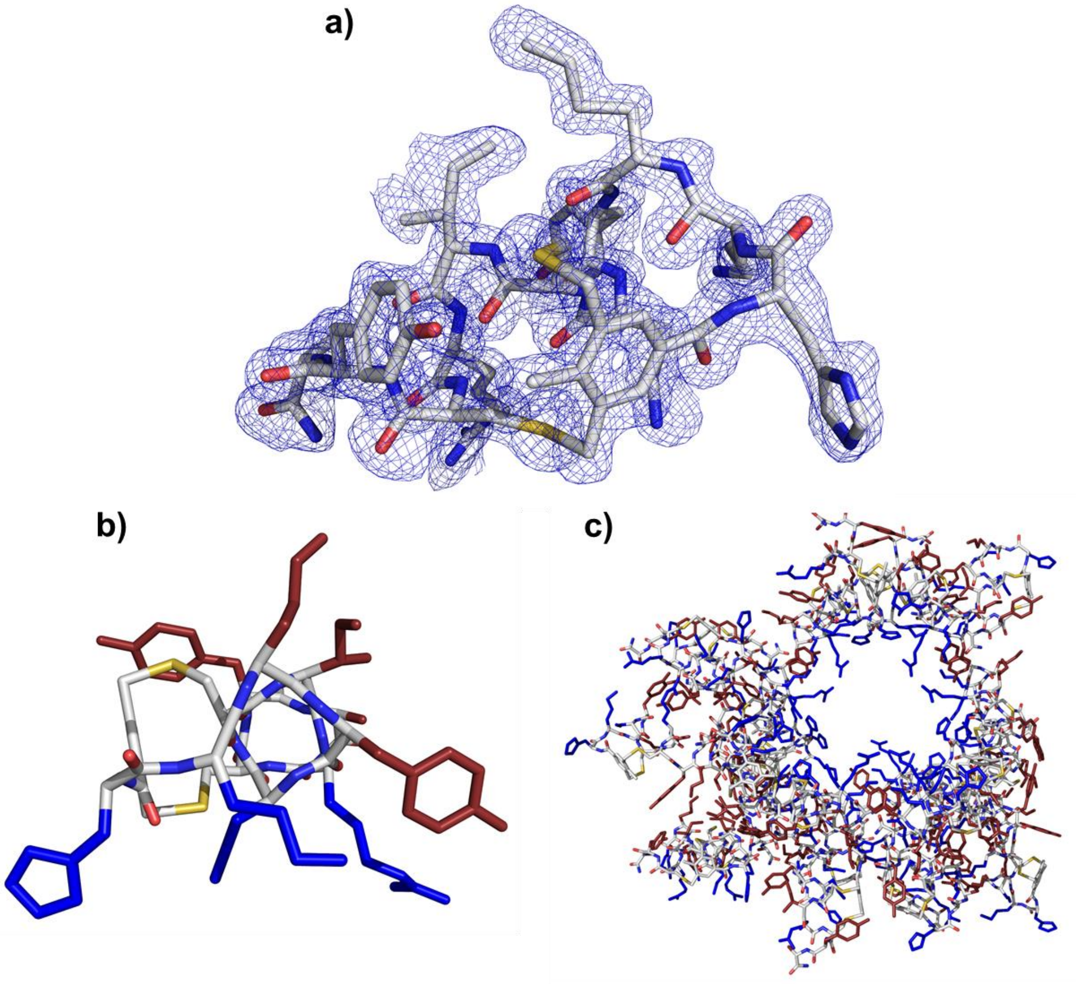

Figure S4.3: Details of the X-ray structure of **bp70** in **I4₁32** crystallized as single L-peptide. (**a**) Stick model with corresponding electron density map as blue mesh. (**b**) Amphiphilic arrangement of leucine and lysine residues along the α-helix. (**c**) view of the unit cell containing 24 symmetrical peptides. Amino acid side chains are color-coded. Brown: hydrophobic, blue: cationic.

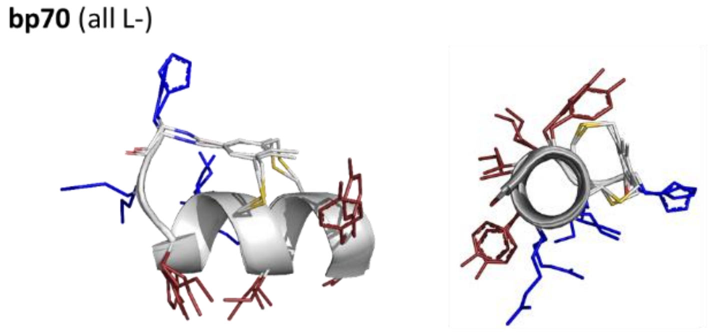

Figure S4.4: Superpositions of non-equivalent peptides within the X-ray structures of **bp70** (PDB entries 6Y13 and 6Y1S). Peptides are shown as grey cartoon with the side chains as sticks. Amino acid side chains are color-coded: brown = hydrophobic, blue = cationic.

Table S3.4: Data collection and refinement statistics for the X-ray structure of the **bp71**·LecB complex.

**Structural data**	**bp71·LecB in P 2_1 2_1 2**
Beam line	PSI PXIII
Wavelength(Å)	1.00
Resolution(Å)	50.0-1.5 (1.53-1.49)[a)]
**Cell dimension**	
Space group	18, P 2_1 2_1 2
Unit cell(Å)	*a* = 122.8, *b* = 56.6, *c* = 70.5;<br>α = 90°, β = 90°, γ = 90°
Measured reflection / unique	251455/144478
Average multiplicity	1.7 (1.6)
Completeness (%)	92.9 (88.3)
Average $I / \sigma(I)$	8.04 (1.31)
Correlation CC (1/2) (%)	99.7 (57.8)
R_{meas} (%)	7.5 (84.4)
Wilson B-factor (Å^2)	23.6
**Refinement**	
Resolution range (Å)	46.29-1.49
Rwork (%)	16.82
Rfree (%)	19.19
Average Biso (Å2)	23.8
RMSD from ideality angles (°)	0.822
Bonds (Å)	0.006
Water molecules	484
Ligand molecules	4
Protein Data Bank deposition code	6Y0U

a) Values in brackets correspond to the outer shell.

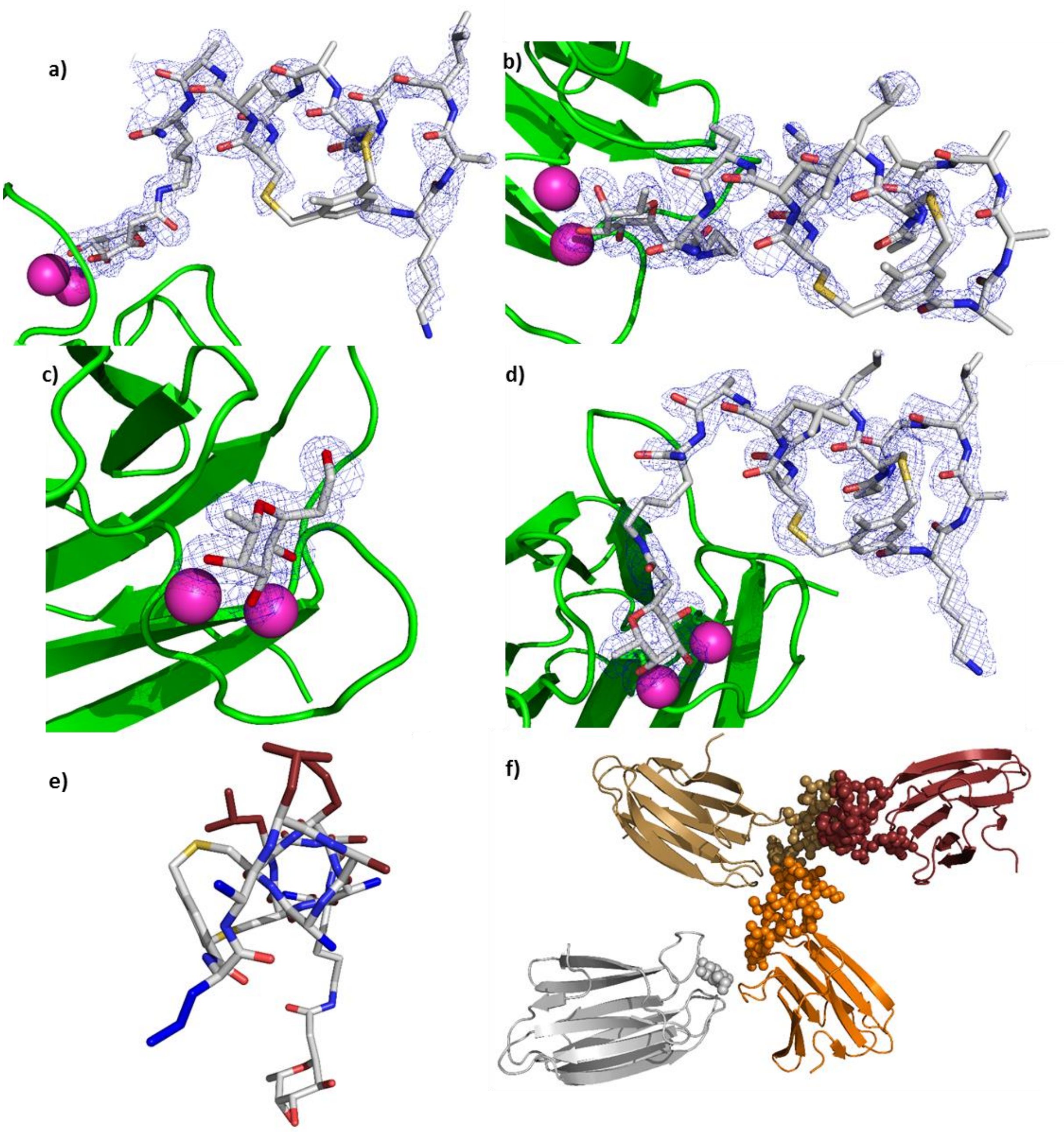

Figure S4.5: Details of the X-ray structure of the **bp71**·LecB complex in P 2_1 2_1 2. (**a-d**) Asymmetric peptide entities with corresponding electron density map as blue mesh, with Ca^{2+} atoms shown as magenta spheres and the bound LecB monomer as green cartoon. (**e**) Amphiphilic arrangement of leucine and lysine residues along the α-helix. Amino acid side chains are color-coded. Brown: leucine, blue: lysines. (**f**) View of the unit cell including LecB subunits and the bound peptides. Peptides are shown as spheres and bound lectin monomers are displayed as cartoon of the same color.

Table S3.5: Data collection and refinement statistics for the X-ray structure of the second **bp71**·LecB complex.

**Structural data**	**bp71·LecB in P 1 2_1 1**
Beam line	PSI PXIII
Wavelength(Å)	1.00
Resolution(Å)	50.0-1.7 (1.76-1.75)[a)]
**Cell dimension**	
Space group	4, P 1 2_1 1
Unit cell(Å)	*a* = 45.1, *b* = 104.9, *c* = 53.0;<br>α = 90°, β = 93.4°, γ = 90°
Measured reflection / unique	164442/92928
Average multiplicity	1.8 (1.7)
Completeness (%)	93.8 (93.1)
Average $I / \sigma(I)$	6.24 (0.92)
Correlation CC (1/2) (%)	99.7 (46.1)
R_{meas} (%)	11.9 (115.0)
Wilson B-factor (Å^2)	26.3
**Refinement**	
Resolution range (Å)	47.23-1.75
Rwork (%)	17.91
Rfree (%)	23.26
Average Biso (Å2)	26.3
RMSD from ideality angles (°)	0.796
Bonds (Å)	0.007
Water molecules	329
Ligand molecules	4
Protein Data Bank deposition code	6Y0V

a) Values in brackets correspond to the outer shell.

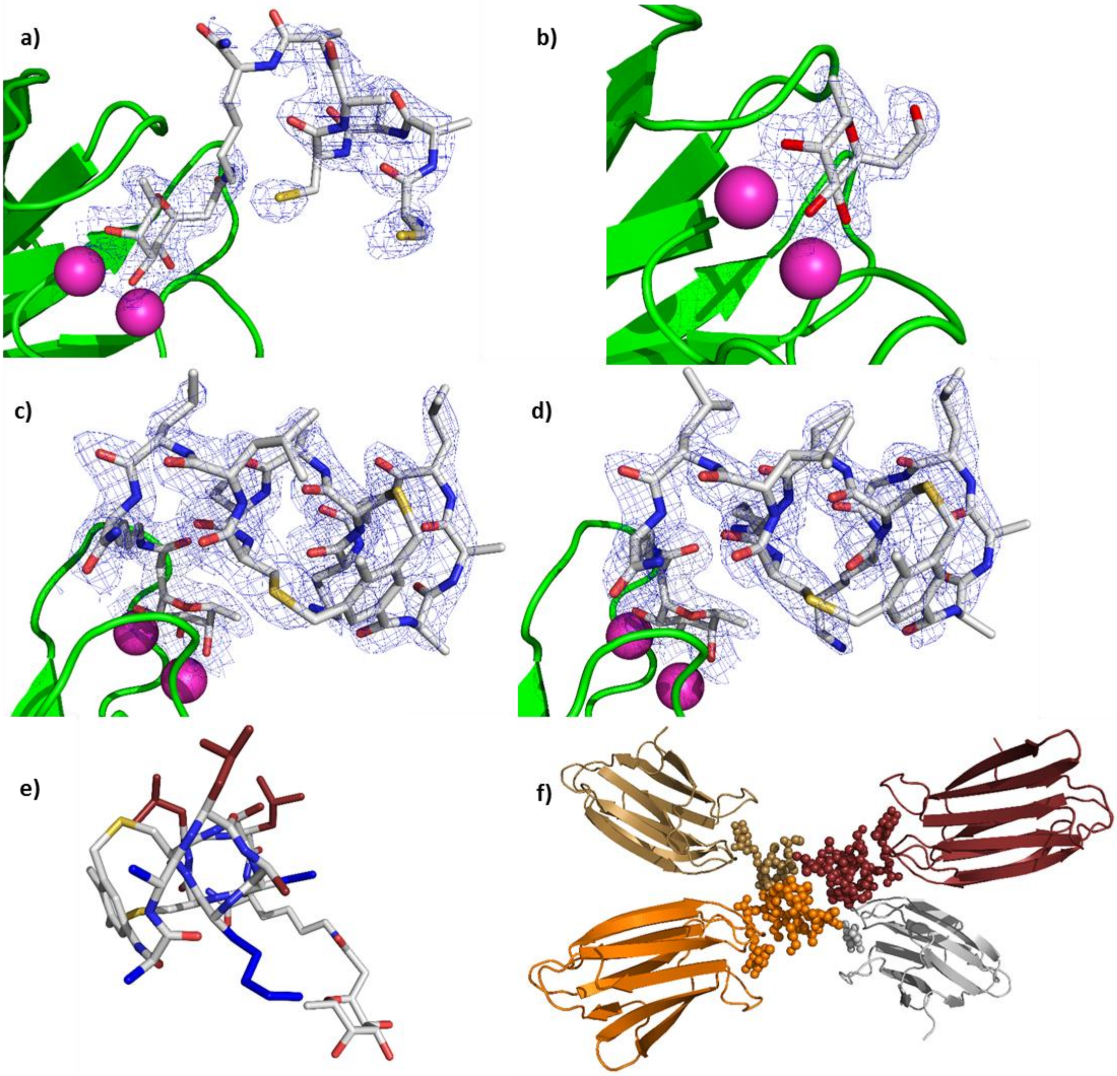

Figure S4.6: Details of the X-ray structure of the second **bp71**·LecB complex in P 1 2_1 1. (**a-d**) Asymmetric peptide entities with corresponding electron density map as blue mesh, with Ca^{2+} atoms shown as magenta spheres and the bound LecB monomer as green cartoon. (**e**) Amphiphilic arrangement of leucine and lysine residues along the α-helix. Amino acid side chains are color-coded. Brown: leucine, blue: lysine. (**f**) View of the unit cell including LecB subunits and the bound peptides. Peptides are shown as spheres and bound lectin monomers are displayed as cartoon of the same color.

Table S3.6: Data collection and refinement statistics for the X-ray structure of the **Fln65**·LecB complex.

**Structural data**	**Fln65· LecB**
Beam line	PSI PXIII
Wavelength(Å)	0.976
Resolution(Å)	48.6-1.5 (1.515-1.507)[a)]
**Cell dimension**	
Space group	4, P 1 2_1 1
Unit cell(Å)	a = 74.5, b = 64.3, c = 118.9;<br>α = 90°, β = 94.9°, γ = 90°
Measured reflection / unique	1071279/336482
Average multiplicity	3.2 (2.4)
Completeness (%)	96.2 (89.8)
Average $I / \sigma(I)$	8.23 (1.23)
Correlation CC (1/2) (%)	99.7 (68.5)
R_{meas} (%)	11.7 (93.3)
Wilson B-factor ($Å^2$)	12.8
**Refinement**	
Resolution range (Å)	48.59-1.51
R_{work} (%)	18.21
R_{free} (%)	20.60
Average Biso ($Å^2$)	89.1
RMSD from ideality angles (°)	1.025
Bonds (Å)	0.008
Water molecules	1415
Ligand molecules	8
Protein Data Bank deposition code	7NEF

a) Values in brackets correspond to the outer shell.

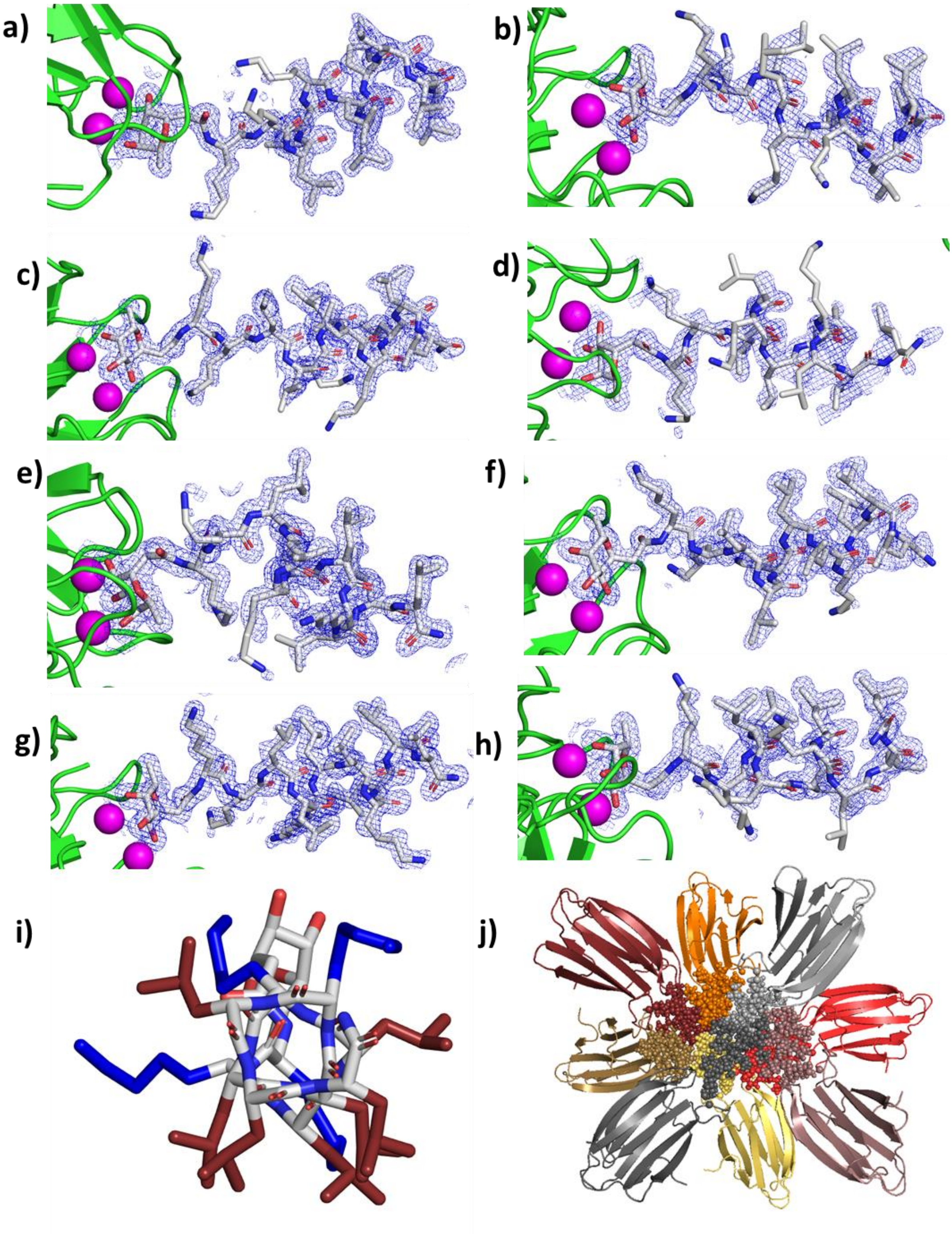

Figure S4.7: Details of the X-ray structure of the **Fln65**·LecB complex in P 1 2_1 1. (**a-h**) Asymmetric peptide entities with corresponding electron density map as blue mesh, with Ca^{2+} atoms shown as magenta spheres and the bound LecB monomer as green cartoon. (**i**) Amphiphilic arrangement of leucine and lysine residues along the α-helix. Amino acid side chains are color-coded. Brown: leucine, blue: lysine. (**j**) View of the unit cell including LecB subunits and the bound peptides. Peptides are shown as spheres and bound lectin monomers are displayed as cartoon of the same color.

Table S3.7: Data collection and refinement statistics for the X-ray structure of the **Fdln69**·LecB complex.

**Structural data**	**Fdln69· LecB**
Beam line	PSI PXIII
Wavelength(Å)	0.976
Resolution(Å)	48.3-2.0 (2.027-2.017)[a)]
**Cell dimension**	
Space group	5, C 1 2 1
Unit cell(Å)	a = 130.6, b = 64.8, c = 73.6;<br>α = 90°, β = 113.3°, γ = 90°
Measured reflection / unique	252919/71291
Average multiplicity	3.5 (3.5)
Completeness (%)	97.5 (96.1)
Average $I / \sigma(I)$	6.89 (1.01)
Correlation CC (1/2) (%)	99.5 (51.2)
R_{meas} (%)	16.7 (139.3)
Wilson B-factor (Å^2)	32.0
**Refinement**	
Resolution range (Å)	40.03-2.02
R_{work} (%)	20.70
R_{free} (%)	22.80
Average Biso (Å^2)	161.2
RMSD from ideality angles (°)	0.702
Bonds (Å)	0.003
Water molecules	236
Ligand molecules	4
Protein Data Bank deposition code	7NEW

a) Values in brackets correspond to the outer shell.

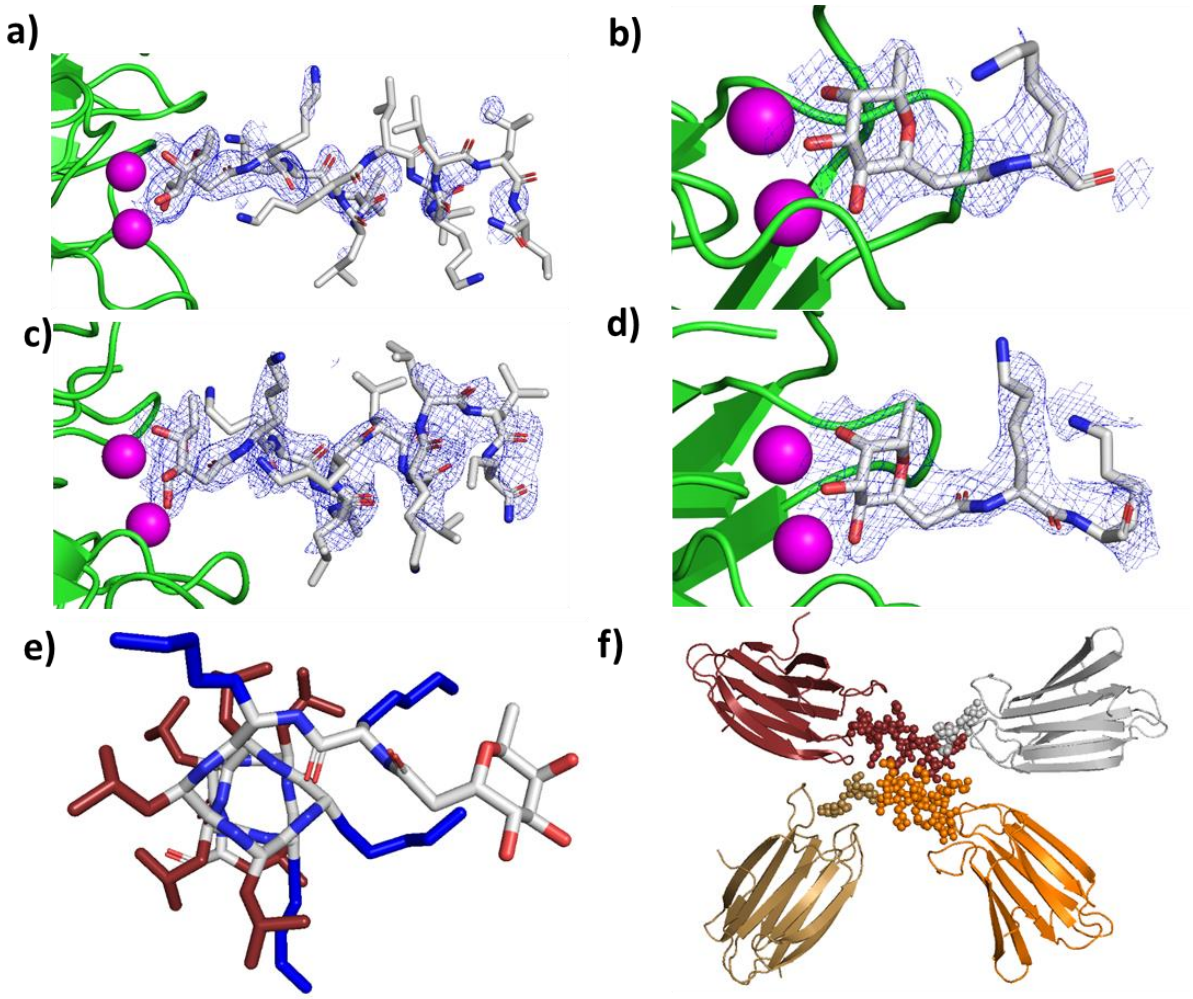

Figure S4.8: Details of the X-ray structure of the **Fdln69**·LecB complex in C 1 2 1. **a-d**) Asymmetric peptide entities with corresponding electron density map as blue mesh, with Ca^{2+} atoms shown as magenta spheres and the bound LecB monomer as green cartoon. **e**) Amphiphilic arrangement of leucine and lysine residues along the α-helix. Amino acid side chains are color-coded. Brown: leucine, blue: lysine. **f**) View of the unit cell including LecB subunits and the bound peptides. Peptides are shown as spheres and bound lectin monomers are displayed as cartoon of the same color.

8 Molecular Dynamics

Molecular dynamics (MD) simulations were performed for the bicyclic and linear peptides **bp65**, **bp69**, **ln65** and **ln69** using GROMACS software version 2018.1 and the gromos53a6 force field.[11,12] The starting topologies were built from the existing X-ray structures. A dodecahedral box was created around the peptide 1.0 nm from the edge of the peptide and filled with extended simple point charge water molecules. Sodium and chloride ions were added to produce an electroneutral solution at a final concentration of 0.15 M NaCl. The energy was minimized using a steepest gradient method to remove any close contacts before the system was subjected to a two-phase position-restrained MD equilibration procedure. The system was first allowed to evolve for 100 ps in a canonical NVT (N is the number of particles, V the system volume, and T the temperature) ensemble at 300 K before pressure coupling was switched on and the system was equilibrated for an additional 100 ps in the NPT (P is the system pressure) ensemble at 1.0 bar.

8.1 Parameters for the non-natural residues

The model for the bicyclic peptide was built by adding the 3,5-bis(chloromethyl)-4-methylbenzoic acid linker (DBZ) to the N-terminus of the linear peptide. Bonds were added between the alkylated cysteine residues (CYX) and the corresponding methyl groups of the DBZ residue leading to the final desired topology. The parameters for the 2 non-natural residues of the Gromos53a6 force field (aminoacids.rtp) were derived from the existing amino acids. They were defined as follows:

```
[ DBZ ]  ; derived from PHE, small positive charge to match CYX
 [ atoms ]
   CA   CH3     0.00000     0
   CG     C     0.00000     0
  CE1     C    -0.14000     1
  HE1    HC     0.14000     1
  CE2     C    -0.14000     2
  HE2    HC     0.14000     2
  CD1     C     0.00000     3
  CB1   CH2     0.24100     4
  CD2     C     0.00000     5
  CB2   CH2     0.24100     6
   CZ     C     0.00000     7
    C     C       0.450     8
    O     O      -0.450     8
 [ bonds ] ;
   CA    CG    gb_27  ; similarly to CB-CG in PHE
   CG   CD1    gb_16
   CG   CD2    gb_16
  CD1   CE1    gb_16
  CD2   CE2    gb_16
  CD1   CB1    gb_27  ; single bond to C
  CD2   CB2    gb_27
  CE1   HE1    gb_3
  CE1    CZ    gb_16
  CE2   HE2    gb_3
  CE2    CZ    gb_16
   CZ     C    gb_27
    C     O    gb_5
    C    +N    gb_10
 [ exclusions ] ; aromatics exclude repulsion penalty 1-4
;  ai    aj
   C    HE1
   C    HE2
   C    CD1
   C    CD2
   CZ   CB1
   CZ   CB2
   CZ    CG
  CE1    CA
  CE1   CD2
  CE1   HE2
  HE1   CE2
  HE1   CB1
  HE1    CG
  CE2   CD1
  CE2    CA
  HE2   CB2
  HE2    CG
  CD1   CB2
  CB1   CD2
  CB1    CA
  CB2    CA
 [ angles ]
;  ai    aj    ak   gromos type
   CA    CG   CD1     ga_27
   CA    CG   CD2     ga_27
  CD1    CG   CD2     ga_27
   CG   CD1   CE1     ga_27
   CG   CD2   CE2     ga_27
   CG   CD1   CB1     ga_27
   CG   CD2   CB2     ga_27
```

```
  CB1   CD1   CE1     ga_27
  CB2   CD2   CE2     ga_27
  CD1   CE1   HE1     ga_25
  CD1   CE1    CZ     ga_27
  HE1   CE1    CZ     ga_25
  CD2   CE2   HE2     ga_25
  CD2   CE2    CZ     ga_27
  HE2   CE2    CZ     ga_25
  CE1    CZ   CE2     ga_27
  CE1    CZ     C     ga_27
  CE2    CZ     C     ga_27
   CZ     C     O     ga_30
   CZ     C    +N     ga_19
    O     C    +N     ga_33
 [ impropers ]
;  ai    aj    ak    al   gromos type
   CG   CD1   CD2    CA     gi_1
   CG   CD1   CE1    CZ     gi_1
   CG   CD2   CE2    CZ     gi_1
  CD1    CG   CD2   CE2     gi_1
  CD1    CG   CE1   CB1     gi_1
  CD1   CE1    CZ   CE2     gi_1
  CD2    CG   CD1   CE1     gi_1
  CD2    CG   CE2   CB2     gi_1
  CD2   CE2    CZ   CE1     gi_1
  HE1   CD1    CZ   CE1     gi_1
  HE2   CD2    CZ   CE2     gi_1
   CZ   CE1   CE2     C     gi_1
    C    CZ    +N     O     gi_1
 [ dihedrals ]
;  ai    aj    ak    al   gromos type
   CZ     C    +N   +CA     gd_14
  CE1    CZ     C    +N     gd_33  ; HC-C-C-

[ CYX ]  ; derived from CYS1 and MET topologies, note that the charge is non-zero since it is
intended to be connected to DBZ residue and match the charge
 [ atoms ]
    N     N    -0.31000     0
    H     H     0.31000     0
   CA   CH1     0.00000     1
   CB   CH2     0.24100     2
   SG     S    -0.48200     3
    C     C       0.450     4
    O     O      -0.450     4
 [ bonds ]
    N     H    gb_2
    N    CA    gb_21
   CA    CB    gb_27
   CA     C    gb_27
   CB    SG    gb_32
    C     O    gb_5
    C    +N    gb_10
 [ angles ]
;  ai    aj    ak   gromos type
   -C     N     H     ga_32
   -C     N    CA     ga_31
    H     N    CA     ga_18
    N    CA    CB     ga_13
    N    CA     C     ga_13
   CB    CA     C     ga_13
   CA    CB    SG     ga_16
   CA     C     O     ga_30
   CA     C    +N     ga_19
    O     C    +N     ga_33
 [ impropers ]
;  ai    aj    ak    al   gromos type
    N    -C    CA     H     gi_1
   CA     N     C    CB     gi_2
    C    CA    +N     O     gi_1
 [ dihedrals ]
;  ai    aj    ak    al   gromos type
  -CA    -C     N    CA     gd_14
   -C     N    CA     C     gd_39
    N    CA    CB    SG     gd_34
    N    CA     C    +N     gd_40
```

8.2 MD in the presence of a DPC micelle

MD simulations in the presence of a DPC (n-dodecylphosphocholine) micelle were performed as follows. Parameters and references for the DPC molecule for the GROMOS53a6 forcefield are given below. Peptides were manually placed at a distance from the pre-equilibrated micelle (of 65 DPC molecules) equal to the diameter of said peptide. Box, solvation and NVT equilibration procedures were performed as explained previously. For each peptide/micelle system, 10 runs of 50 ns were generated to show the possibility for the peptide to either interact or diffuse away from the micelle. Then, runs of interest were extended up to 250 ns.

8.3 Topology for the DPC molecule[13]

```
; Charge from Chiu et al.
; Chiu, S. W.; Clark, M.; Balaji, V.; Subramaniam, S.; Scott, H. L.; Jakobsson, E. Incorporation of
surface tension into molecular dynamics simulation of an interface: a fluid phase lipid bilayer
membrane. Biophys. J. 1995, 69, 1230-1245.
; Atom types from GROMOS53A6
; Oostenbrink, C.; Soares, T. A.; van der Vegt, N. F. A.; van Gunsteren, W. F. Validation of the
53A6 GROMOS force field. Eur. Biophys. J. 2005, 34, 273-284.

[ moleculetype ]
; Name   nrexcl
DPC      3

[ atoms ]
;   nr    type   resnr  residu    atom    cgnr        charge          mass
     1     CH3       1    DPC      C1       1         0.40  15.035  ; qtot: 0.25
     2     CH3       1    DPC      C2       2         0.40  15.035  ; qtot: 0.50
     3     CH3       1    DPC      C3       3         0.40  15.035 ; qtot: 0.75
     4     NL        1    DPC      N4       4        -0.5  14.0067 ; qtot: 0.75
     5     CH2       1    DPC      C5       5         0.30  14.027 ; qtot:  1.0
     6     CH2       1    DPC      C6       6         0.40  14.027 ; qtot: 1.0
     7      OA       1    DPC      O7       7        -0.80  15.999 ; qtot: 0.64
     8       P       1    DPC      P8       8         1.7  30.973 ; qtot : 1.63
     9      OM       1    DPC      O9       9        -0.8 15.999 ; qtot: 0.995
    10      OM       1    DPC      O10     10        -0.8 15.999 ; qtot: 0.36
    11      OA       1    DPC      O11     11        -0.7  15.999 ; qtot: 0.0
    12     CH2       1    DPC      C12     12         0.0   14.027  ; qtot: 0
    13     CH2       1    DPC      C13     13         0.0   14.027  ; qtot: 0
    14     CH2       1    DPC      C14     14         0.0   14.027  ; qtot: 0
    15     CH2       1    DPC      C15     15         0.0   14.027  ; qtot: 0
    16     CH2       1    DPC      C16     16         0.0   14.027  ; qtot: 0
    17     CH2       1    DPC      C17     17         0.0   14.027  ; qtot: 0
    18     CH2       1    DPC      C18     18         0.0   14.027  ; qtot: 0
    19     CH2       1    DPC      C19     19         0.0   14.027  ; qtot: 0
    20     CH2       1    DPC      C20     20         0.0   14.027  ; qtot: 0
    21     CH2       1    DPC      C21     21         0.0   14.027  ; qtot: 0
    22     CH2       1    DPC      C22     22         0.0   14.027  ; qtot: 0
    23     CH3       1    DPC      C23     23         0.0   15.035  ; qtot: 0


[ bonds ]
;  ai    aj  funct            c0            c1            c2            c3

    1     4     2    gb_21
    2     4     2    gb_21
    3     4     2    gb_21
    4     5     2    gb_21
    5     6     2    gb_27
    6     7     2    gb_18
    7     8     2    gb_28
    8     9     2    gb_24
    8    10     2    gb_24
    8    11     2    gb_28
```

```
   11    12     2    gb_18
   12    13     2    gb_27
   13    14     2    gb_27
   14    15     2    gb_27
   15    16     2    gb_27
   16    17     2    gb_27
   17    18     2    gb_27
   18    19     2    gb_27
   19    20     2    gb_27
   20    21     2    gb_27
   21    22     2    gb_27
   22    23     2    gb_27

[ pairs ]
;  ai    aj funct
    1     6     1
    2     6     1
    3     6     1
    4     7     1
    5     8     1
    6     9     1
    6    10     1
    6    11     1
    7    12     1
    8    13     1
    9    12     1
   10    12     1
   11    14     1
;   12    15     1
;   13    16     1
;   14    17     1
;   15    18     1
;   16    19     1
;   17    20     1
;   18    21     1
;   19    22     1
;   20    23     1

[ angles ]
;  ai    aj    ak funct
    1     4     2     2    ga_13
    1     4     3     2    ga_13
    1     4     5     2    ga_13
    2     4     3     2    ga_13
    2     4     5     2    ga_13
    3     4     5     2    ga_13
    4     5     6     2    ga_15
    5     6     7     2    ga_15
    6     7     8     2    ga_26
    7     8     9     2    ga_14
    7     8    10     2    ga_14
    7     8    11     2    ga_5
    9     8    10     2    ga_29
   10     8    11     1    ga_14
    8    11    12     1    ga_26
   11    12    13     1    ga_15
   12    13    14     1    ga_15
   13    14    15     1    ga_15
   14    15    16     1    ga_15
   15    16    17     1    ga_15
   16    17    18     1    ga_15
   17    18    19     1    ga_15
   18    19    20     1    ga_15
   19    20    21     1    ga_15
   20    21    22     1    ga_15
   21    22    23     1    ga_15

[ dihedrals ]
;  ai    aj    ak    al funct
    1     4     5     6     1 gd_29
    4     5     6     7     1 gd_4
    4     5     6     7     1 gd_36
    5     6     7     8     1 gd_29
;
; define gd_20     0.000       5.09          2
; O-P-O- (dna, lipids)  1.2
```

```
 6    7    8    9    1 gd_20
 7    8   11   12    1 gd_27
 8   11   12   13    1 gd_29
11   12   13   14    1 gd_1
12   13   14   15    1 gd_34
13   14   15   16    1 gd_34
14   15   16   17    1 gd_34
15   16   17   18    1 gd_34
16   17   18   19    1 gd_34
17   18   19   20    1 gd_34
18   19   20   21    1 gd_34
19   20   21   22    1 gd_34
20   21   22   23    1 gd_34
```

8.4 Clustering of stable structures

To obtain a representative conformer for each SA-MD run, the last 100 ns (10001 frames) of each run were clustered using an RMSD cut-off adapted to get a good balance between the amount of clusters and the size of the main cluster (100 frames for the whole runs in case of bundles). A large number of clusters combined with a very large percentage of structures in the top cluster is an indication of the stability of the one main conformer in each case. The PyMol Molecular Graphics System, version 1.8 (Schrödinger, LLC), was used to create structural models.

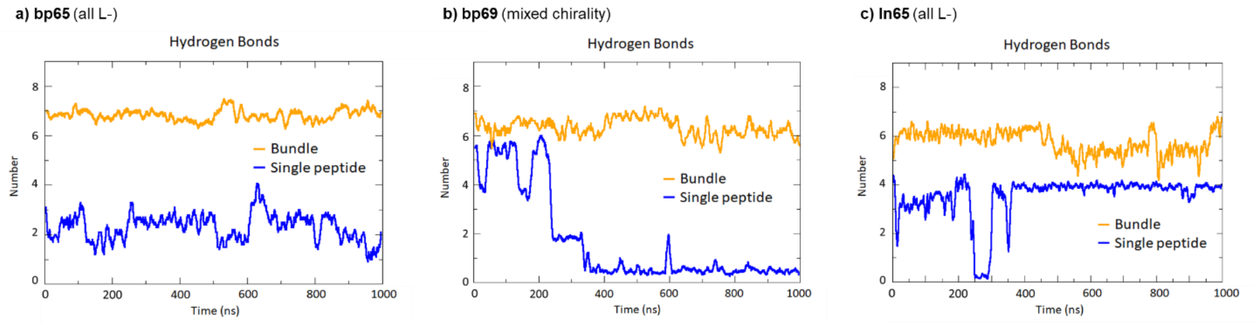

Figure S5: MD simulations of helix bundles for **bp65**, **bp69** and **ln65** from X-ray structures of **bp65**, **bp71** and **Fdln65**. **(a)** Number of intramolecular Hbonds comparison between a single peptide in the bundle compared to single peptide in water in case of **bp65**. **(b)** Same as (a) for **bp69**. **(c)** Same as (a) for **ln65**.

9 HPLC and MS Data

B^{12}KKLLKC^{1}LKC^{2}L (bp3) was obtained as foamy white solid after preparative RP-HPLC (21.6 mg, 15.0 %). Analytical RP-HPLC: t_R = 1.950 min (A/D 100:0 to 0:100 in 7.00 min, λ = 214nm). MS (ESI+): $C_{64}H_{111}N_{15}O_{11}S_2$ calc./obs.1330.81/1330.43 Da $[M+H]^+$.

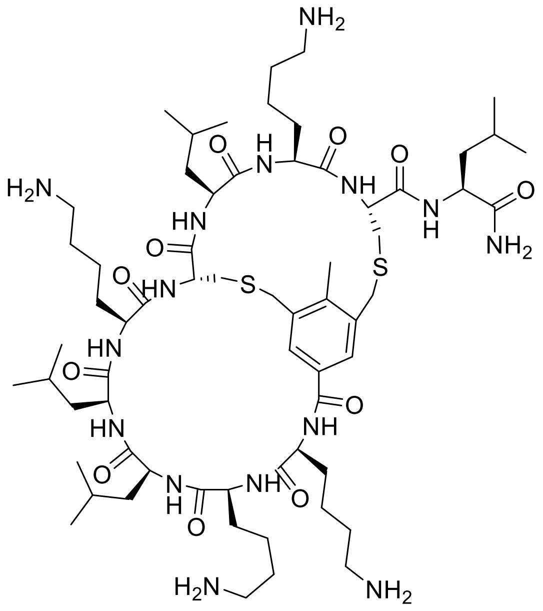

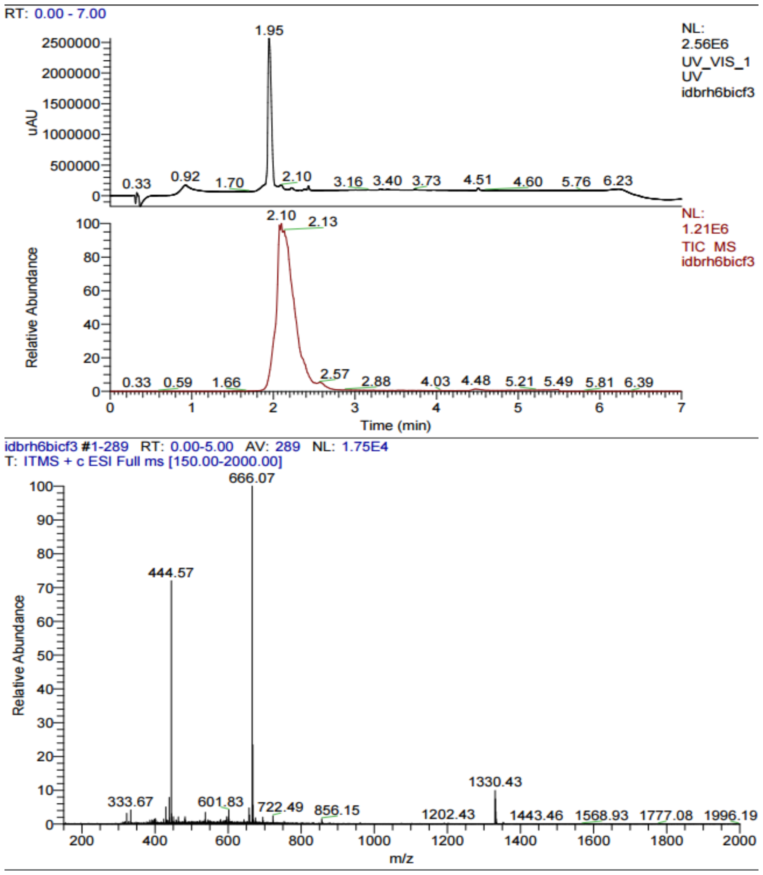

$B^{12}KLLKKC^{1}LKC^{2}L$ (bp59) was obtained as foamy white solid after preparative RP-HPLC (37.4 mg, 26.0 %). Analytical RP-HPLC: t_R = 2.33 min (A/D 100:0 to 0:100 in 7.00 min, λ = 214nm). MS (ESI$^+$): $C_{64}H_{111}N_{15}O_{11}S_2$ calc./obs. 1330.81/1330.73 Da $[M+H]^+$.

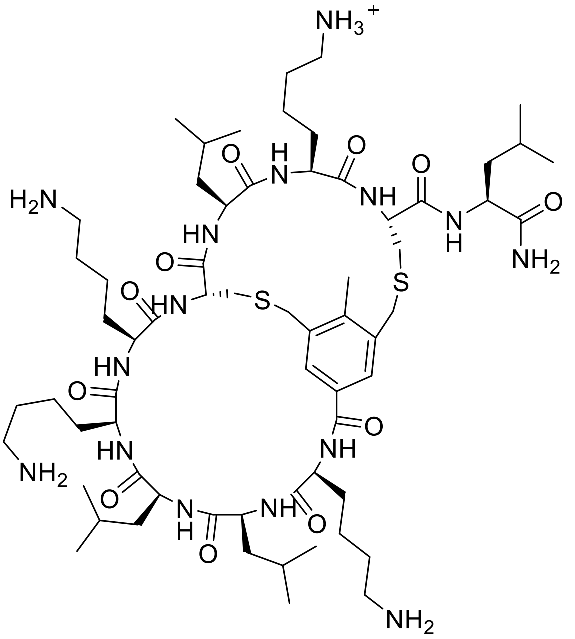

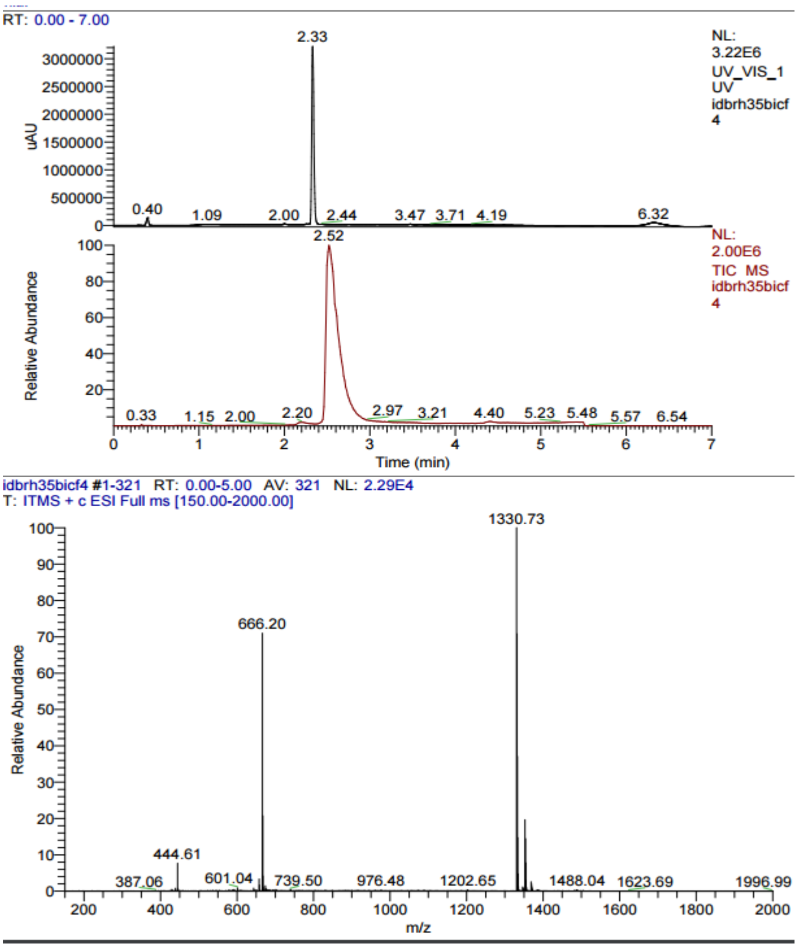

B^{12}KLLKLC^{1}KKC^{2}L (bp60) was obtained as foamy white solid after preparative RP-HPLC (31.2 mg, 21.7 %). Analytical RP-HPLC: t_R = 2.21 min (A/D 100:0 to 0:100 in 7.00 min, λ = 214nm). MS (ESI$^+$): $C_{64}H_{111}N_{15}O_{11}S_2$ calc./obs. 1330.81/1330.70 Da [M+H]$^+$.

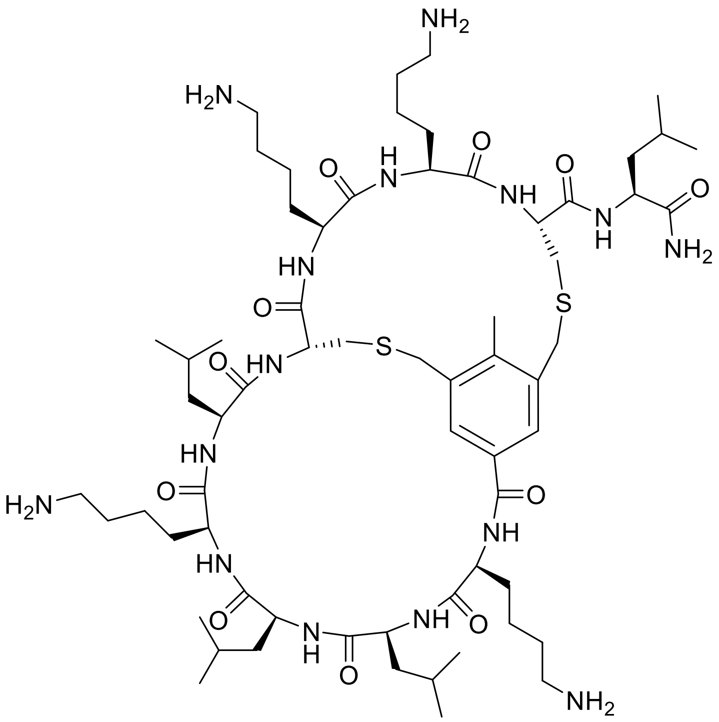

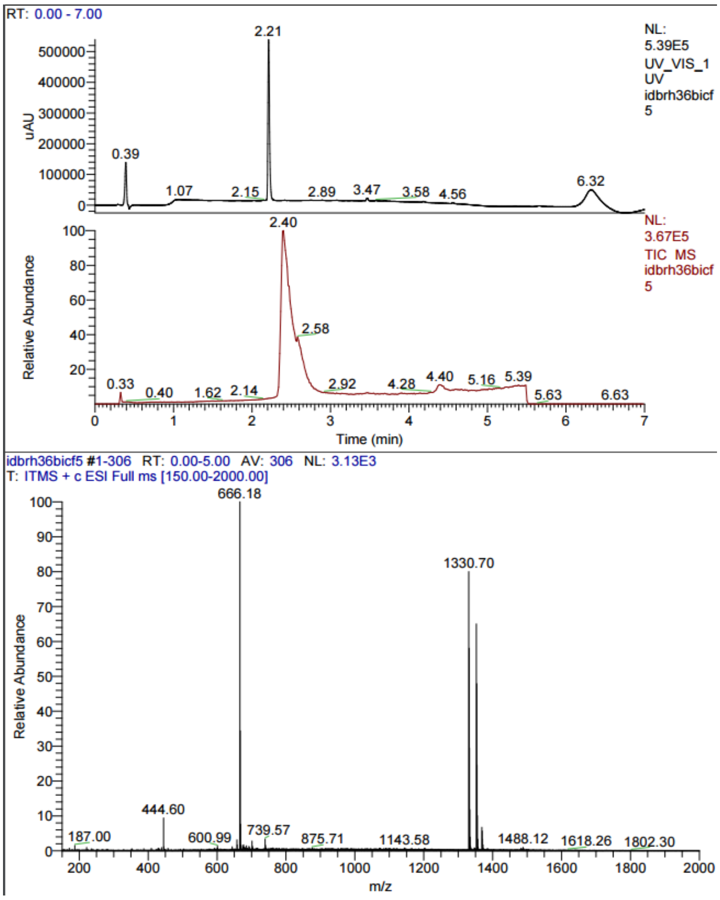

B[12]KKLC[1]KLC[2]KL (bp61) was obtained as foamy white solid after preparative RP-HPLC (41.2 mg, 31.3 %). Analytical RP-HPLC: t_R = 2.00 min (A/D 100:0 to 0:100 in 7.00 min, λ = 214nm). MS (ESI$^+$): $C_{58}H_{100}N_{14}O_{10}S_2$ calc./obs. 1217.73/1217.73 Da $[M+H]^+$.

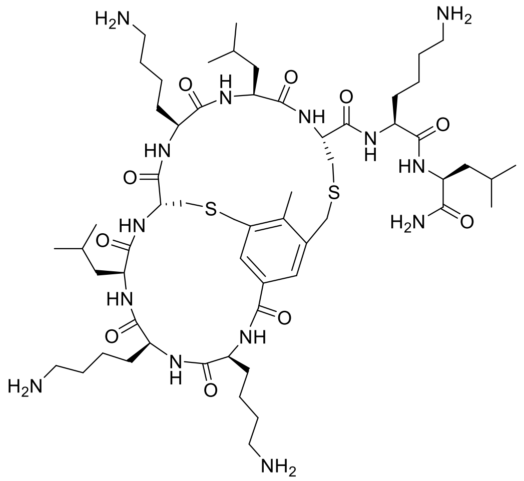

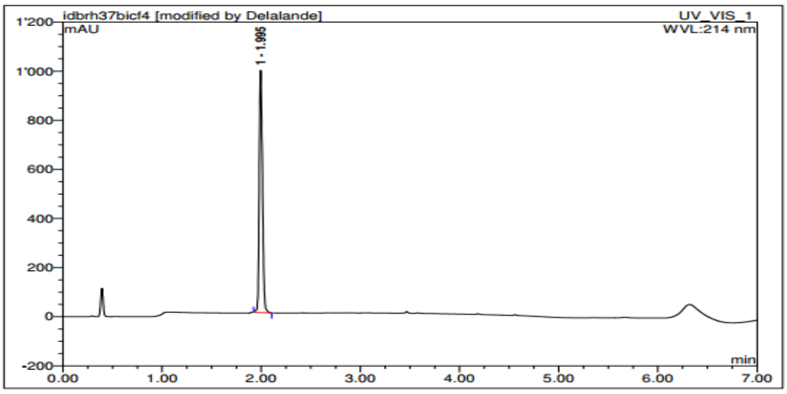

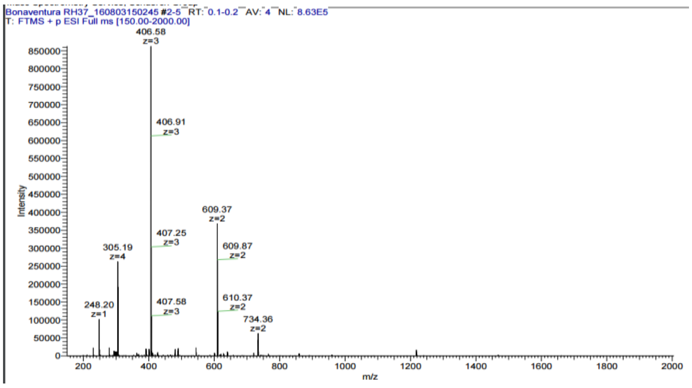

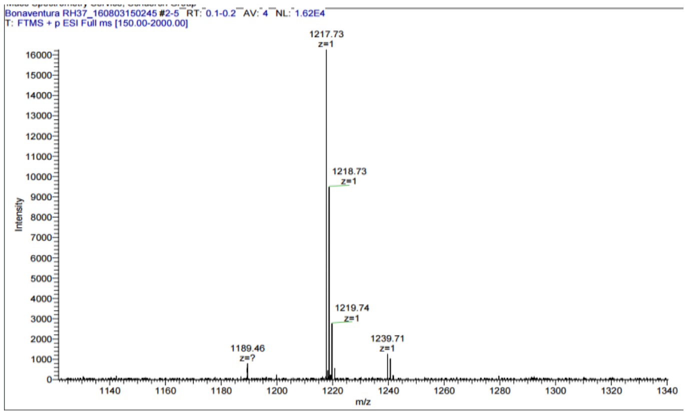

B[12]KKLLLC[1]KLC[2]K (bp62) was obtained as foamy white solid after preparative RP-HPLC (33.5 mg, 23.3 %). Analytical RP-HPLC: t_R = 2.17 min (A/D 100:0 to 0:100 in 7.00 min, λ = 214nm). MS (ESI^+): $C_{64}H_{111}N_{15}O_{11}S_2$ calc./obs. 1330.81/1330.81 Da $[M+H]^+$.

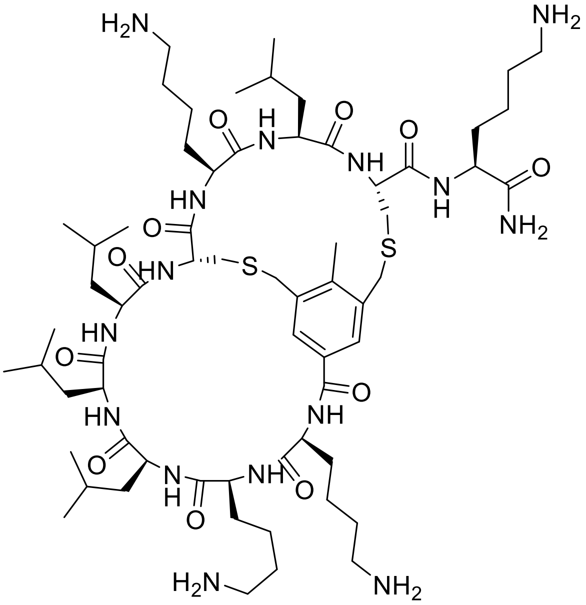

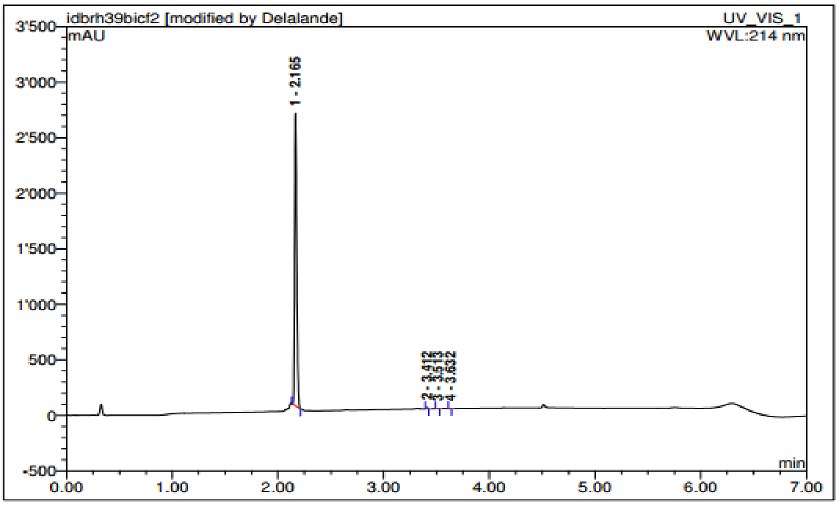

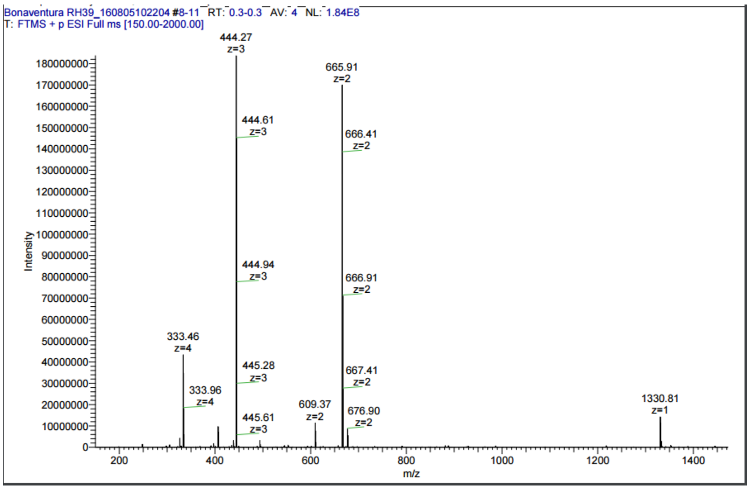

B[12]KKLC[1]KLLKC[2]L (bp63) was obtained as foamy white solid after preparative RP-HPLC (33.5 mg, 23.3 %). Analytical RP-HPLC: t_R = 2.17 min (A/D 100:0 to 0:100 in 7.00 min, λ = 214nm). MS(ESI$^+$): $C_{64}H_{111}N_{15}O_{11}S_2$ calc./obs. 1330.81/1330.81 Da $[M+H]^+$.

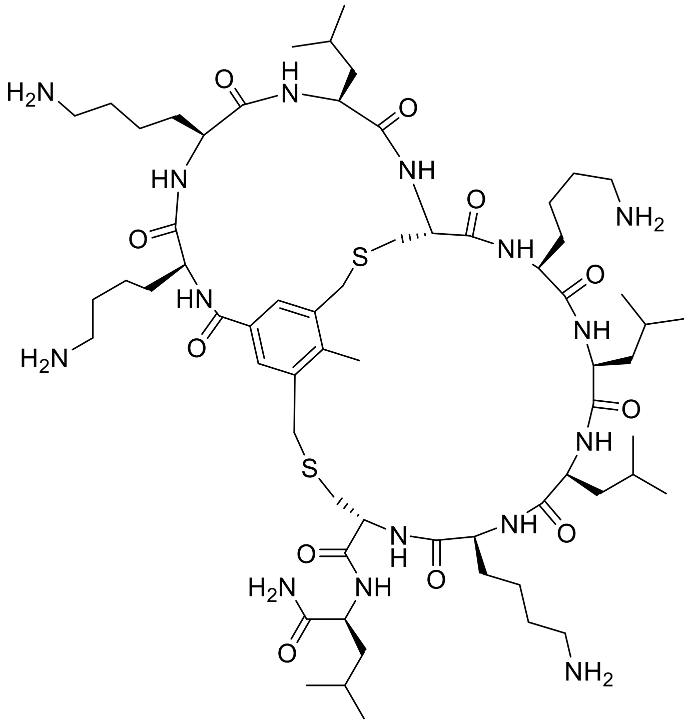

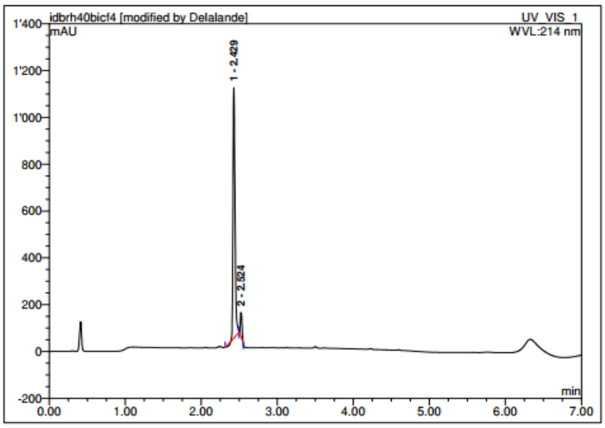

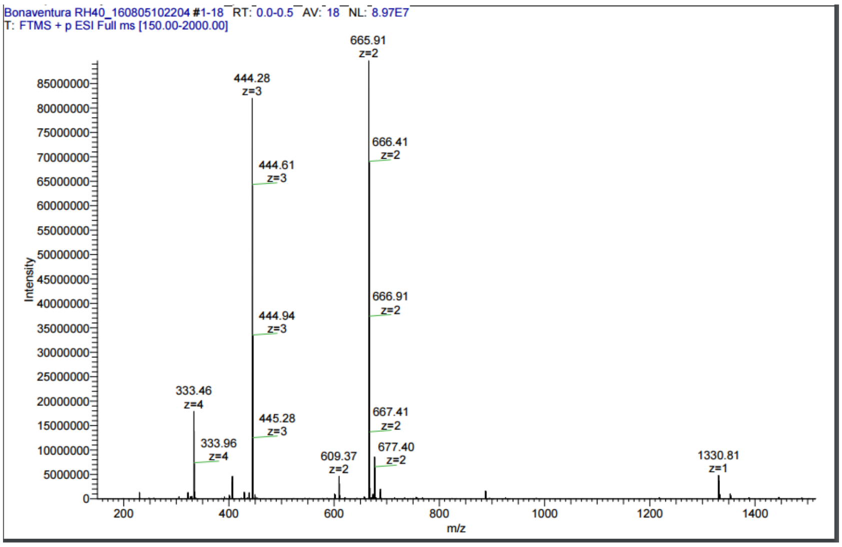

B^{12}KKLLKC^{1}KLC^{2}L (bp64) was obtained as foamy white solid after preparative RP-HPLC (23.5 mg, 16.3 %). Analytical RP-HPLC: t_R = 2.40 min (A/D 100:0 to 0:100 in 7.00 min, λ = 214nm). MS (ESI$^+$): $C_{64}H_{111}N_{15}O_{11}S_2$ calc./obs. 1330.81/1330.71 Da [M+H]$^+$.

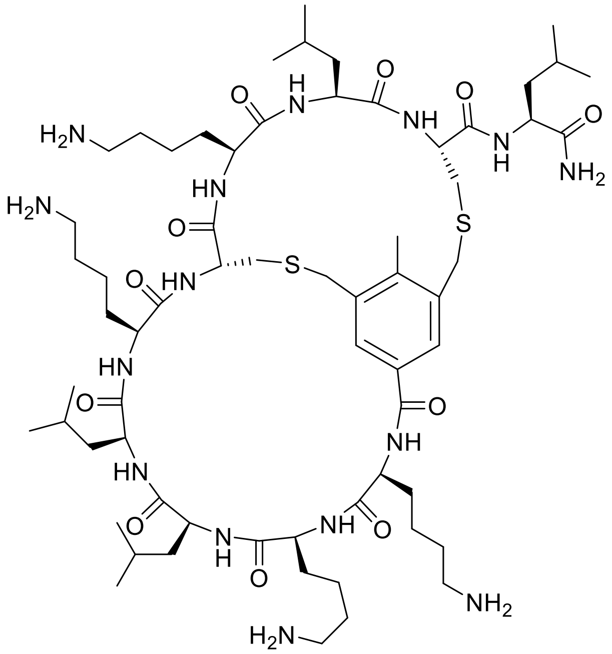

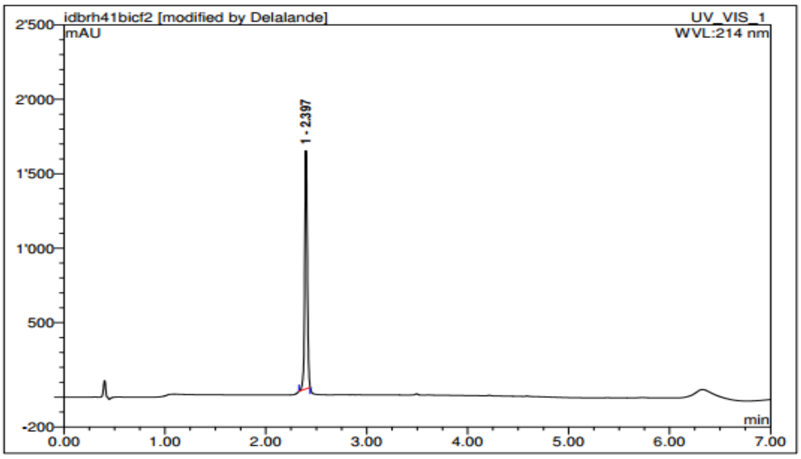

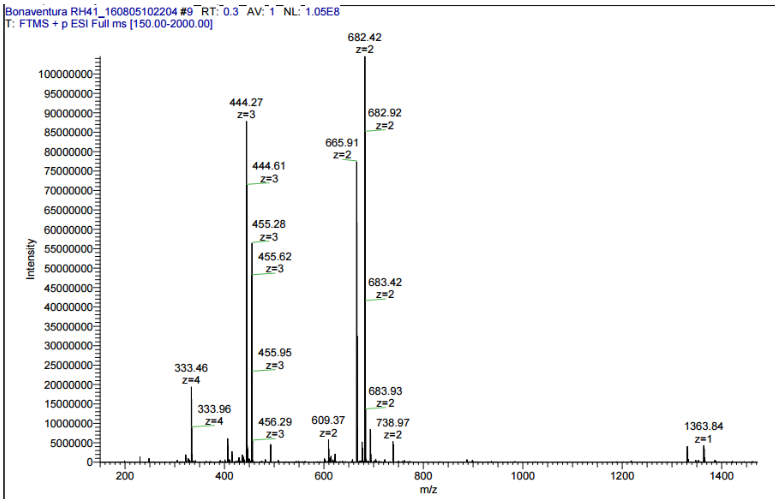

$B^{12}KKLLKC^{1}LKC^{2}LL$ (bp65) was obtained as foamy white solid after preparative RP-HPLC (41.5 mg, 26.5 %). Analytical RP-HPLC: t_R = 2.94 min (A/D 100:0 to 0:100 in 7.00 min, λ = 214nm). MS (ESI$^+$): $C_{70}H_{122}N_{16}O_{12}S_2$ calc./obs. 1443.89/1443.90 Da $[M+H]^+$.

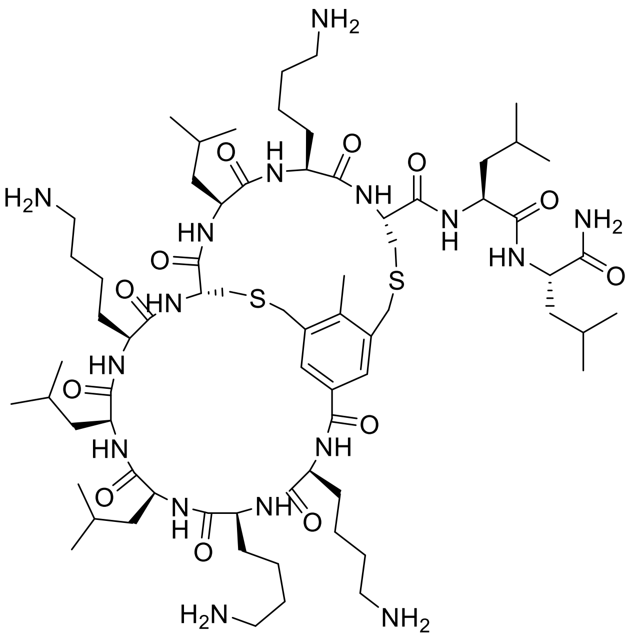

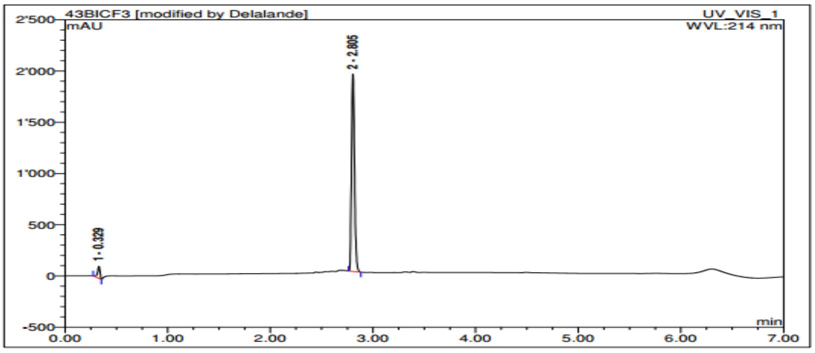

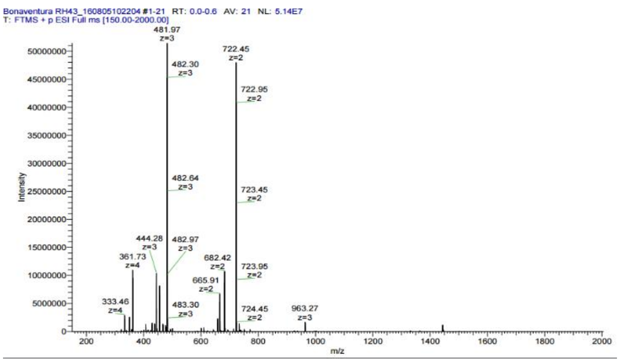

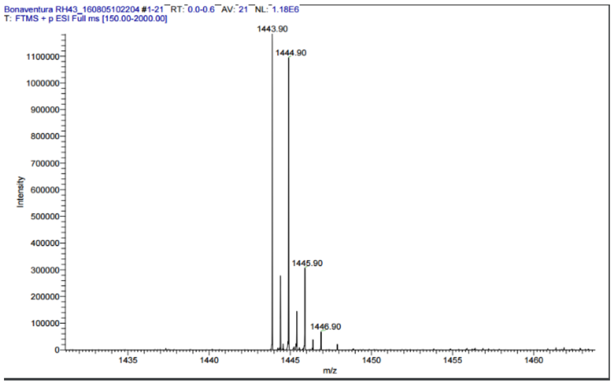

B^{12}kkllkc^{1}lkc^{2}ll (dbp65) was obtained as foamy white solid after preparative RP-HPLC (61.7 mg, 39.5 %). Analytical RP-HPLC: t_R = 2.77 min (A/D 100:0 to 0:100 in 7.00 min, λ = 214nm). MS (ESI$^+$): $C_{70}H_{122}N_{16}O_{12}S_2$ calc./obs. 1443.89/1443.89 Da $[M+H]^+$.

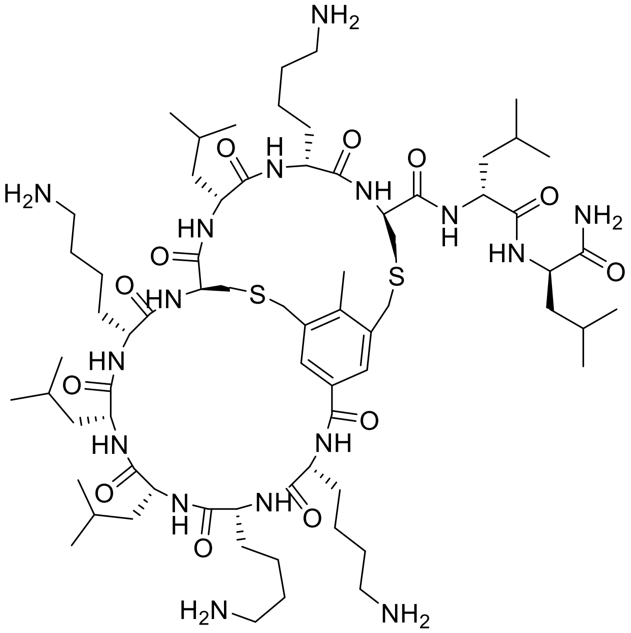

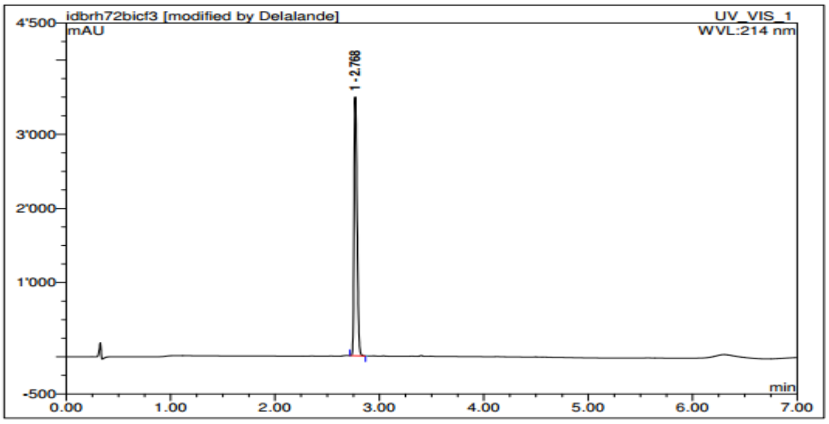

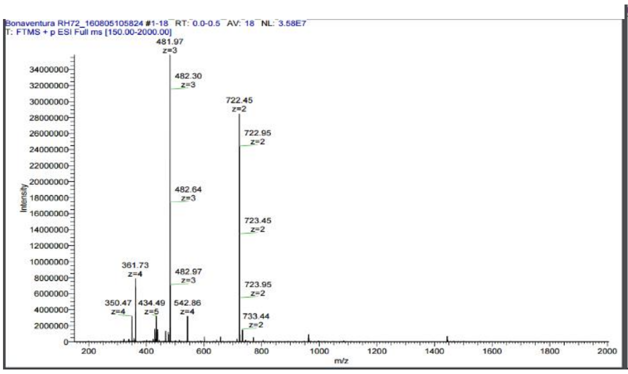

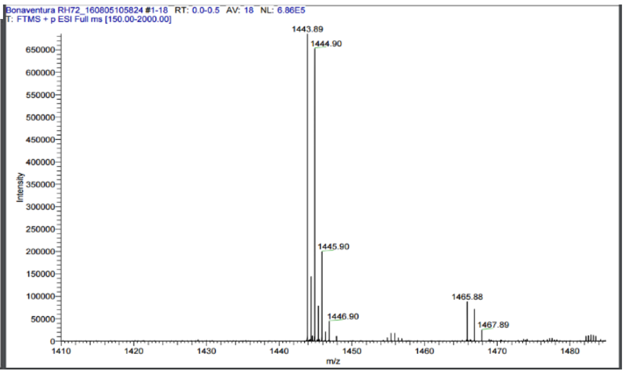

B[12]KKLC[1]KLLKC[2] (bp66) was obtained as foamy white solid after preparative RP-HPLC (41.5 mg, 26.5 %). Analytical RP-HPLC: t_R = 2.15 min (A/D 100:0 to 0:100 in 7.00 min, λ = 214nm). MS (ESI$^+$): $C_{58}H_{100}N_{14}O_{10}S_2$ calc./obs. 1217.73/1217.71 Da $[M+H]^+$.

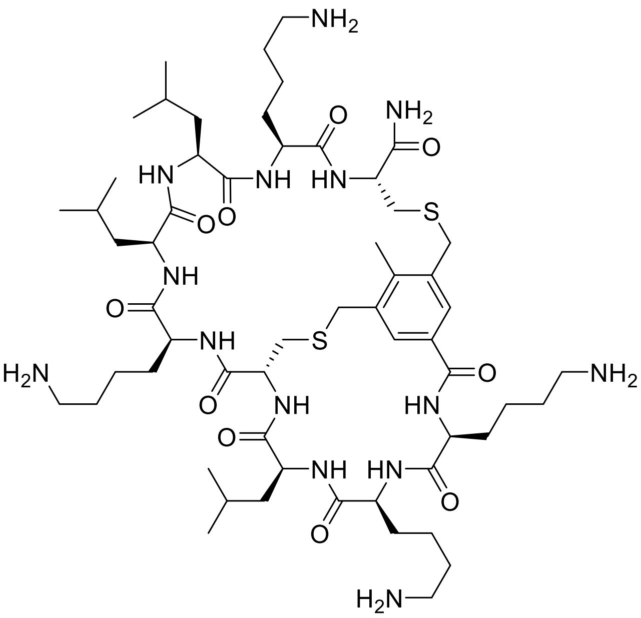

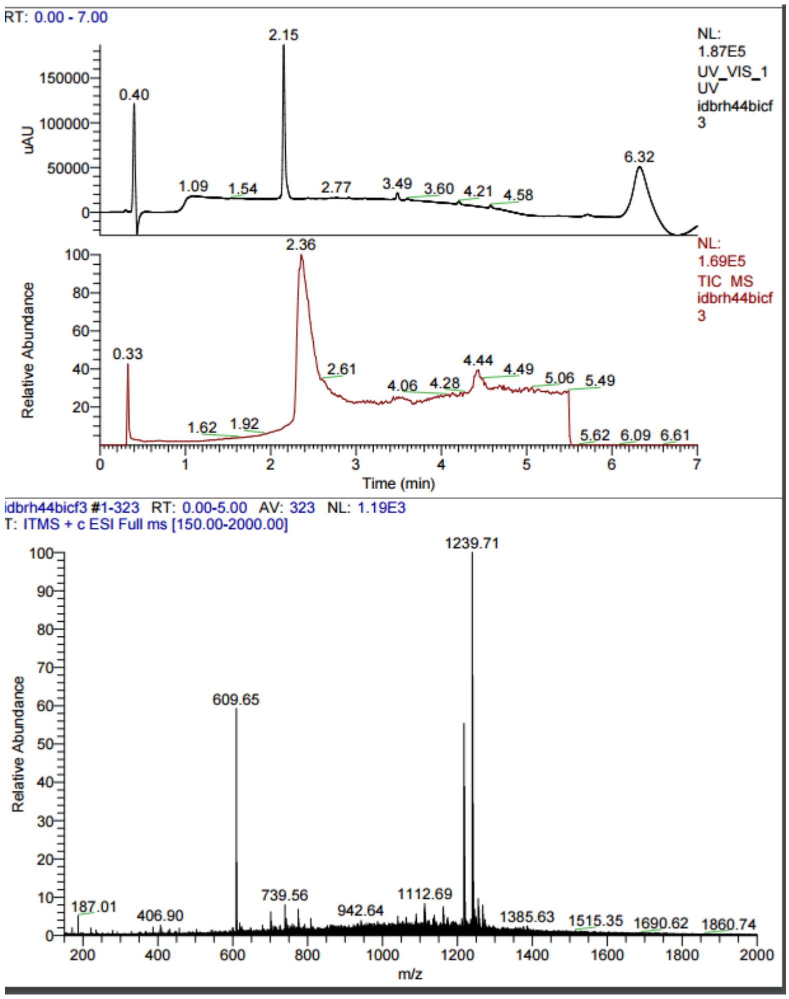

$B^{12}kKlLkC^{1}lKc^{2}Ll$ (bp67) was obtained as foamy white solid after preparative RP-HPLC (51.2 mg, 32.8 %). Analytical RP-HPLC: t_R = 2.38 min (A/D 100:0 to 0:100 in 7.00 min, λ = 214nm). MS (ESI$^+$): $C_{70}H_{122}N_{16}O_{12}S_2$ calc./obs. 1443.89/1443.89 Da $[M+H]^+$.

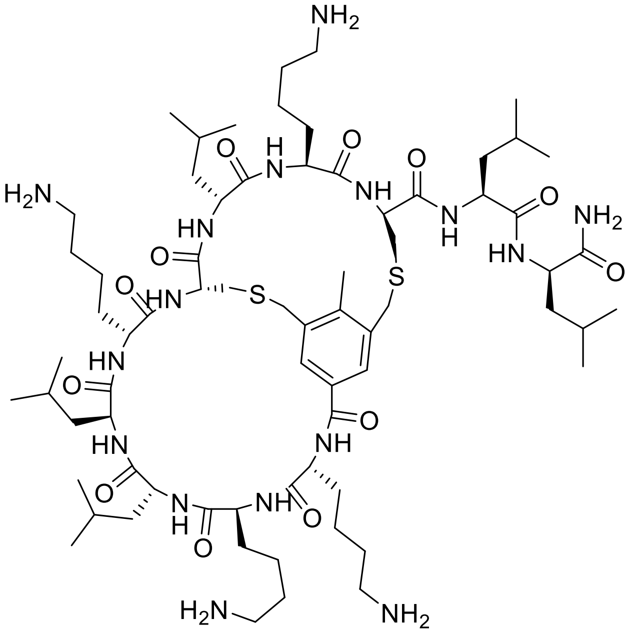

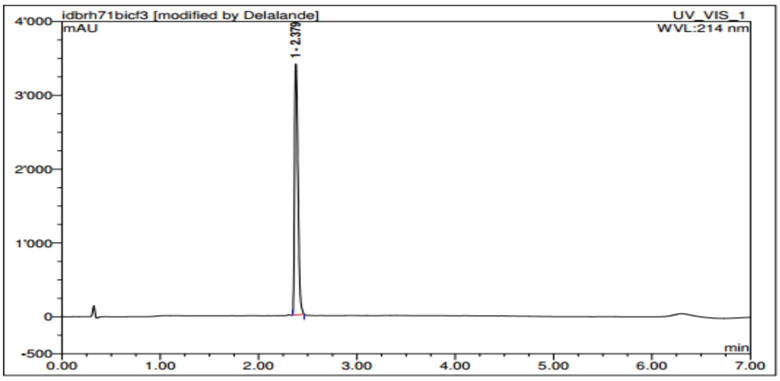

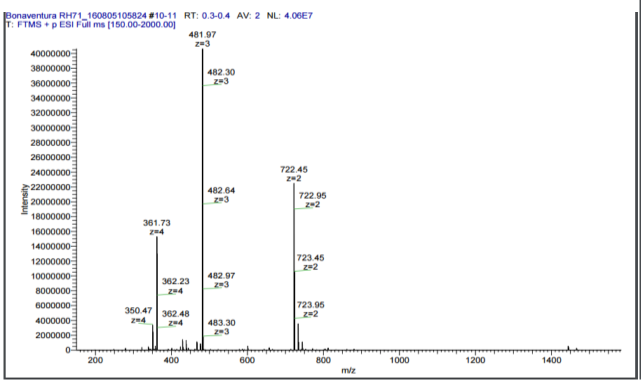

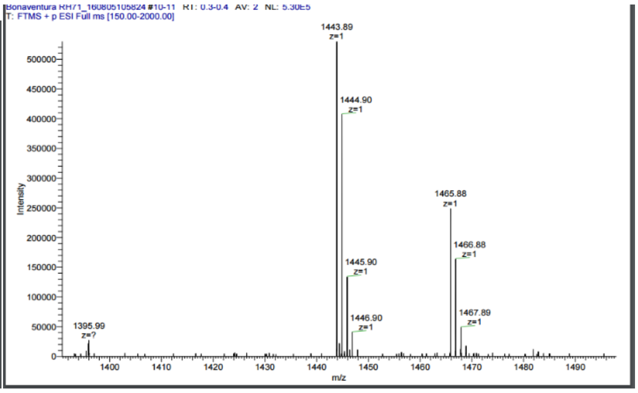

B[12]KKLLKc[1]LKc[2]LL (bp68) was obtained as foamy white solid after preparative RP-HPLC (55.7 mg, 35.7 %). Analytical RP-HPLC: t_R = 2.57 min (A/D 100:0 to 0:100 in 7.00 min, λ = 214nm). MS (ESI$^+$): $C_{70}H_{122}N_{16}O_{12}S_2$ calc./obs. 1443.89/1443.79 Da $[M+H]^+$.

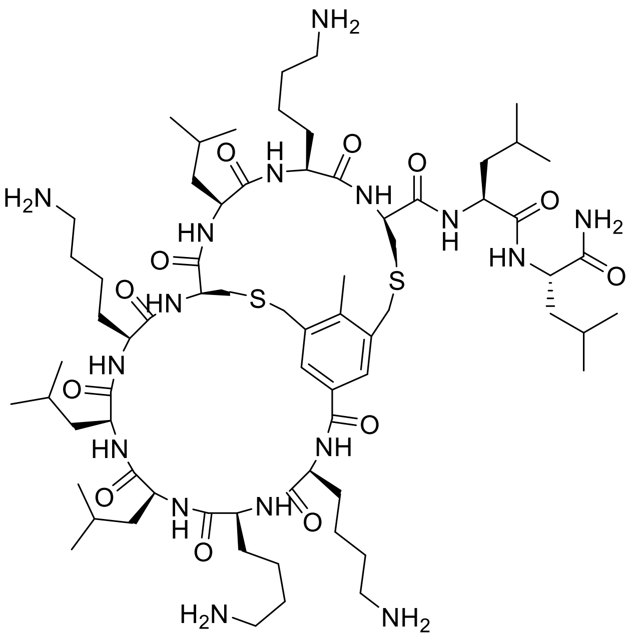

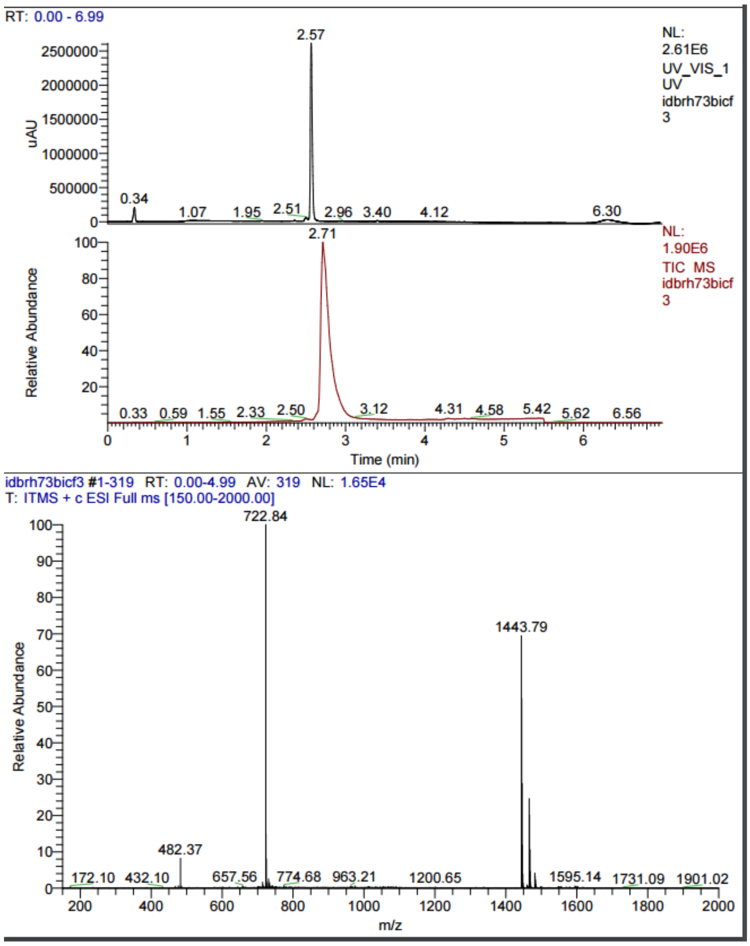

B^{12}kkLLkC^{1}LkC^{2}LL (bp69) was obtained as foamy white solid after preparative RP-HPLC (45.7 mg, 29.3 %). Analytical RP-HPLC: t_R = 1.76 min (A/D 100:0 to 0:100 in 5.00 min, λ = 214nm). MS (ESI$^+$): $C_{70}H_{122}N_{16}O_{12}S_2$ calc./obs. 1443.89/1443.83 Da $[M+H]^+$.

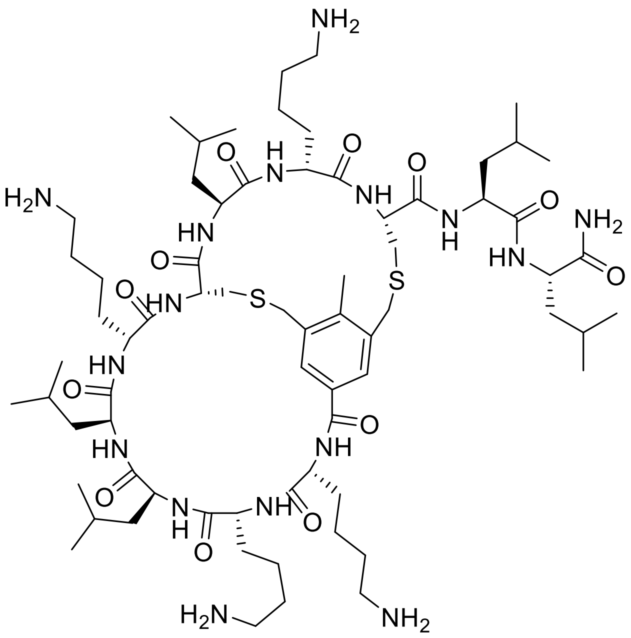

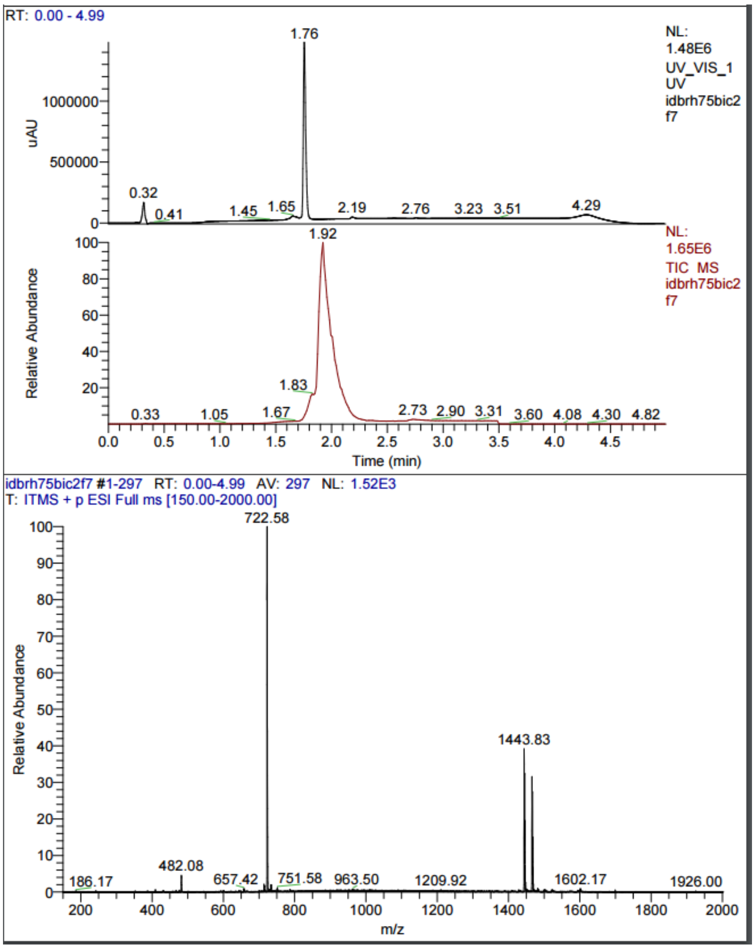

B[12]HONLYDabC[1]IRC[2]YA (bp70) was obtained as foamy white solid after preparative RP-HPLC (43.0 mg, 23.00 %). Analytical RP-HPLC: $t_R = 3.00$ min (A/D 100:0 to 0:100 in 10.00 min, $\lambda = 214$nm). MS (ESI$^+$): $C_{70}H_{101}N_{19}O_{14}S_2$ calc./obs. 1495.72/1495.72 Da [M].

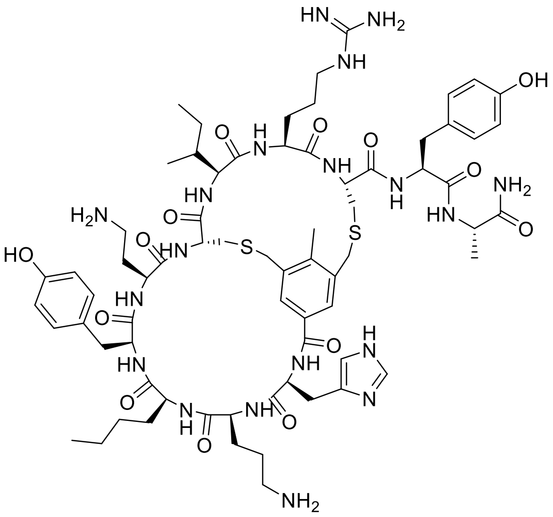

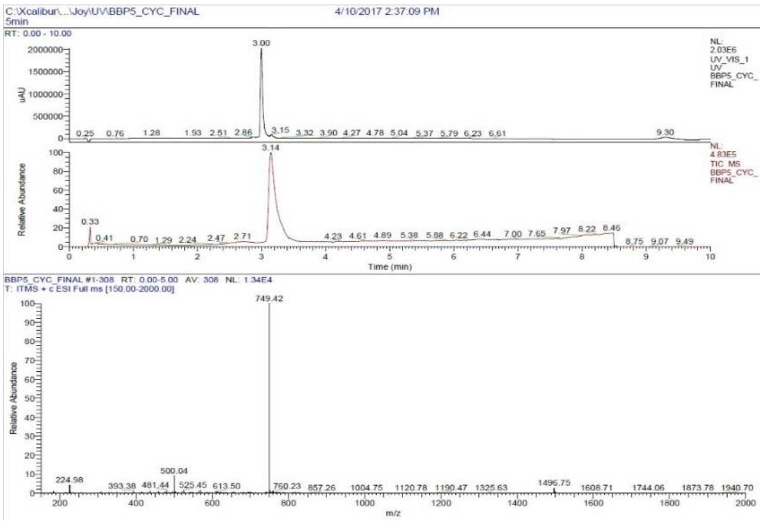

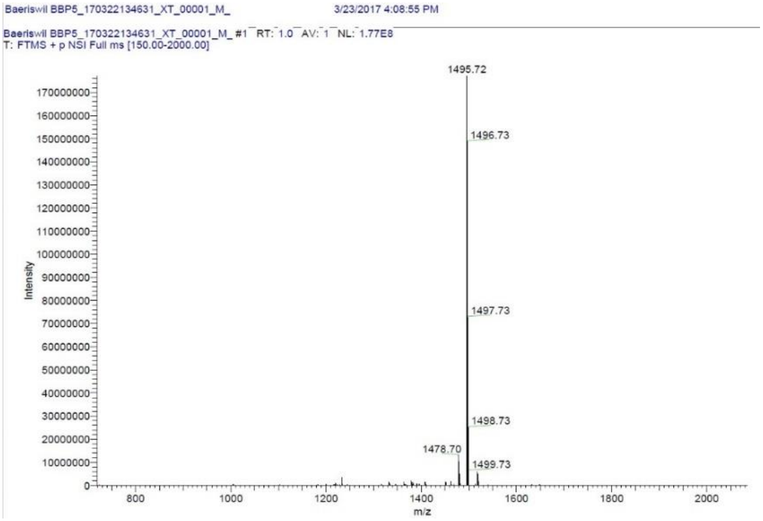

B[12]kkLLkC[1]LkC[2]LLK(*) (bp71) was obtained as foamy white solid after preparative RP-HPLC (2.0 mg, 1.1 %). * = α-L-fucosyl-acetyl group. Analytical RP-HPLC: t_R = 4.04 min (A/D 100:0 to 0:100 in 10.00 min, λ = 214nm). MS (ESI$^+$): $C_{84}H_{146}N_{18}O_{18}S_2$ calc./obs. 1759.05/1759.05 Da [M].

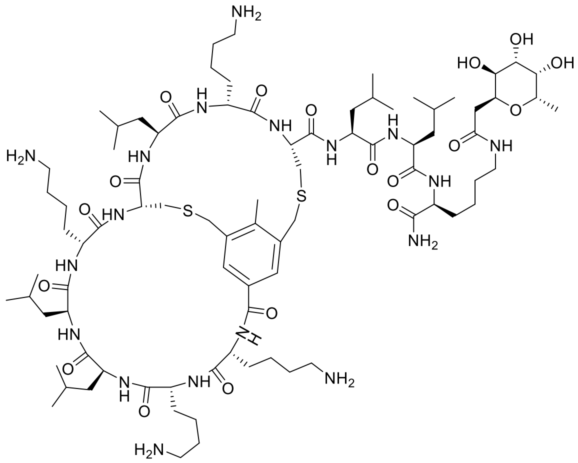

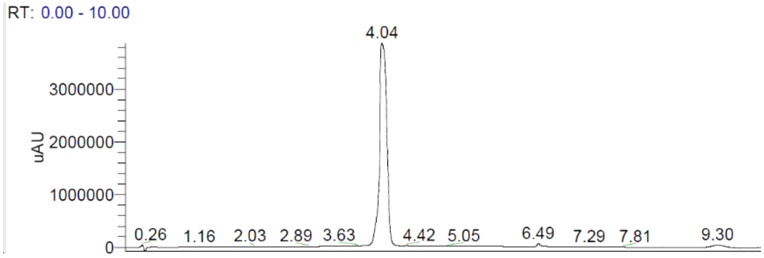

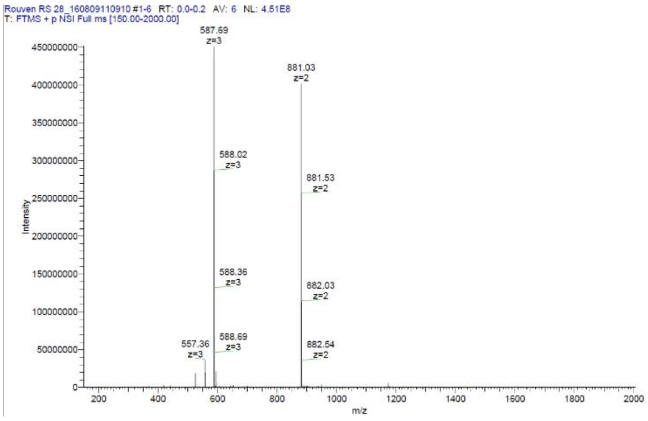

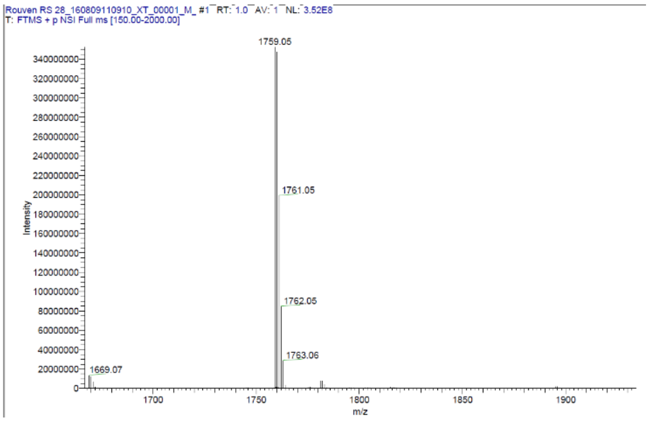

KKLLKLLKLLL (ln65) was obtained as foamy white solid after preparative RP-HPLC (84.0 mg, 58.0 %). Analytical RP-HPLC: t_R = 3.79 min (A/D 100:0 to 0:100 in 7.50 min, λ = 214nm). MS (ESI$^+$): $C_{66}H_{128}N_{16}O_{11}$ calc./obs. 1320.99/1320.99 Da [M].

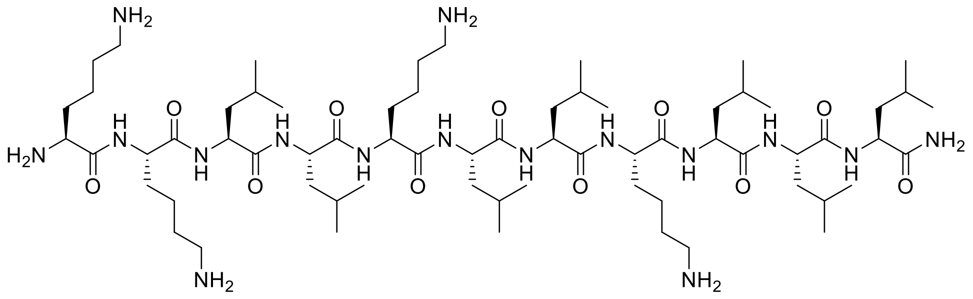

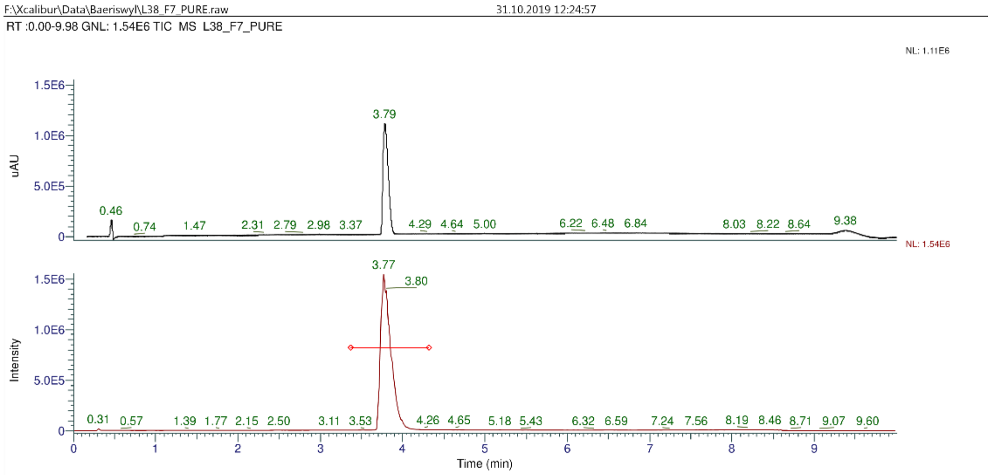

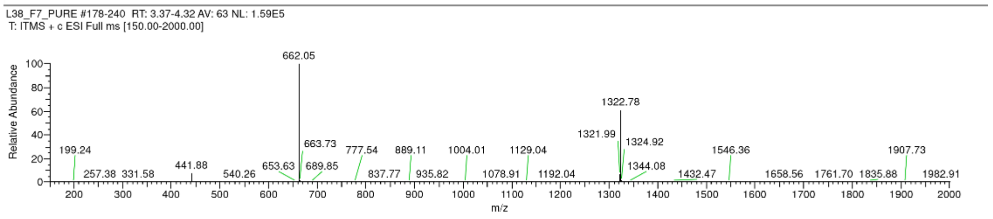

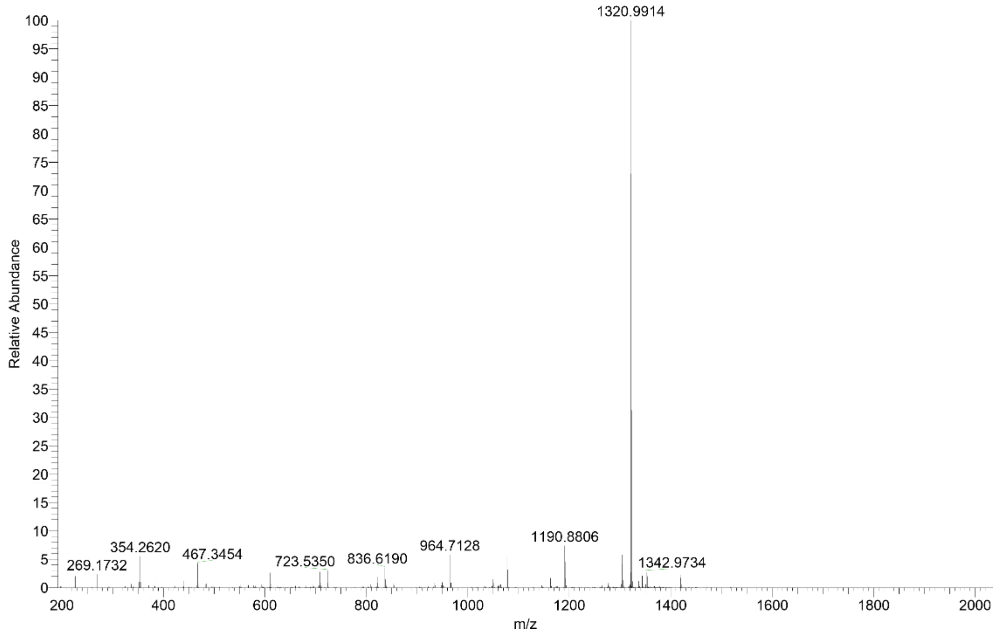

TolKKLLKC$_{m}$LKC$_{m}$LL (ln65b) was obtained as foamy white solid after preparative RP-HPLC (84.0 mg, 58.0 %). Analytical RP-HPLC: t_R = 4.56 min (A/D 100:0 to 0:100 in 7.50 min, λ = 214nm). MS (ESI$^+$): $C_{70}H_{126}N_{16}O_{12}S_2$ calc./obs. 1446.92/1446.92 Da [M].

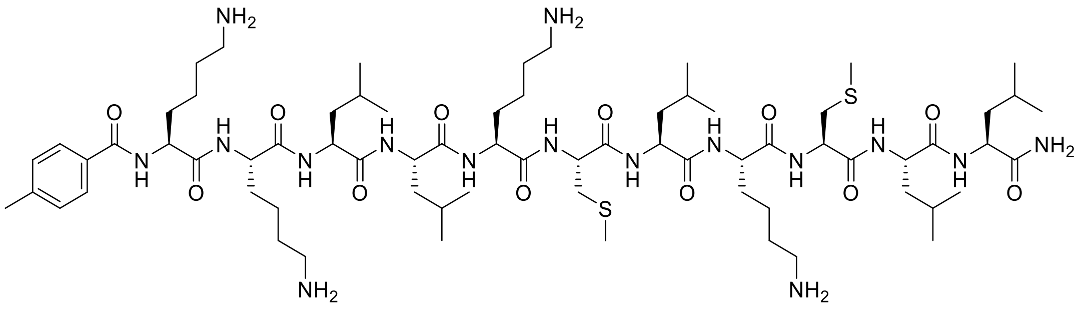

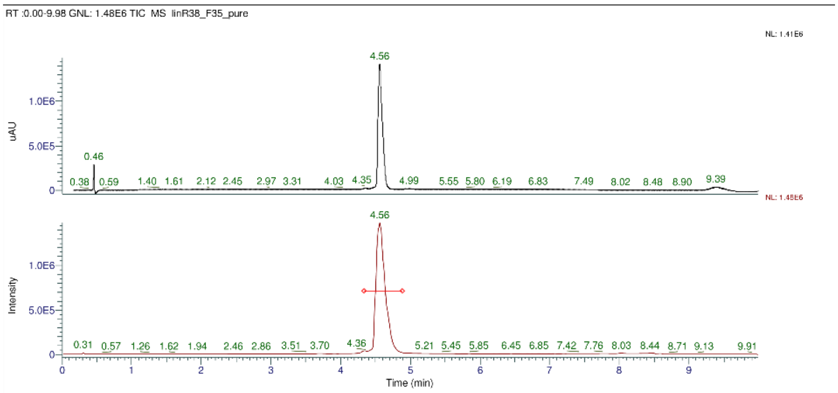

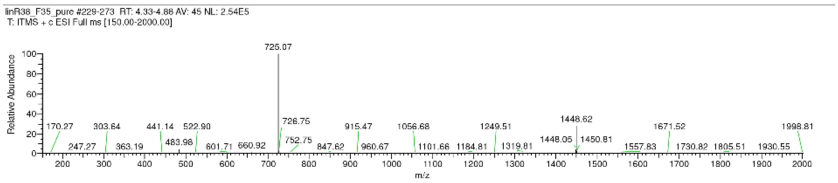

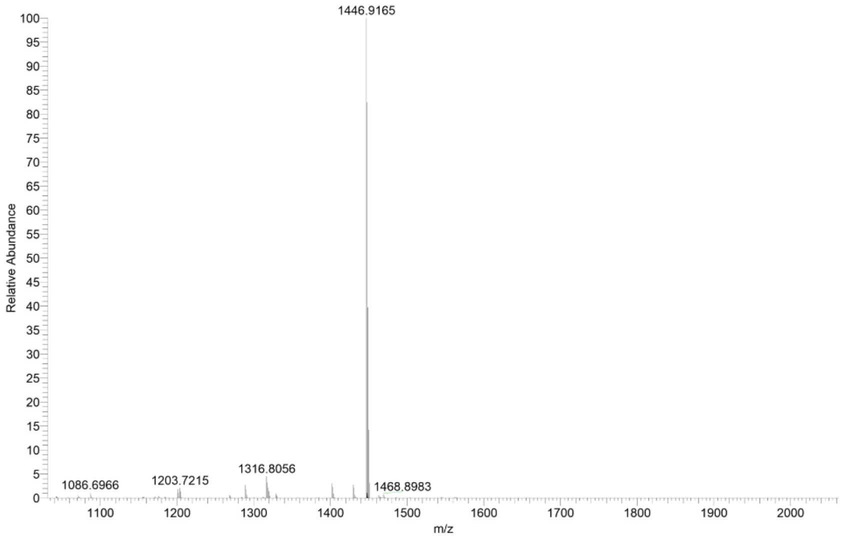

kkLLkLLkLLL (ln69) was obtained as foamy white solid after preparative RP-HPLC (102.6 mg, 70.9 %). Analytical RP-HPLC: t_R = 3.39 min (A/D 100:0 to 0:100 in 7.50 min, λ = 214nm). MS (ESI$^+$): $C_{66}H_{128}N_{16}O_{11}$ calc./obs. 1320.99/1320.99 Da [M].

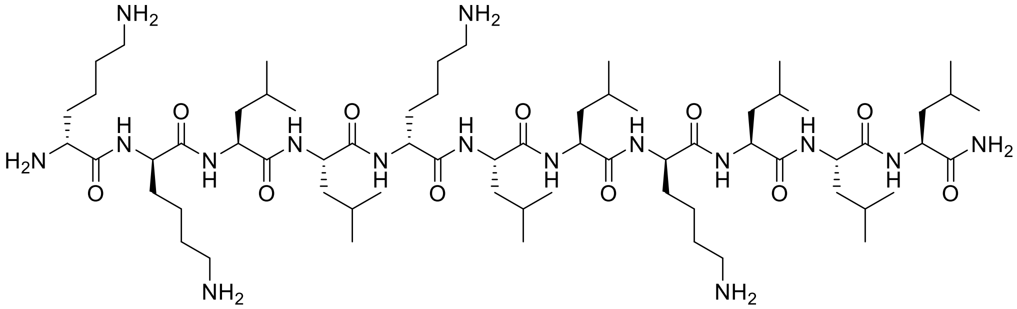

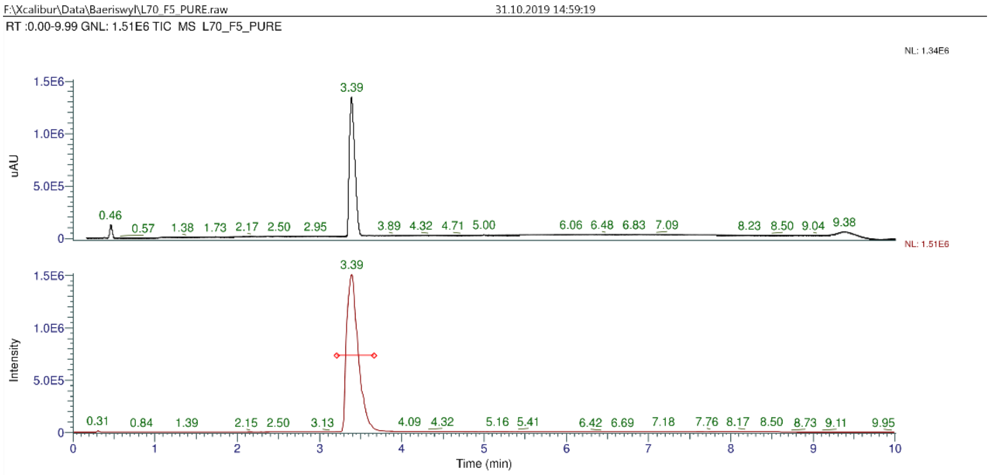

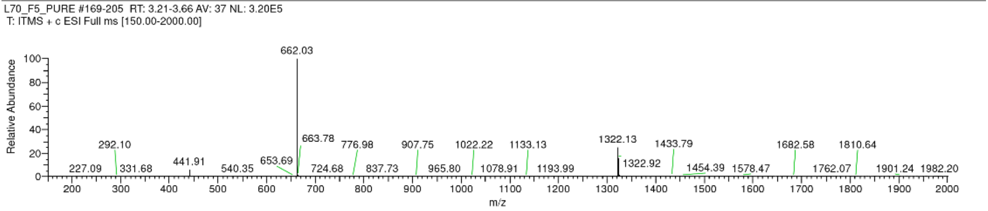

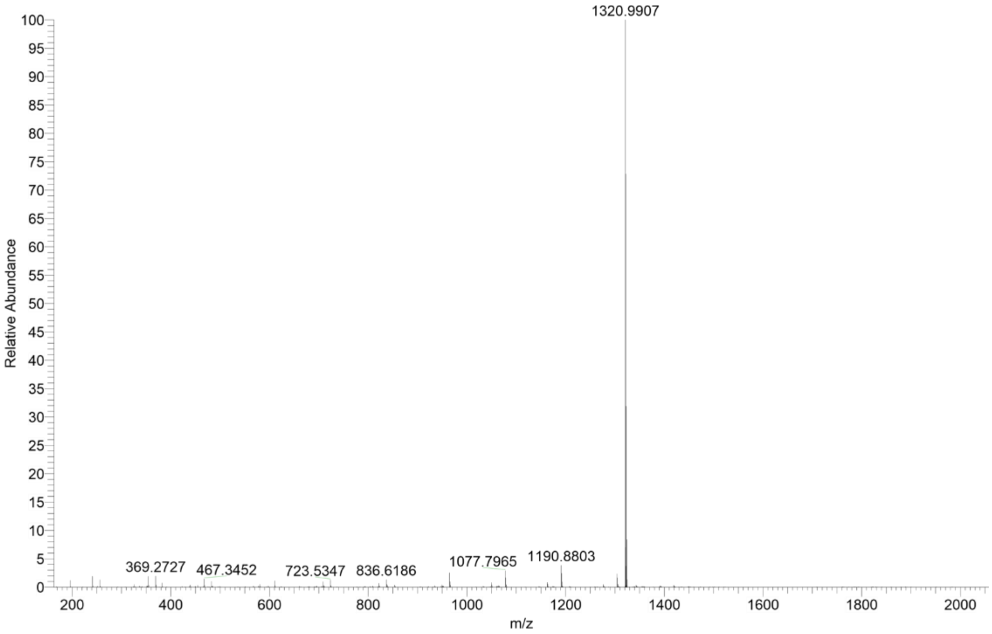

TolkkLLkC$_{m}$LkC$_{m}$LL (ln69b) was obtained as foamy white solid after preparative RP-HPLC (102.6 mg, 70.9 %). Analytical RP-HPLC: $t_R = 3.68$ min (A/D 100:0 to 0:100 in 7.50 min, $\lambda = 214$nm). MS (ESI$^+$): $C_{70}H_{126}N_{16}O_{12}S_2$ calc./obs. 1446.92/1446.92 Da [M].

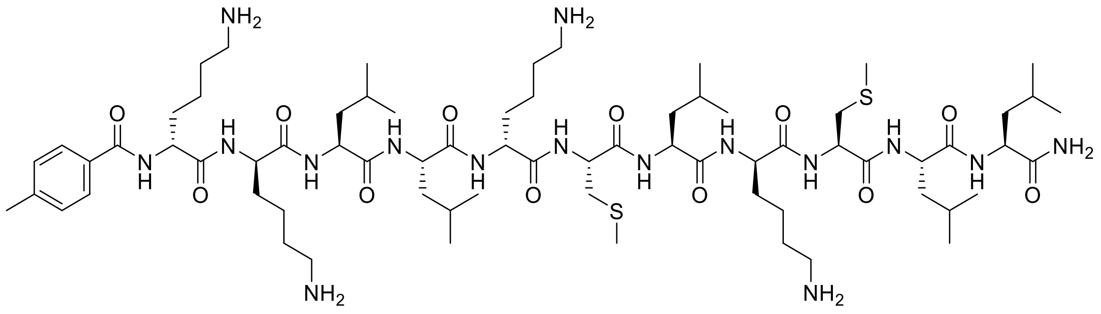

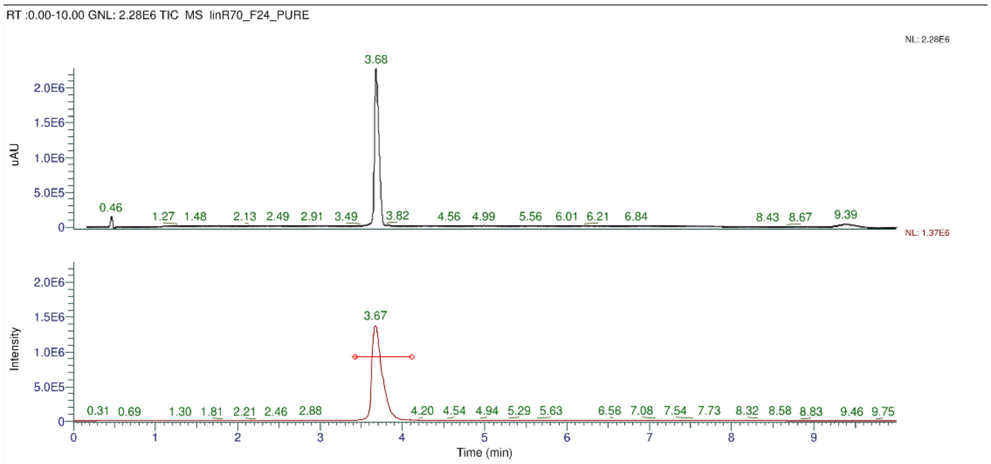

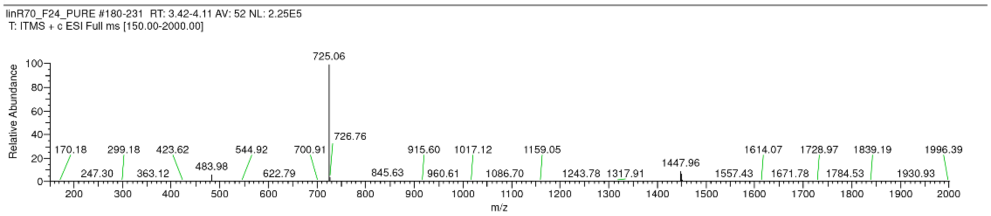

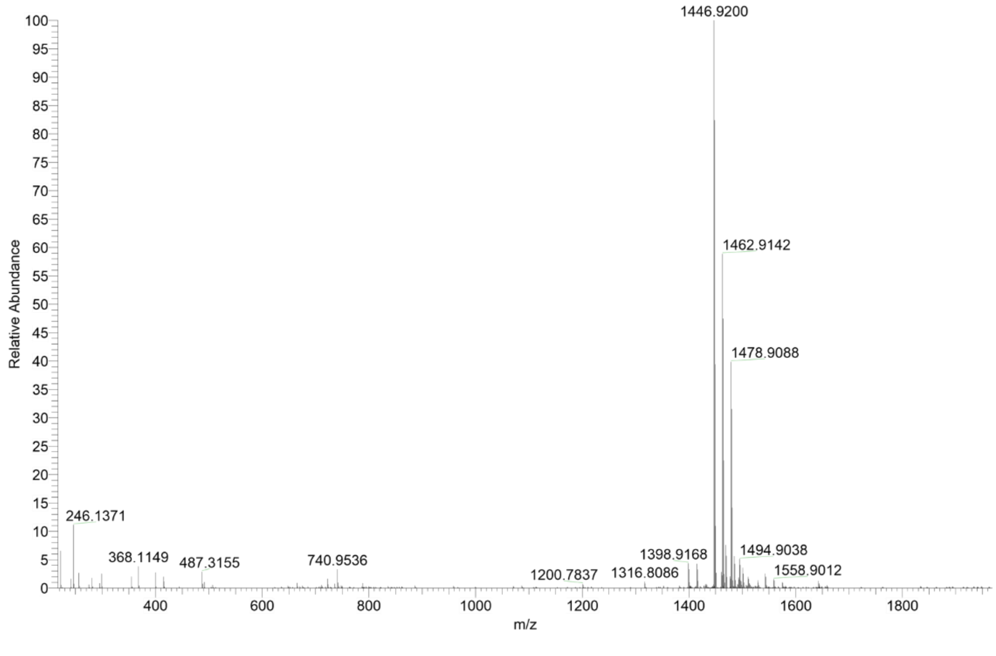

(*)kkllkllklll (Fdln65) was obtained as foamy white solid after preparative RP-HPLC (9.8 mg, 8.0 %). * = α-L-fucosyl-acetyl group. Analytical RP-HPLC: t_R = 3.95 min (A/D 100:0 to 0:100 in 7.50 min, λ = 214nm). MS (ESI$^+$): $C_{74}H_{140}N_{16}O_{16}$ calc./obs. 1510.08/1510.50 Da $[M+H]^+$.

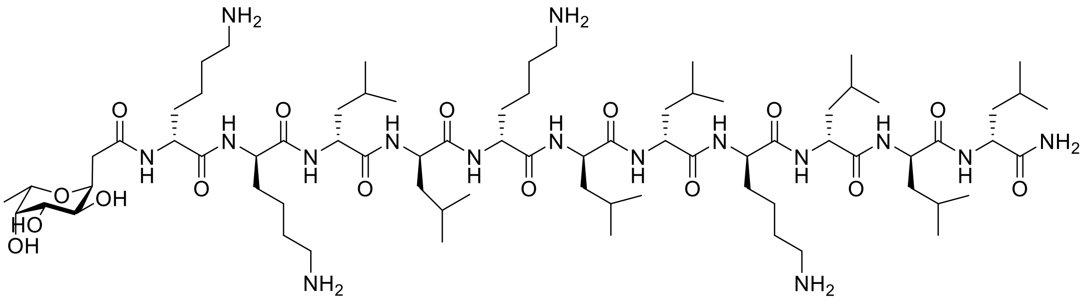

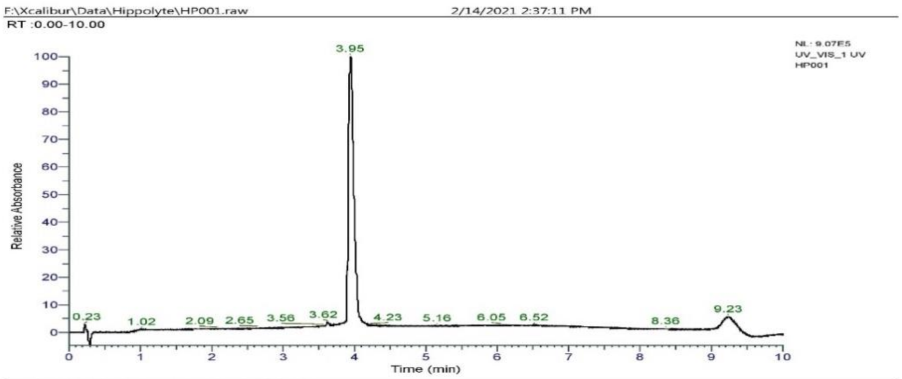

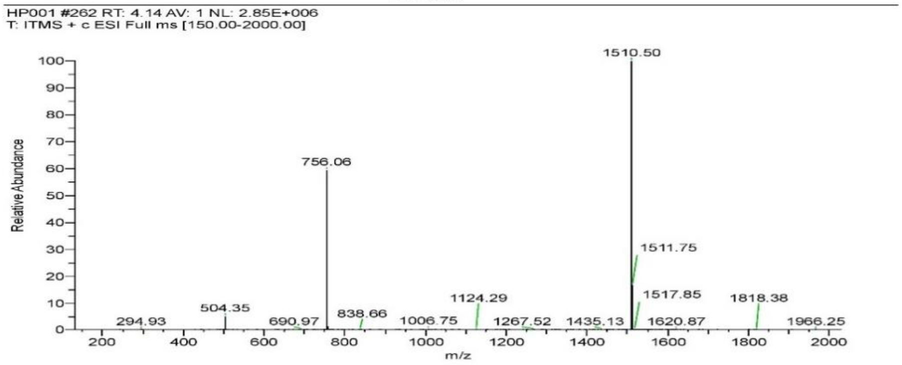

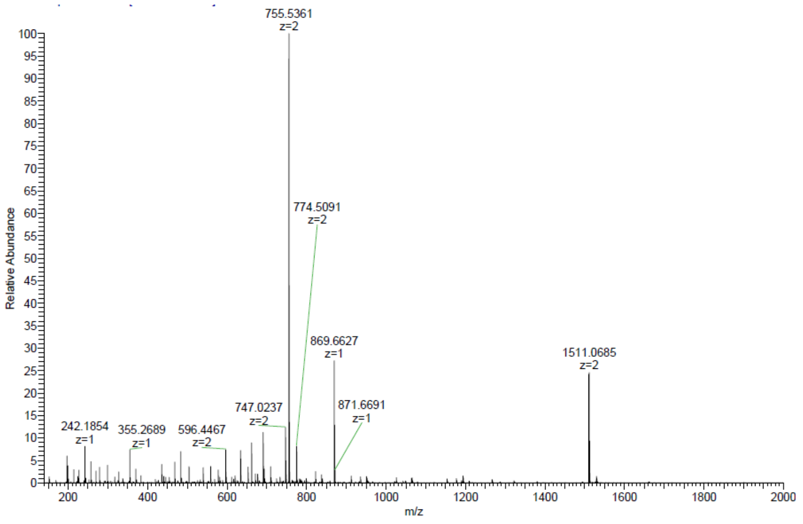

(*)KKLLKLLKLLL (Fln65) was obtained as foamy white solid after preparative RP-HPLC (21.9 mg, 11.0 %). * = α-L-fucosyl-acetyl group. Analytical RP-HPLC: t_R = 3.95 min (A/D 100:0 to 0:100 in 7.50 min, λ = 214nm). MS (ESI$^+$): $C_{74}H_{140}N_{16}O_{16}$ calc./obs. 1510.07/1510.43 Da $[M+H]^+$.

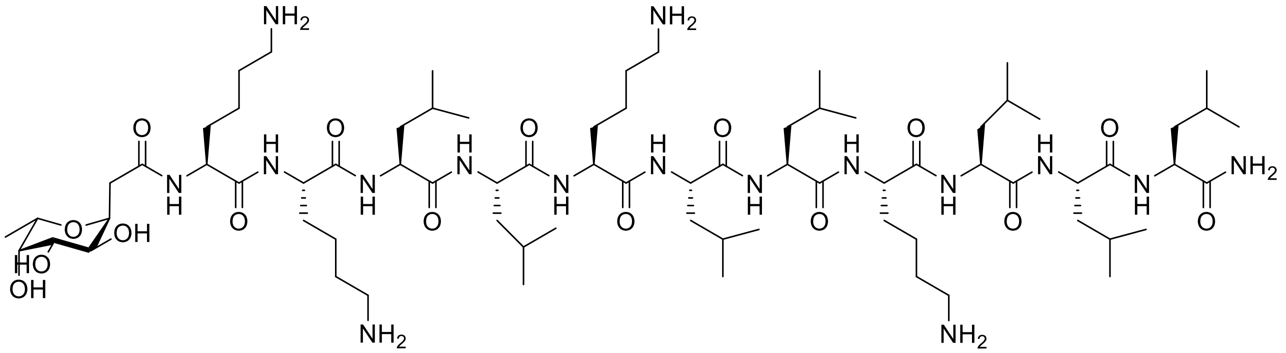

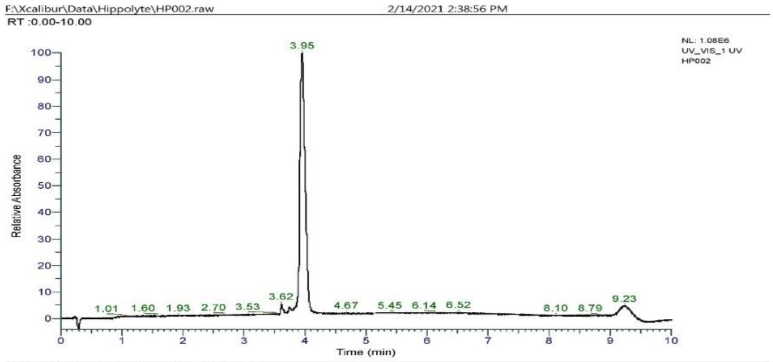

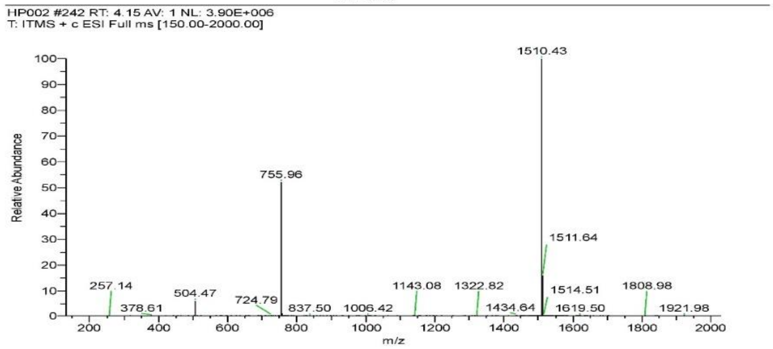

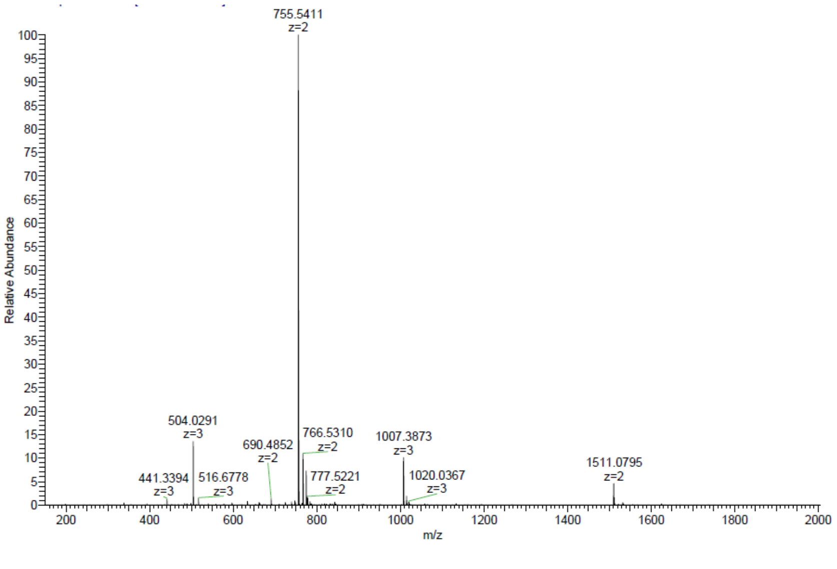

(*)kkLLkLLkLLL (Fln69) was obtained as foamy white solid after preparative RP-HPLC (38.2 mg, 19.0 %). * = α-L-fucosyl-acetyl group. Analytical RP-HPLC: t_R = 3.45 min (A/D 100:0 to 0:100 in 7.50 min, λ = 214nm). MS (ESI^+): $C_{74}H_{140}N_{16}O_{16}$ calc./obs. 1510.07/1509.89 Da $[M+H]^+$.

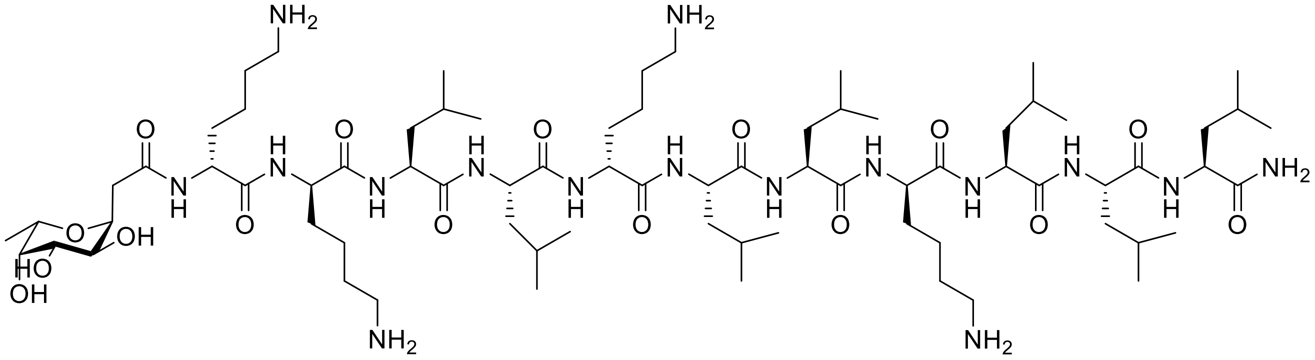

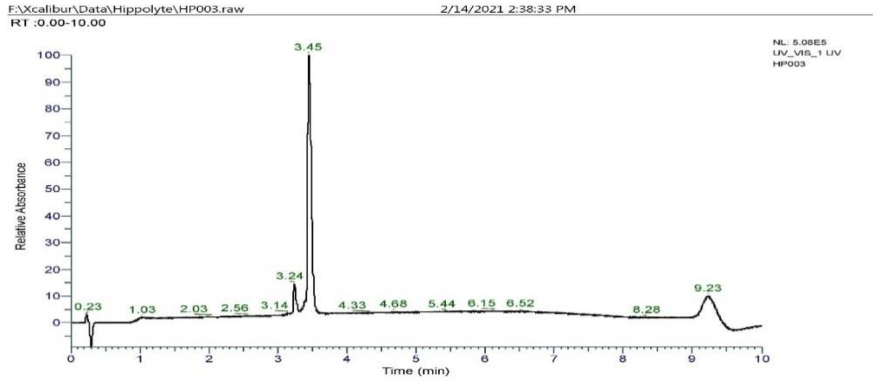

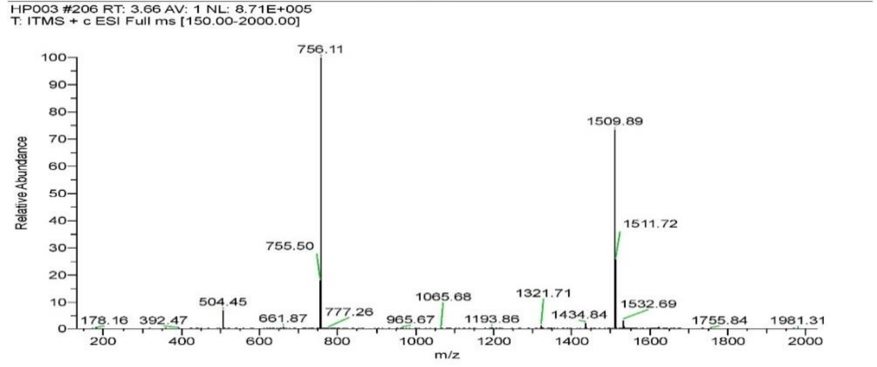

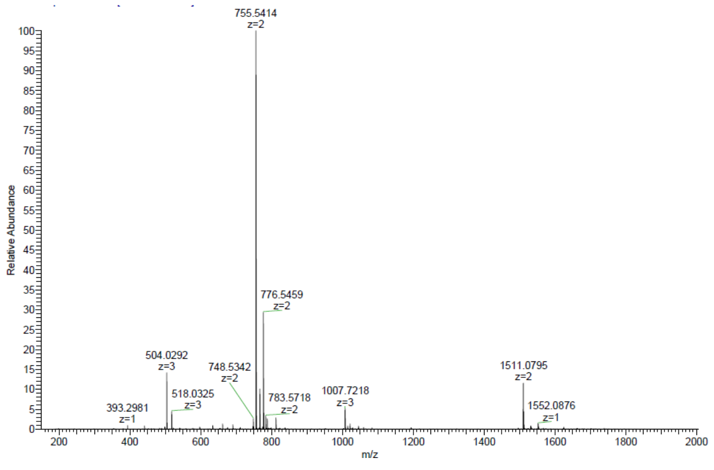

(*)KKllKllKlll (Fdln69) was obtained as foamy white solid after preparative RP-HPLC (12.9 mg, 13.0 %). * = α-L-fucosyl-acetyl group. Analytical RP-HPLC: t_R = 3.45 min (A/D 100:0 to 0:100 in 7.50 min, λ = 214nm). MS (ESI$^+$): $C_{74}H_{140}N_{16}O_{16}$ calc./obs. 1510.07/1510.08 Da $[M+H]^+$.

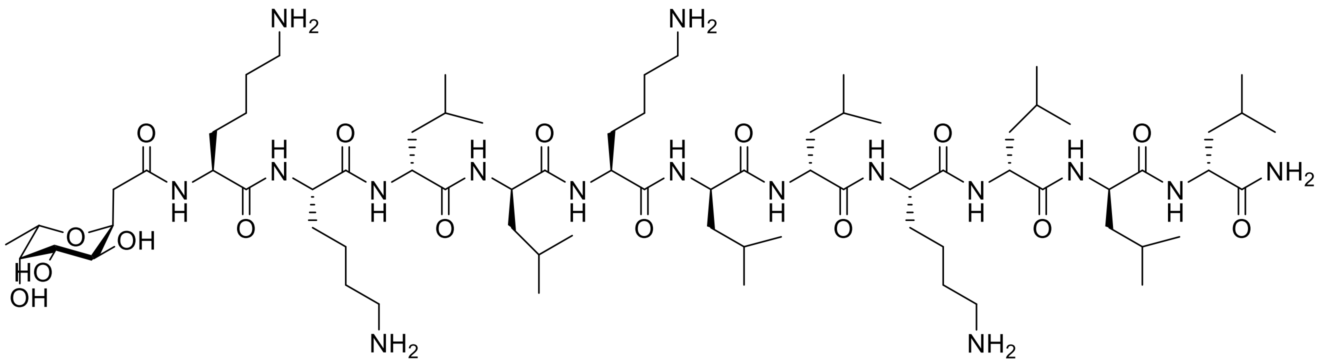

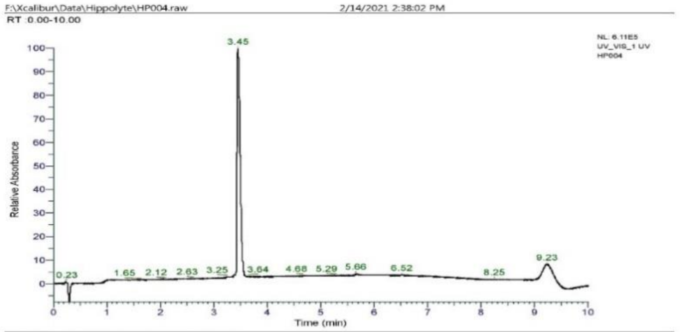

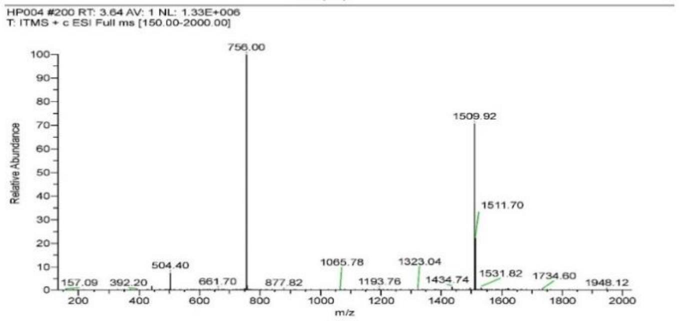

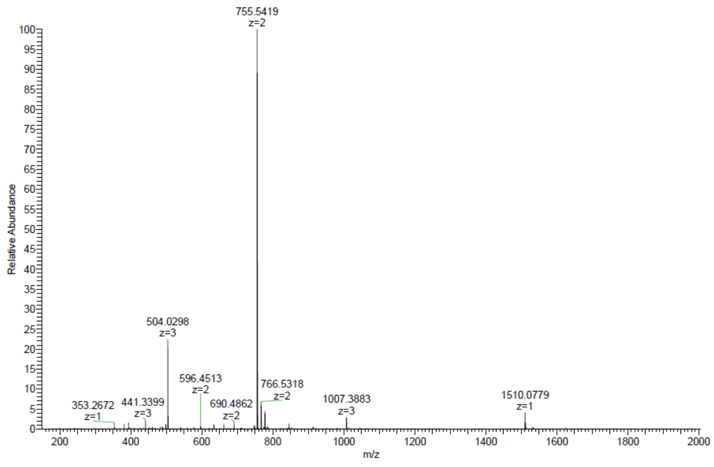

10 References

(1) Kolomiets, E.; Johansson, E. M. V.; Renaudet, O.; Darbre, T.; Reymond, J.-L. Neoglycopeptide Dendrimer Libraries as a Source of Lectin Binding Ligands. *Org. Lett.* **2007**, *9* (8), 1465–1468. https://doi.org/10.1021/ol070119d.

(2) Wiegand, I.; Hilpert, K.; Hancock, R. E. W. Agar and Broth Dilution Methods to Determine the Minimal Inhibitory Concentration (MIC) of Antimicrobial Substances. *Nat. Protoc.* **2008**, *3* (2), 163–175. https://doi.org/10.1038/nprot.2007.521.

(3) Berridge, M. V.; Herst, P. M.; Tan, A. S. Tetrazolium Dyes as Tools in Cell Biology: New Insights into Their Cellular Reduction. *Biotechnol. Annu. Rev.* **2005**, *11*, 127–152. https://doi.org/10.1016/S1387-2656(05)11004-7.

(4) Sreerama, N.; Woody, R. W. Estimation of Protein Secondary Structure from Circular Dichroism Spectra: Comparison of CONTIN, SELCON, and CDSSTR Methods with an Expanded Reference Set. *Anal. Biochem.* **2000**, *287* (2), 252–260. https://doi.org/10.1006/abio.2000.4880.

(5) Dolomanov, O. V.; Bourhis, L. J.; Gildea, R. J.; Howard, J. a. K.; Puschmann, H. OLEX2: A Complete Structure Solution, Refinement and Analysis Program. *J. Appl. Crystallogr.* **2009**, *42* (2), 339–341. https://doi.org/10.1107/S0021889808042726.

(6) Adams, P. D.; Afonine, P. V.; Bunkóczi, G.; Chen, V. B.; Davis, I. W.; Echols, N.; Headd, J. J.; Hung, L.-W.; Kapral, G. J.; Grosse-Kunstleve, R. W.; et al. PHENIX: A Comprehensive Python-Based System for Macromolecular Structure Solution. *Acta Crystallogr. D Biol. Crystallogr.* **2010**, *66* (Pt 2), 213–221. https://doi.org/10.1107/S0907444909052925.

(7) Winn, M. D.; Ballard, C. C.; Cowtan, K. D.; Dodson, E. J.; Emsley, P.; Evans, P. R.; Keegan, R. M.; Krissinel, E. B.; Leslie, A. G. W.; McCoy, A.; et al. Overview of the CCP4 Suite and Current Developments. *Acta Crystallogr. D Biol. Crystallogr.* **2011**, *67* (Pt 4), 235–242. https://doi.org/10.1107/S0907444910045749.

(8) Schrödinger, LLC. *The PyMOL Molecular Graphics System, Version 1.2r3pre*; 2010.

(9) Emsley, P.; Lohkamp, B.; Scott, W. G.; Cowtan, K. Features and Development of Coot. *Acta Crystallogr. D Biol. Crystallogr.* **2010**, *66* (Pt 4), 486–501. https://doi.org/10.1107/S0907444910007493.

(10) Kabsch, W. XDS. *Acta Crystallogr. D Biol. Crystallogr.* **2010**, *66* (Pt 2), 125–132. https://doi.org/10.1107/S0907444909047337.

(11) Abraham, M. J.; Murtola, T.; Schulz, R.; Páll, S.; Smith, J. C.; Hess, B.; Lindahl, E. GROMACS: High Performance Molecular Simulations through Multi-Level Parallelism from Laptops to Supercomputers. *SoftwareX* **2015**, *1–2*, 19–25. https://doi.org/10.1016/j.softx.2015.06.001.

(12) Oostenbrink, C.; Soares, T. A.; van der Vegt, N. F. A.; van Gunsteren, W. F. Validation of the 53A6 GROMOS Force Field. *Eur. Biophys. J. EBJ* **2005**, *34* (4), 273–284. https://doi.org/10.1007/s00249-004-0448-6.

(13) Chiu, S. W.; Clark, M.; Balaji, V.; Subramaniam, S.; Scott, H. L.; Jakobsson, E. Incorporation of Surface Tension into Molecular Dynamics Simulation of an Interface: A Fluid Phase Lipid Bilayer Membrane. *Biophys. J.* **1995**, *69* (4), 1230–1245. https://doi.org/10.1016/S0006-3495(95)80005-6.
